# Micro-total envelope system with silicon nanowire separator for safe carcinogenic chemistry

**DOI:** 10.1038/ncomms10741

**Published:** 2016-02-26

**Authors:** Ajay K. Singh, Dong-Hyeon Ko, Niraj K. Vishwakarma, Seungwook Jang, Kyoung-Ik Min, Dong-Pyo Kim

**Affiliations:** 1 National Center of Applied Microfluidic Chemistry, Department of Chemical Engineering, POSTECH (Pohang University of Science and Technology), Pohang 37673, Korea

## Abstract

Exploration and expansion of the chemistries involving toxic or carcinogenic reagents are severely limited by the health hazards their presence poses. Here, we present a micro-total envelope system (μ-TES) and an automated total process for the generation of the carcinogenic reagent, its purification and its utilization for a desired synthesis that is totally enveloped from being exposed to the carcinogen. A unique microseparator is developed on the basis of SiNWs structure to replace the usual exposure-prone distillation in separating the generated reagent. Chloromethyl methyl ether chemistry is explored as a carcinogenic model in demonstrating the efficiency of the μ-TES that is fully automated so that feeding the ingredients for the generation is all it takes to produce the desired product. Syntheses taking days can be accomplished safely in minutes with excellent yields, which bodes well for elevating the carcinogenic chemistry to new unexplored dimensions.

## Abstract (web summary)

Carcinogenic compounds present difficulties in synthesis not just during reaction but perhaps especially during purification. Here, the authors report a microfluidic device for the use of carcinogenic chloromethyl methyl ether that includes a silicon nanowire separator allowing isolated, automated purification.

## Graphical abstract

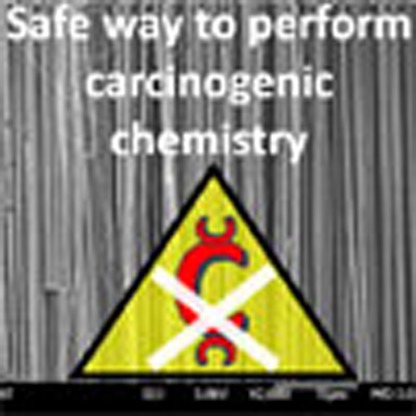

It is not uncommon that carcinogenic reagents are needed for chemical syntheses. In fact, a number of carcinogenic reagents are utilized for the synthesis of drugs and other valuable products^1^. In certain syntheses, no alternative reagents are available. A case in point is chloromethylmethyl ether (CMME). It is one of the most popular chemical reagents and has drawn the attention of many chemists during the past decades in both academia and industry^2^,^3^. It has been broadly used in multi-step syntheses of drugs and natural products, including bactericides and pesticides, and functional chemicals, solvent for polymerization reactions, as well as acid-sensitive protecting groups for alcohols, phenols, thiols and carboxylic acids^4^,^5^,^6^,^7^,^8^,^9^. In particular, chloromethylation reaction is an important intermediate step to prepare anion exchange membrane for alkaline fuel cells, desalination and electro-dialysis applications^10^.

The moisture sensitive CMME is highly carcinogenic and genotoxic by reacting spontaneously with nucleophilic DNA in the absence of enzyme, whereas even little exposure causes sore throat with fever and difficulty in breathing^11^. Despite the fact, there have been no clear-cut solutions to minimizing direct exposure to the carcinogenic reagent in CMME chemistry^2^,^12^,^13^. Therefore, scientific and industrial use of toxic CMME has been faced with serious safety issues in the synthesis, separation and transportation, which turned away many potential opportunities in this field. Thus, a safe and efficient chemical approach is needed to expand the scope of the carcinogenic chemistry, including the CMME chemistry, to new unexplored dimensions.

Continuous-flow microfluidic device has emerged as an efficient synthetic tool with attractive advantages such as high surface-to-volume ratio, and excellent mass and heat transfer, which leads to an enhancement in selectivity and a reduction in reaction time^14^,^15^,^16^,^17^,^18^,^19^,^20^,^21^,^22^,^23^,^24^,^25^. Recently, there have been several attempts to minimize the safety issues in the microfluidic processing of risky chemicals by separation through the embedded membranes^16^,^17^,^23^,^26^,^27^,^28^,^29^. However, the vulnerable polymer membrane as a physical barrier lowered diffusion rate and limited the operating conditions^30^. It is still challenging to demonstrate a zero exposure carcinogenic chemistry with excellent performance.

To realize the concept of a total process in this light, we present micro-total envelope system μ-TES), an automated total process that is enveloped totally from being exposed to carcinogen reagents. The μ-TES platform consisting of microfluidic devices enables *in situ* generation of the carcinogenic reagent, its separation from the reaction products, subsequent synthesis of the desired product with the carcinogenic reagent and decomposition of the unreacted carcinogenic reagent by quenching, separating the final desired product, all in a safe sequential manner. This μ-TES for CMME chemistry is depicted in Fig. 1, showing the four microfluidic sub-systems for generation, separation, reaction and quenching. The quenching part is elaborated in more detail later.

## Results

### *In situ* generation of CMME compound

For *in situ* generation of CMME, hexanoyl chloride and dimethoxy methane as substrates were chosen among others^11^. A single by-product of non-volatile methyl hexanoate (boiling point 151 °C) was formed in this CMME generation because of its stoichiometric yield with extensive substrate availability^11^. Two reactants in separate syringes were injected into a polytetrafluoroethylene (PTFE) capillary microreactor through a T-mixer (T_1_), and then passed through the tubing (id=300 μm, length=5.0 m) at 55 °C. Their flow rates were adjusted to maintain the 1:1 molar ratio stoichiometry (Supplementary Table 1). A back pressure regulator (BPR) was necessary to suppress the volatility of low boiling point dimethoxymethane (42 °C) reactant for a homogeneous liquid with no phase segregation. In general, a longer retention time promoted a higher CMME production. A residence time of 6 min at 40 p.s.i. was found to nearly complete the exchange reaction of dimethoxymethane to reach 98% yield of CMME (Supplementary Table 1). In contrast, a bulk CMME synthesis required 18 h of long reaction time^11^. The solvothermal-like condition in the capillary microreactor with intrinsic microfluidic advantages in mass and thermal transfer could be responsible for the accelerated synthesis.

### Fabrication of membrane-free SiNW microseparator

**Figure 2:**
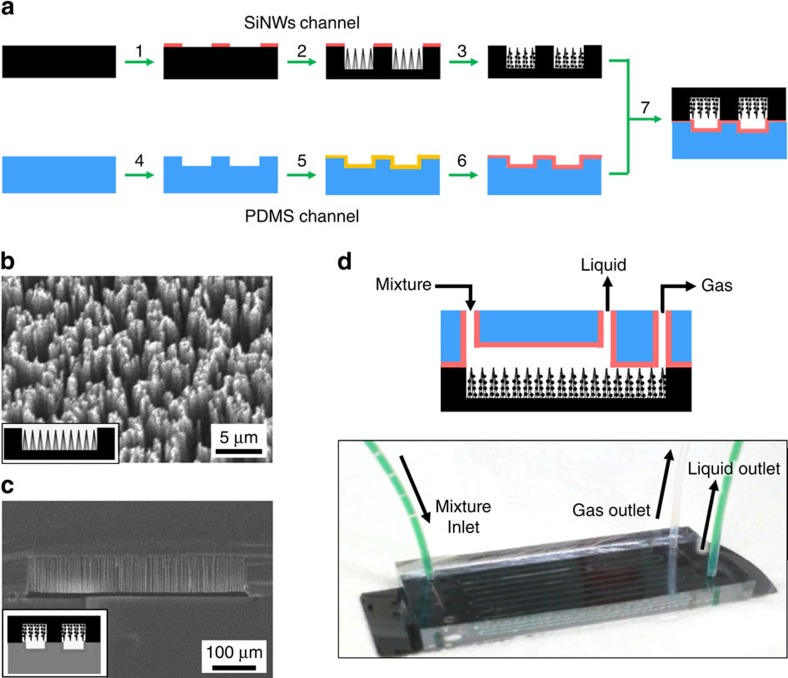
Microseparator. (**a**) Schematic illustration of fabrication of membrane-free SiNW microseparator. Step 1: patterning of AZ photoresist protective layer on Si. Step 2: preparation of SiNW pattern. Step 3: fabrication of superamphiphobic SiNWs pattern. Step 4: spin-coating polyvinylsilazane (PVSZ) on plasma-treated PDMS channel. Step 5: ultraviolet curing of PVSZ-coated channel. Step 6: thermal bonding of PVSZ-coated PDMS channel on the SiNW channel. SEM images for (**b**) cross-sectional view of SiNW microseparator, (**c**) top view of cone-shaped SiNWs (100-300 nm in diameter and 75 μm in length). (**d**) Optical image of SiNWs microseparator, showing one inlet and two outlets.

Routine purification of CMME by separation from reaction mixture invariably involves batch distillation under argon atmosphere, which poses safety and human health issues^11^. For the μ-TES, we developed a continuous-flow microfluidic device for safe and efficient purification of CMME from the *in situ* generated reaction mixture. This novel microseparator based on silicon nanowires (SiNWs) is shown in Fig. 2. The unique feature of this microseparator is that it does not require any membrane for the separation because of the definitive gas–liquid interface formed in the stable laminar flow because of the presence of SiNWs. This direct and side-by-side contact between gas and liquid phases that is rendered by simple feeding of contacting gas and liquid streams is most desirable for efficient diffusion kinetics for diverse gas separation tasks. It is definitely distinguished from the traditional gas–liquid segmented flow^31^ and microchemical distillation systems^32^ that required a low gas permeable membrane or a carrier gas (He or N_2_) at elevated temperatures.

The microseparator is essentially a long serpentine tunnel imbedded in a solid block of a polydimethylsiloxane (PDMS) slab bonded to a silicon wafer patterned by photolithography^33^. The bottom part of the tunnel is formed in the chemically resistant PDMS slab, whereas the matching upper part is filled with the cone-shaped SiNWs clusters (Fig. 2a,d) fabricated by silver-assisted selective etching of silicon wafer^33^,^34^,^35^. The cone-shaped SiNWs were decorated with SiO_2_ nanoparticles by a sol-gel process to form a hierarchical structure with rough surface. They were then fluorinated to lower the surface energy and to enhance the chemical resistivity of the SiNWs surface (see Supplementary Methods).

This SiNWs surface was found to be superamphiphobic (both superhydrophobic and superoleophobic) as revealed by the high contact angles (CAs) of water (164°) on the surface, and of three organic solvents of dimethylsulphoxide (155°), hexadecane (120°) and methyl hexanoate (105.5°). The chemical and thermal stability of the prepared superamphiphobic SiNWs surface was confirmed by exposing to corrosive gases (either HCl or NH_3_ vapour for 24 h) and thermal stress (300 °C for 1 h in air) and observing no change of CAs (Supplementary Fig. 6). The scanning electron microscopy (SEM) images in Fig. 2b,c and Supplementary Fig. 8 reveal that the diameter of Si nanowires is in the range of 100–300 nm and the length is ∼75 μm, which can be controlled by varying the etching time. The SiO_2_ nanoparticles decorating the nanowires are ∼20 nm in diameter (see Supplementary Fig. 8C,D for details). It should be pointed out here that the SiO_2_ nanotexturing on the smooth surface of cone-shaped SiNWs bundles with micrometre-scale intervals led to a hierarchically structured surface, which enhances the superamphiphobicity considerably.

To fabricate a membrane-free SiNW microseparator, a chemical-resistant polyvinylsilazane layer (ca 2–10 nm) was applied on the PDMS slab with the patterned channel structure to bond with the silicon slab, as reported elsewhere^16^. The membrane-free SiNW microseparator has one inlet for infusing a product mixture, and two outlets for separated gas and remaining liquid, respectively. The dimensions of the SiNWs channel are 500 μm in width, 70 μm in height and 43 cm in length (Fig. 2d) and those of the PDMS channel are 500 μm,15 μm and 40 cm, respectively, for the width, height and length.

### Performance of *in situ* CMME separation

The gas–liquid mixture of the products generated in the generation part of the μ-TES squirts into the microseparator through the inlet tube in bursts of CMME gas plug followed by liquid plug containing by-products. One major role of the SiNWs in the microseparator is to enable a complete separation of gas from a gas and liquid mixture. Highly superamphiphobic nature of the nanowires ensures that the liquid from the reactor does not splash into the upper SiNWs channel so that a gas phase laminar flow is established in the SiNWs channel. The other is to collect only gaseous product evaporating from the liquid phase flow in the bottom PDMS channel by establishing a definitive gas–liquid interface between the two channels. To demonstrate the stable gas–liquid laminar flow with direct interface contact, a combination of dyed water (100 μl min^−1^) and air (100 μl min^−1^) were injected through individual inlets. No bubbling was observed in the liquid flow along a 40-cm microchannel, and no dyed water was observed in the gas outlet dipped into pure methyl hexanoate (see Supplementary Movie 1). The stable gas–liquid laminar flow was maintained under various flow rates of gas and liquids (methyl hexanoate) in the range of 0.5–100 μl min^−1^. To the best of our knowledge, it is reported for the first time that a superamphiphobic surface enables handling of both liquid (organic solvent or water) and gas under dynamic flow conditions.

The separation efficiency for the generated CMME was optimized with respect to the channel dimensions, temperature and surface condition of Si nanowires. A low PDMS channel height (15 μm) relative to the height of SiNWs channel (75 μm) provided a short distance for the CMME to evaporate into the SiNWs channel. As shown in Table 1, the yield of CMME after the separation turned out to be 96% at 60 °C, which means that 98% of the CMME produced was separated because the reaction yield was 98%. Table 1 also clearly shows the beneficial effects of adding SiO_2_ nanoparticles and making the surface fluorinated, both of which render the structure more superamphiphobic. With the optimized conditions, the generated CMME was distilled quickly (3 min of residence time) at a rate of 17.5 μl min^−1^ with 98% separation efficiency. In the separation, the relatively non-volatile methyl hexanoate remains in the liquid phase.

### Total organic processes by μ-TES

**Figure 3:**
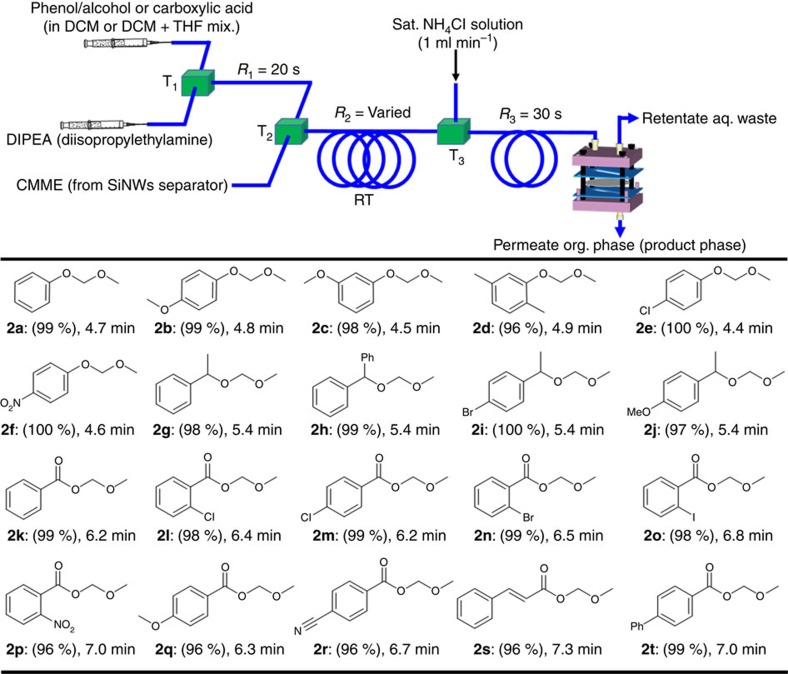
Functional group protection Expanded reaction and quenching parts in Fig. 1 for the continuous-flow μ-TES for alkoxyalkylation reactions to obtain MOM group-protected phenol, alcohols and carboxylic acid products at room temperature and ambient pressure: automated serial steps of CMME generation (6 min), CMME separation (3 min), *in situ* consumption (time in the table) and quenching/separation step (30 s). (**2a**–**2f**: phenols (1 M in DCM, flow rate 180 μl min^−1^), DIPEA (37 μl min^−1^)); alcohol products (**2g**–**2j**: alcohol (1 M in DCM, flow rate 170 μl min^−1^), DIPEA (37 μl min^−1^)) and acid products (**2k**, **2l** and **2s**: acid (1 M in DCM, flow rate 190 μl min^−1^), DIPEA (37 μl min^−1^); **2m**, **2q** and **2t**: acid (1 M in DCM+THF mixture (20:1 ratio), flow rate 180 μl min^−1^), DIPEA (37 μl min^−1^)); **2r**: acid (1 M in DCM+THF mixture (4:1 ratio), flow rate 180 μl min^−1^), DIPEA (37 μl min^−1^)). Isolated yields in parenthesis by NMR analysis were averaged by conducting three experiments at least and the data error is within ±1.5%. THF, tetrahydrofuran.

With the *in situ* generation of CMME and its separation from the reaction mixture established, it remains to demonstrate a safe CMME chemistry in the μ-TES. The protection of acid-sensitive phenol group by forming a heteroatom C–O bond was taken up first as a model reaction to utilize the purified CMME as a reactive electrophile in a serial process, which is suitable for synthesis of drug and selective functionalized chemistry^4^,^5^,^6^,^7^. For the reaction, a solution of phenol (1 M) in dichloromethane (DCM), and diisopropylethylamine (DIPEA) were separately introduced into PTFE tube through a T-shaped mixer by syringe pumps (Fig. 3). The mixed phenol and DIPEA solution was directly connected by a T-mixer (T2) into the out-flowing CMME (17.5 μl min^−1^) from the microseparator to provide a molar ratio of 1:1.15 (phenol/CMME), which was passed through a PTFE tube (id=500 μm, length=560 cm). Temperature, reagent concentration and residence time (Supplementary Table 2) were optimized to form themethoxymethyl (MOM)-phenol product. Eventually, 99% yield of (methoxymethoxy)benzene (**2a**) was obtained in 4.7 min residence time at room temperature and ambient pressure. In contrast, the conventional batch process for the identical reaction showed lower yields (61–85%) even under excess use of CMME (1.5–2 equivalent) and long reaction time (24 h) (ref. 36) at lowered temperature^37^. With the intrinsically good characteristics of microreactor, the exothermic reaction of carcinogenic CMME with DIPEA and phenol could be conducted at room temperature.

For the total process strategy, a quenching step was devised to decompose the unreacted toxic CMME as well as to remove the reaction impurities (DIPEA, its salt DIPEA·HCl) in the product mixture. This continuous process of removing toxic and waste chemicals must contribute to reducing a tedious workup step^38^ and accomplishing no exposure of carcinogenic CMME compound. In view of previous studies for quenching chemicals^2^ and removing unwanted chemicals by extraction^16^,^17^,^23^, an aqueous NH_4_Cl saturated solution (flow rate=1 ml min^−1^) was infused through a T-junction to mix with the organic flowing out of the integrated capillary microreactor, forming organic-aqueous droplets for selective extraction of reaction wastes (excess DIPEA/their salt and excess of CMME) that preferentially dissolve in water (Fig. 3) (ref. 39). The extraction between liquid droplets was accomplished in the PTFE capillary (id=500 μm, length=320 cm, *R*_3_=30 s, Supplementary Movie 2). Subsequently, the organic phase containing only the target product was separated from the aqueous phase using a PTFE membrane-embedded microseparator. The thin fluoropolymer-based PTFE membrane was preferentially wetted by the organic solvent (product) that permeated the membrane holes, whereas the non-wetting aqueous phase (waste) did not penetrate the membrane (details in Supplementary Methods) as reported in the literature^16^,^17^,^23^. Complete separation of the two phases was achieved at high flow rates (up to 300 μl min^−1^).

Various protected phenol products were obtained with excellent yields over 96% (Fig. 3, 2a–2f) in 13.9–14.4 min by the fully automated μ-TES (CMME generation (6 min), CMME separation (3 min), *in situ* consumption (4.4–4.9 min) and quenching/separation step (30 s)). In contrast, the traditional batch methods require 16 h to produce these substituted phenol products at 10.4−10.8 mmol h^−1^. The identical methodology has been further applied to protecting alcohol group with excellent yields over 97% in 5.4 min of *in situ* consumption time (Fig. 3, 2g–2j), whereas a batch process generally needed 16 h using excess (2.0–2.1 equivalent) CMME. In the case of protecting benzoic acid group, the reaction was nearly completed in only 6.2 min of *in situ* consumption time at room temperature (Fig. 3, 2k). Insoluble DIPEA·HCl salt in DCM solvent caused tube clogging problem to an extent, which required the use of a co-solvent system (DCM+THF) and controlled concentration of the carboxylic acid reactant to dissolve the precipitate. Several substituted aromatic carboxylic acids (*o*-Cl, *p*-Cl, *o*-Br, *o*-I, *o*-NO2, *p*-MeO and *p*-CN) were successfully protected in excellent yields (**2l–2q**, 96–99%) within 6.2–7 min of *in situ* consumption time under the same system and the conditions for phenol group protection. Aliphatic- and biphenyl-based carboxylic acids (**2r** and **2s**) were also successfully protected with yields up to 96% (Fig. 3), which is superior to the conventional batch process that generally required several hours with additional cooling system.

### Polymer chloromethylation by μ-TES

**Figure 4:**
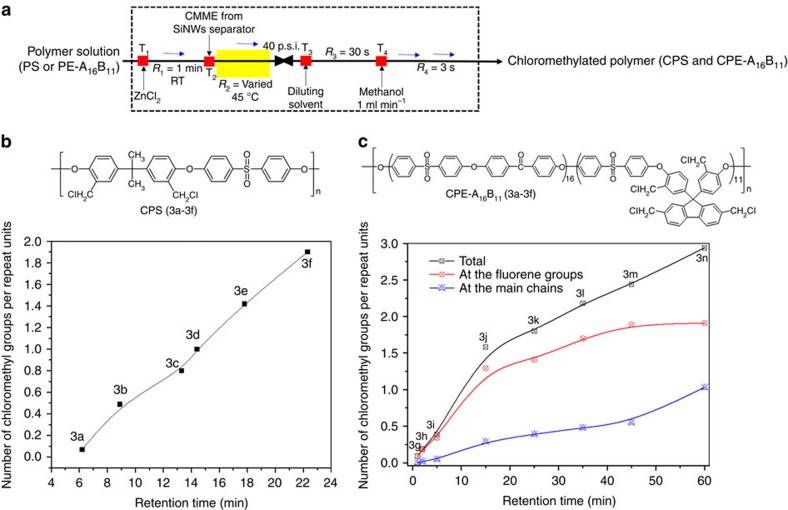
Polymer functionalization. (**a**) Continuous-flow chloromethylation by an automated μ-TES (CMME generation (6 min), distilled separation (3 min), *in situ* consumption (times in the graph) and quenching/separation step (30 s)); (**b**) chloromethylation of polysulphone polymer (CPS) reaction condition: Polysulphone (1.2 gm in 10 ml THF solution, flow rate=160 μl min^−1^), ZnCl_2_ in THF solution (0.05 M in THF, flow rate=14 μl min^−1^), CMME flow rate=17.0±0.5 μl min^−1^); (**c**) PE-A_16_B_11_polymer (1.0 g (fluorine unit=1.03 mmol) in 20 ml TCE solution (flow rate 101 ml min^−1^), ZnCl_2_ in THF solution (0.25 M, flow rate=11 μl min^−1^), CMME flow rate=17.0±0.5 μl min^−1^. The number of chloromethyl groups per repeat unit was measured by ^1^H-NMR analysis and the data error is within ±1.5%.

Moreover, an automated total process of CMME generation, separation, *in situ* consumption and quenching step was extended to the formation of carbon–carbon bond via Friedel-craft reaction that is a significant route to functionalizing aromatic polymers or anion exchange resin through electrophilic substitution^13^,^40^,^41^. The degree of chloromethylation is very critical to the ion exchange capacity of polymer membrane, which is always challenging for the membrane scientists^42^. Polysulphone polymer (containing aromatic backbone) was chosen as a model substrate for Friedel-craft chloromethylation (Fig. 4). A solution of polysulphone (in THF) and ZnCl_2_ (in THF) was mixed at a T-junction, which was then mixed at another T junction with the purified CMME from the membrane-free SiNW microseparator (see Fig. 4a and Supplementary Fig. 15 for more details). The reaction mixture was then introduced into a capillary microreactor system with endpoint BPR, which was submerged in a preheated oil bath at different temperatures (30–45 °C), and back pressures (40 p.s.i.). The reaction time of CMME ranged from 1 to 22 min with ZnCl_2_ catalyst (0.25 M in THF). In general, the degree of chloromethylation (based on NMR analysis) gradually increased with longer reaction time (Fig. 4b, **3a**–**3e**), eventually reaching 1.90 without gelation at the optimal conditions (40 p.s.i. BPR, 40 equivalent CMME concentration, 45 °C and 22 min chloromethylation, **3f**) by preferably functionalizing the electron providing propane-2,2-diylgroup containing unit. In contrast, the conventional batch process yields a lower degree of chloromethylation (1.69) even with longer reaction time (90 min) at high temperature (75 °C) (ref. 20). The quenching/separation of the unreacted CMME by extraction through methanol-aqueous droplets was conducted in a continuous-flow device to reduce manpower and exposure of unreacted CMME chemicals.

Recently, aromatic multiblock copolymers have drawn interest as a new class of polymeric anion exchange membrane because the degree of chloromethylation could be highly improved^43^. The long process time over 144 h, however, could increase the risk of carcinogenic exposure to manpower. Therefore, the μ-TES was applied to chloromethylate a synthesized poly(arylene ether) block copolymer (PE-A_16_B_11_; details in Supplementary Methods)^43^. As shown in Fig. 4c and Supplementary Fig. 19, the degree of chloromethylation in the A unit of PE-A_16_B_11_ block copolymer could not exceed 1.0 for 60 min *in situ* reaction time at 45 °C with 80 equivalent of CMME, because electron-withdrawing ketone and sulfone groups were less reactive with electrophiles. In contrast, the fluorene groups of the B unit in the PE-A_16_B_11_ block copolymer were more reactive to be chloromethylated, considerably more at preferable positions (2 and 7) due to the absence of electron-withdrawing group^43^.

## Discussion

We have developed a μ-TES and established a zero exposure of carcinogenic reagent for CMME chemistry using the μ-TES. This autonomous serial process of CMME generation, self-purification, separation, reaction and quenching system does not require any additional workup and column chromatography, which completely remove the safety issues involving risky compounds in the chemical processes. In particular, a novel membrane-free SiNWs microseparator developed here has been shown to allow for the separation of low boiling chemicals by simple heating in a continuous-flow manner. This total process concept including the integrated system and procedure can be easily extended to other carcinogenic, explosive, toxic or noxious regents. More importantly, the system provided here would provide a safe and fast avenue for those potential workers who turned away or using alternative routes due to the safety and longevity issues in the areas of drug discovery, natural products, ion-exchange membranes, materials synthesis and biology.

## Methods

### General

Used material details given in Supplementary Methods. GC/MS spectrum was recorded by Agilent 5975C GC/MSD System (Agilent Technologies). ^1^H NMR and ^13^C NMR spectra were recorded on a Bruker 600 or 300. Field emission scanning electron microscope (FE-SEM) images were obtained using JSM-6700F. The synthesized product compounds were fully characterized by their Mass-spectra, ^1^H and ^13^C NMR data, by collecting for 30 min unless otherwise noted. Optimization yields were average of at least two experiments. Electron ionization mass spectra were recorded on 5675C VL MSD spectrometer (Agilent Technologies). CAs were measured using a SmartDrop (FemtoFab). The etched fraction of SiNWs was analysed by calculating the ratio of etched area to the whole area of SiNWs from the SEM image through Image J software.

### Typical procedure for total organic process

A solution of substituted organic compound in DCM or THF+DCM mixture and excess DIPEA reactant were introduced to a T-mixer (T_1_) of molar ratio of 1:1.15 to maintain reaction stoichiometry, and then passed through a PTFE tubing (id=500 μm, length=varied) for varied retention time. Properly mixed organic compound and DIPEA solution were connected by another T-mixer (T_2_) and directly to outlet of the membrane-free SiNW microseparator (see in Supplementary Methods in more details). Out-flowing CMME (17.5 μl min^−1^) and the substrate were controlled to become molar ratio 1:1.15 (organic compound/CMME). The three components (phenol, DIPEA and out-flowing CMME) were mixed through T-junction (T_2_; see Supplementary Fig. 10) and infused to PTFE tubing (id=500 μm, length=varied) for varied time. Note that the tube length was varied with different retention times of chloromethylation. The organic group protection setup as aforementioned in the Supplementary Fig. 10 was connected to a quencher inlet for *in situ* decomposition of CMME and extraction removal of excess DIPEA/their salt. The saturated aqueous NH_4_Cl quenching solution was merged to the DCM-based reaction mixture by T-junction to form aqueous-organic droplets (see Supplementary Fig. 11 for more details). Sufficient quenching and extraction from the above reaction mixture was observed at 1 ml min^−1^ flow rate of aqueous NH_4_Cl. To separate organic phase containing the wanted product from the aqueous impurity phase, the additional PFFE membrane embedded separator was added to end of the integrated microreactor system, as similarly reported (see Supplementary Fig. 12 for more details).

### Typical procedure for polymer chloromethylation

A solution of polymer (see Supplementary Methods in more details) was taken in one syringe and the solution of ZnCl_2_ (varied) in THF was taken in another syringe (see Supplementary Figs 15 and 19 in more details). The two solutions were introduced to a T-mixer (T_1_) in a flow rate (detail in Supplementary Methods), and then passed through PTFE tubing (id=500 μm, length=varied) to mix for varied residence time. Next the reaction mixture was connected to outlet of CMME purified microreactor at a flow rate=17.5 μl min^−1^ by another T-mixer (T_2_) at 45 °C (Supplementary Figs 15 and 19 in more details). Three component mixture (polymer, ZnCl_2_, CMME) was passed through a PTFE tubing (id=500 μm, length=varied as mentioned in Supplementary Figs 15 and 19) for different retention times. The use of 40 p.s.i. BPR enhanced the degree of chloromethylation. Then controlled degree of chloromethylated polymer solution was connected with T-mixer (T_3_), and THF/1,1,2,2-tetrachloroethane (TCE) solvent was added continuously (flow rate varied) and again passed through PTFE tubing (id=500 μm, length=varied) to dilute the polymer solution for varied time. The properly diluted chloromethylated polymer solution was precipitated continuously by adding methanol (95% methanol and 5% water) solution (flow rate=1 ml min^−1^) through T-mixer (T_4_) and passed through a PTFE tubing (id=1,000 μm, length=10 cm) to complete quenching of excess of ZnCl_2_ and CMME during 3 s residence time with no clogging problem. Finally, the chloromethylated polymer was washed several times with de-ionized water to determine degree of chloromthylation by NMR analysis after drying.

## Additional information

**How to cite this article:** Singh, A. K. *et al.* Micro-total envelope system with silicon nanowire separator for safe carcinogenic chemistry. *Nat. Commun.* 7:10741 doi: 10.1038/ncomms10741 (2016).

## Supplementary Material

Supplementary Information Supplementary Figures 1-73 and Supplementary Tables 1-2, Supplementary Methods and Supplementary References

Supplementary Movie 1 Gas-liquid separation through SiNWs embedded microseparator

Supplementary Movie 2 Aquous-organic separation through PTFE membrane embedded separator

## Figures and Tables

**Figure 1:**
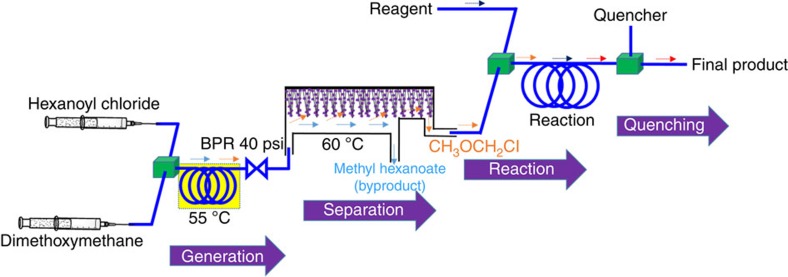
Continuous-flow μ-TES. for safe utilization of carcinogenic chloromethyl methyl ether (CMME) via generation of CMME, separation, *in situ* consumption for forming a final product and quenching step.

**Table 1:** Continuous-flow separation performance of the membrane-free SiNW microseparator for the *in situ* synthesized CMME compound.

**Entry**	**Hexanoyl chloride flow rate (μl** **min**^−**1**^**)**	**Dimethoxy methane flow rate (μl** **min**^−**1**^**)**	**Temperature (°C)**	**CH**_**3**_**OCH**_**2**_Cl yield (%)*
1	36	22	60	96
2	36	22	55	70
3	36	22	50	2
4†	36	22	60	88
5‡	36	22	60	75

CMME, chloromethylmethyl ether; SiNW, silicon nanowire.

^*^Isolated yield is based on NMR analysis, retention time 3 min

^†^Without SiO_2_ nanoparticle coating on SiNWs.

^‡^Without trichloro(1H, 1H, 2H, 2H-perfluorooctyl)silane coating.
