# Supplementary material for: Micro-total envelope system with silicon nanowire separator for safe carcinogenic chemistry
Source: Nat Commun. 2016 Feb 26;7:10741. doi: 10.1038/ncomms10741 (PMC4773427; doi:10.1038/ncomms10741)
Supplement: Supplementary Information — Supplementary Figures 1-73 and Supplementary Tables 1-2, Supplementary Methods and Supplementary References [file ncomms10741-s1.pdf]

Supplementary Figures

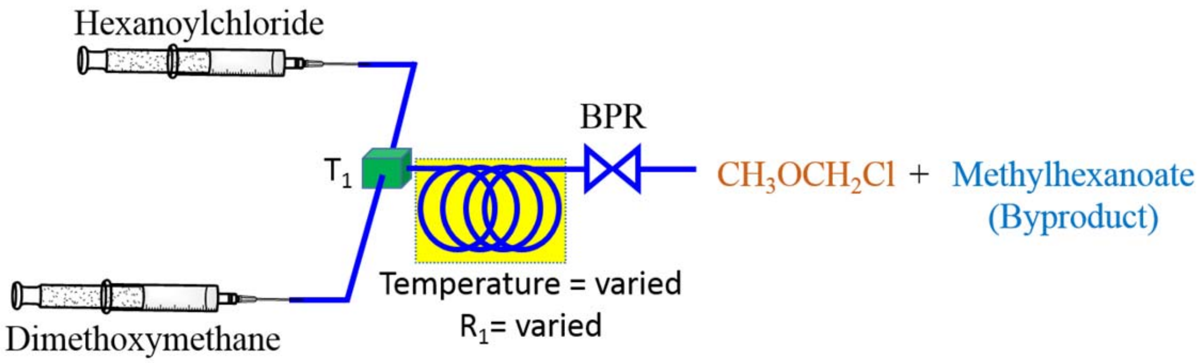

Supplementary Figure 1. Scheme for continuous-flow synthesis of CMME at neat condition with no use of solvent.

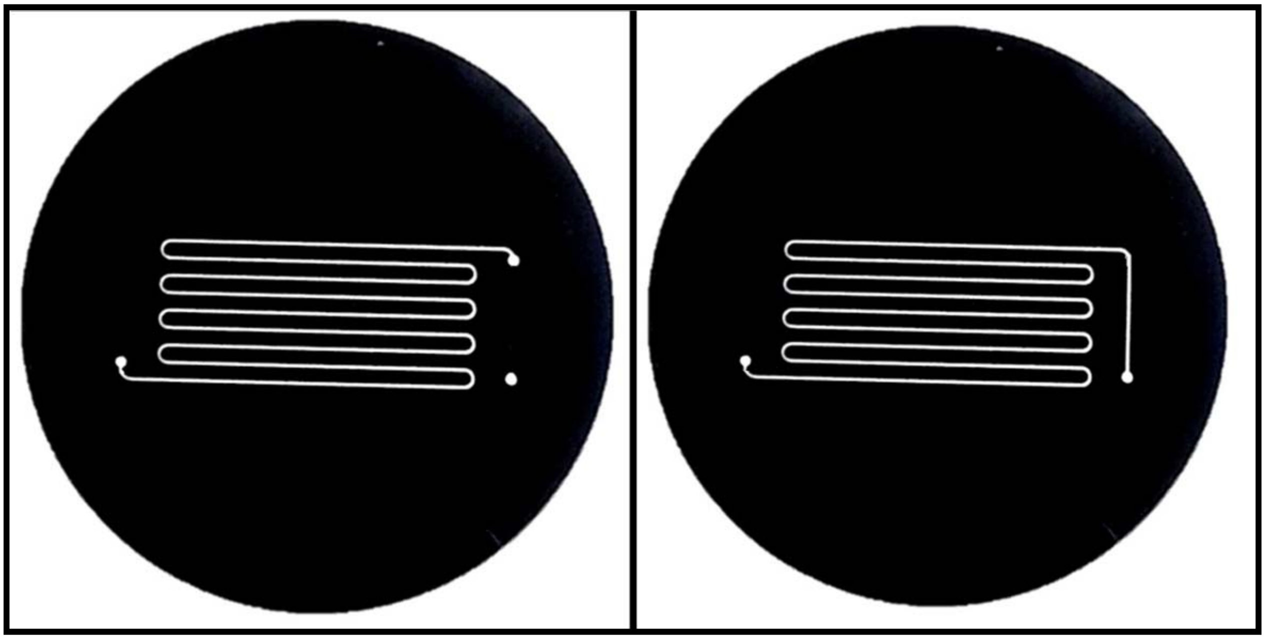

Supplementary Figure 2. Photo-masks used to make a resistant layer coated PDMS channel (left); a silicon nanowire pattern (right) in a SiNWs microseparator.

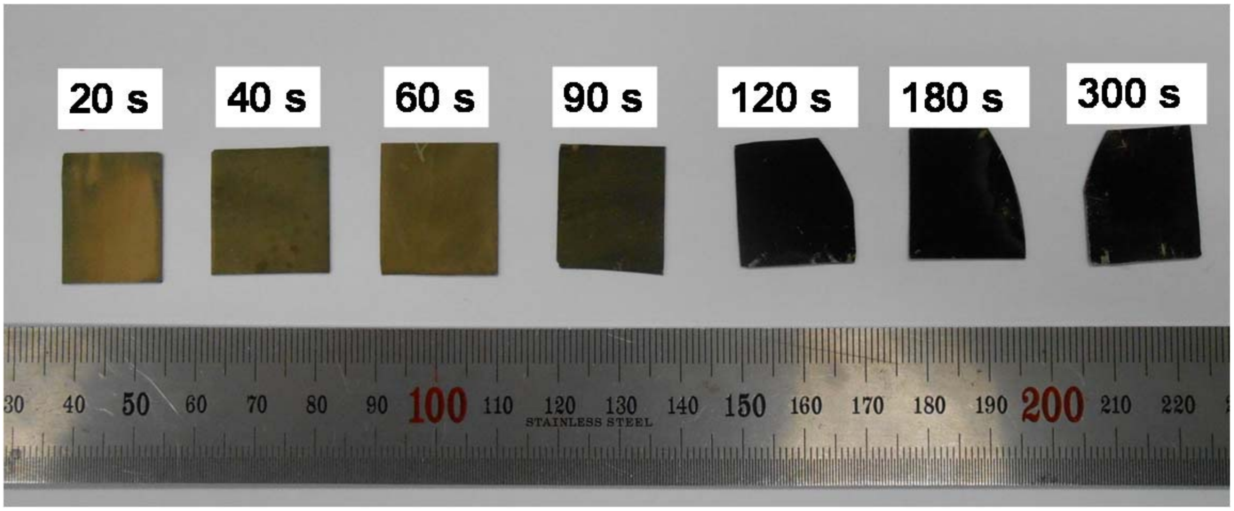

Supplementary Figure 3. Optical images of SiNWs sample with various silver loading times.

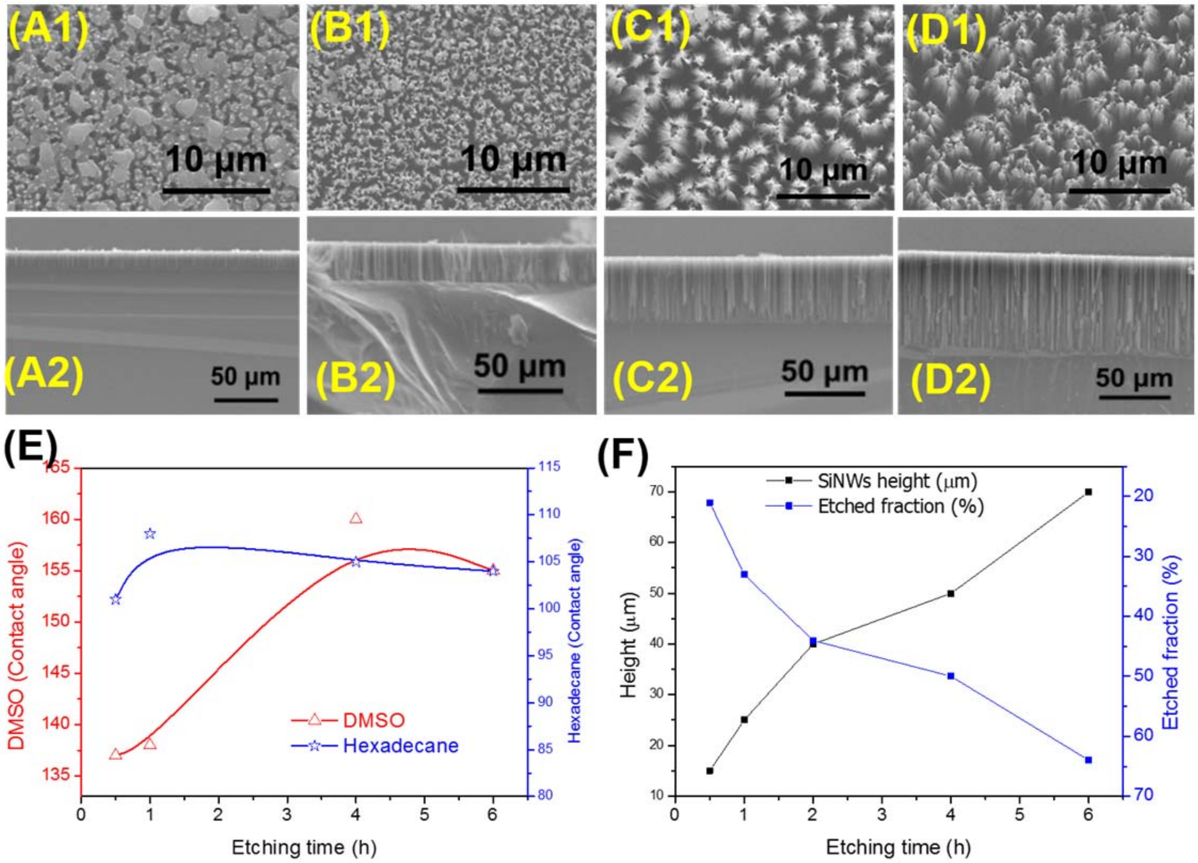

Supplementary Figure 4. Relationship of superamphiphobic SiNWs with chemical etching time from 0.5 to 5 h when silver loading time and TEOS concentration (for SiO_2 decoration) were fixed at 2 min and 24 mM, respectively; (A1-D1) Surface SEM images of 0.5, 1, 4, 6 h chemical etched SiNWs and A2-D2 corresponding to the cross-section view; (E) Contact angle of DMSO and Hexadecane; (F) SiNWs heights (μm) and Etched fraction (%) in reference with etched time.

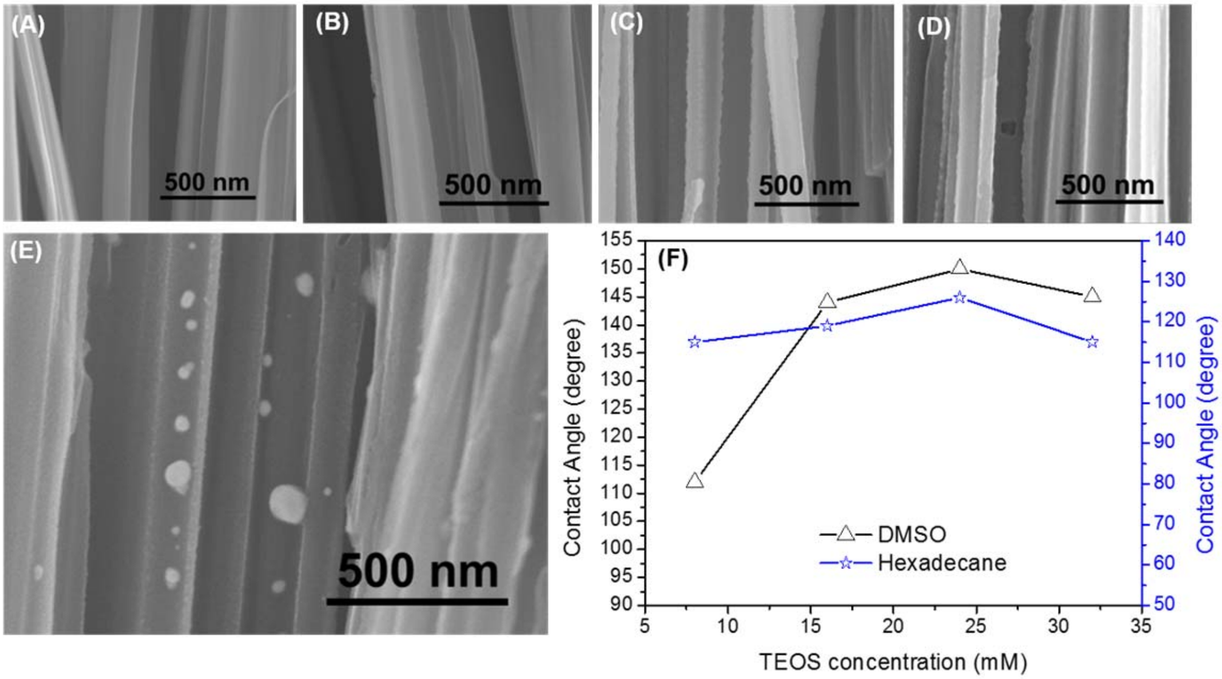

Supplementary Figure 5. Relationship of superamphiphobic SiNWs patterns with TEOS concentration for SiO_2 decoration: (A-E) SEM images of SiNWs treated with 0, 8, 16, 24, 32 mM TEOS (silver loading time and chemical etching time were fixed at 2 min and 4 h) respectively; (F) Contact angle of DMSO and Hexadecane.

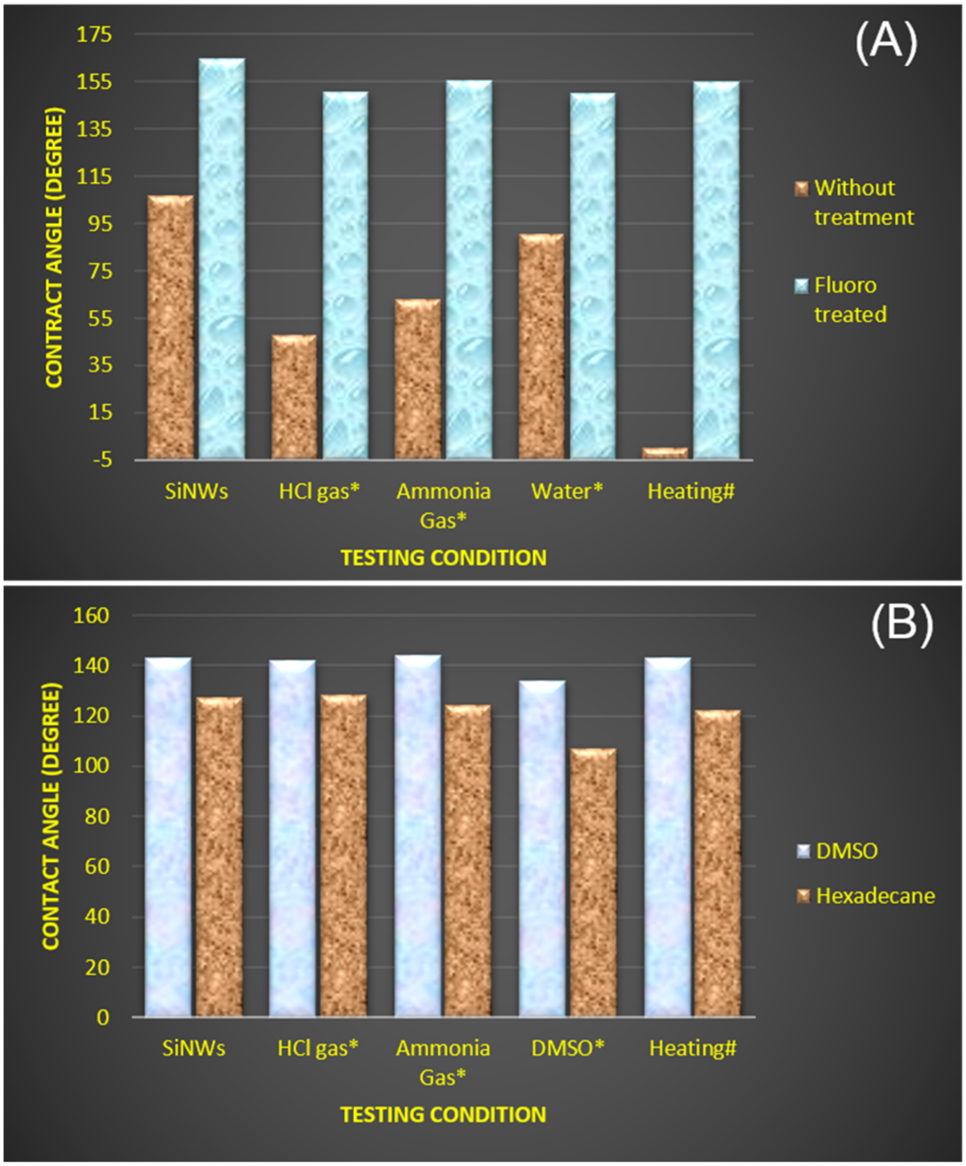

Supplementary Figure 6. Chemical and thermal stability of superamphiphobic SiNWs: (A) Water CA of SiNWs without and with surface fluorination treatment; (B) DMSO and hexadecane CA on fluorinated SiNWs. (* = 24 h gas exposed, # = 300 ^{o}C heated SiNW for 1h in air).

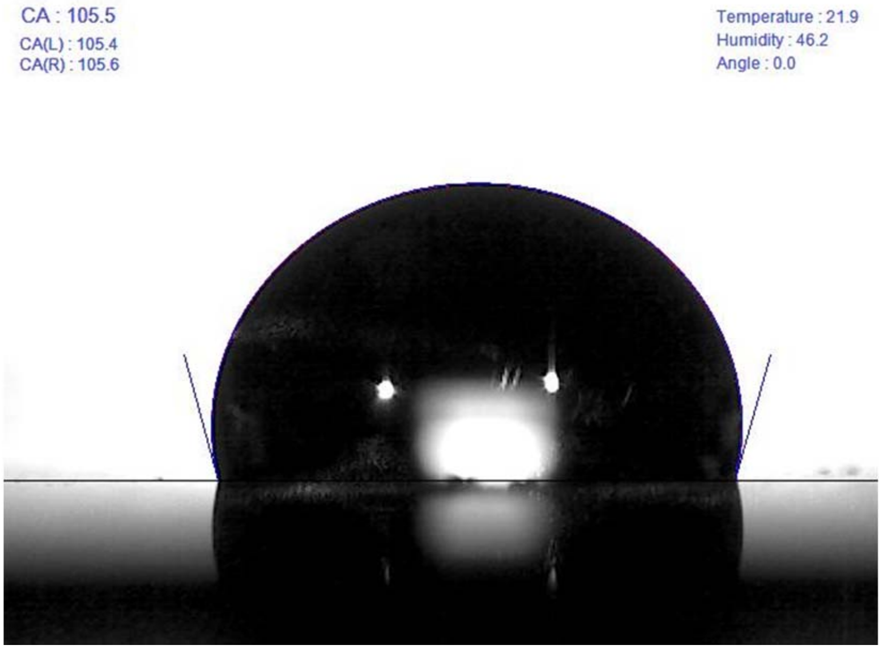

Supplementary Figure 7. Methyl hexanoate contact angle on Fluorotreated SiNWs surface.

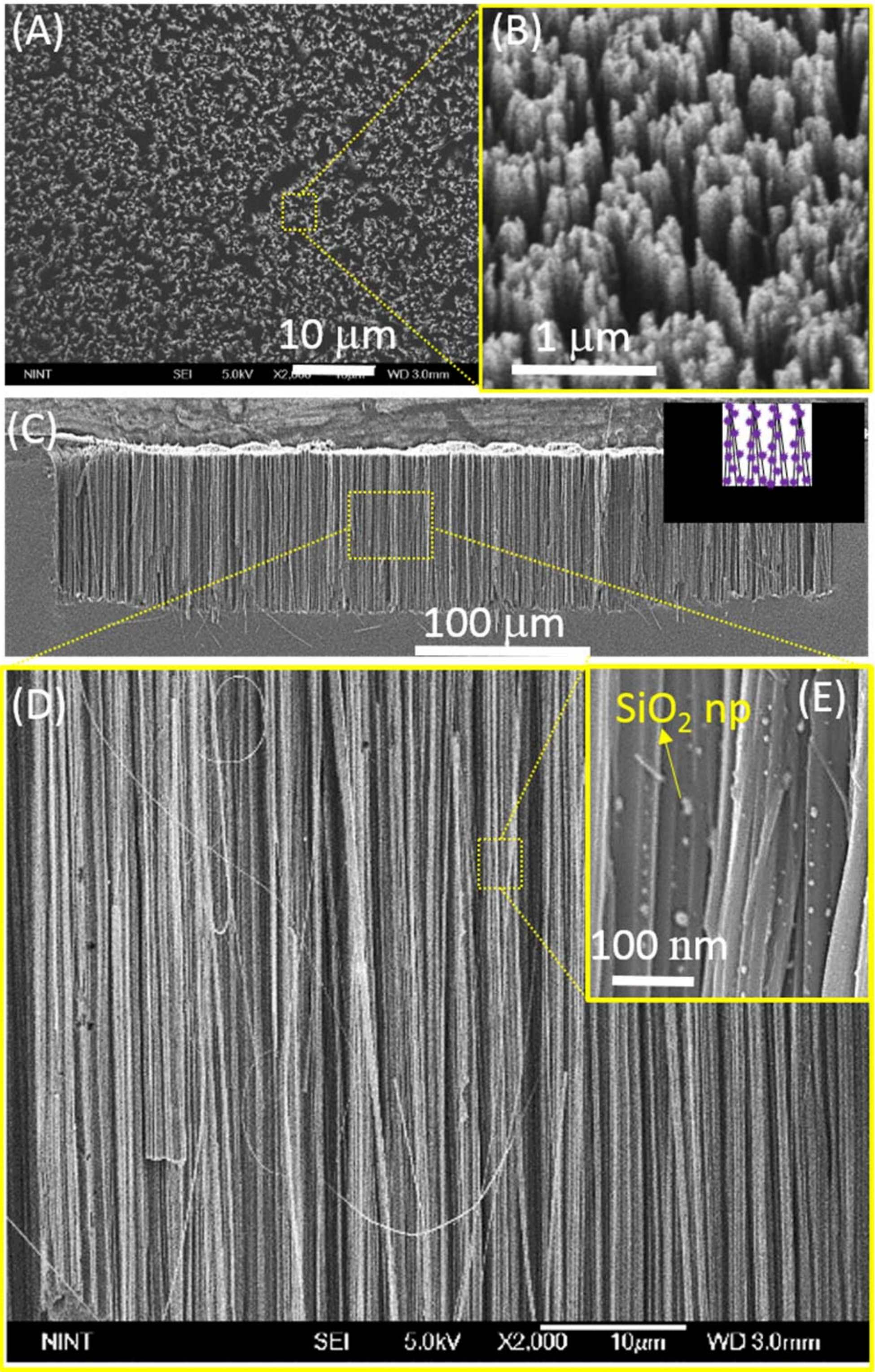

Supplementary Figure 8. (A & B) Top-view SEM images of fluorosilicate coated SiNWs at low and high resolution; (C-E) Cross-sectional SEM image of vertically aligned fluorosilicate coated SiNWs pattern at low and high resolution respectively.

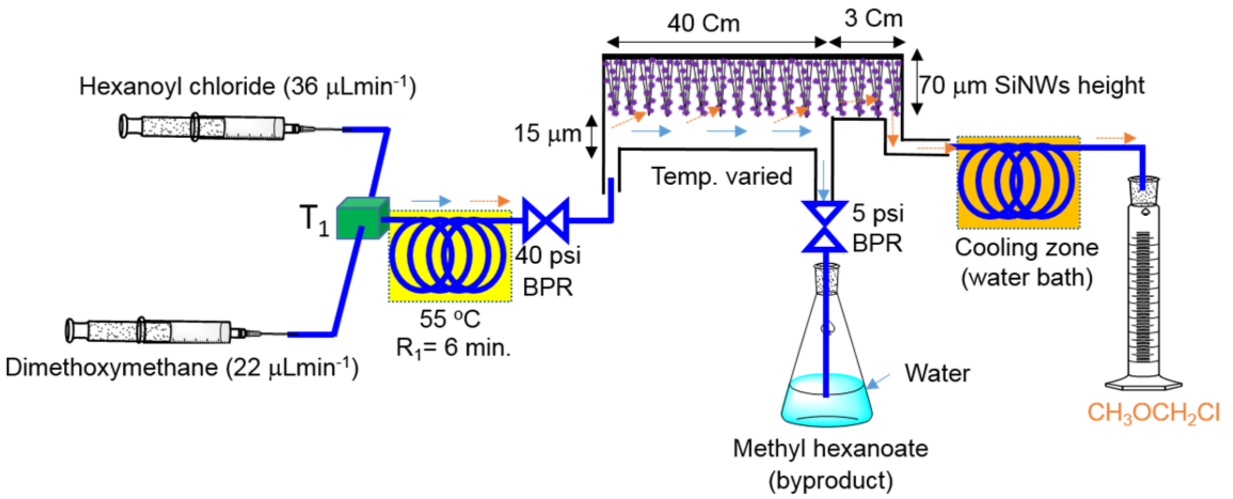

Supplementary Figure 9. Scheme of in situ distilled separation of volatile CMME in a serial and continuous-flow manner by newly designed membrane-free SiNWs microseparator.

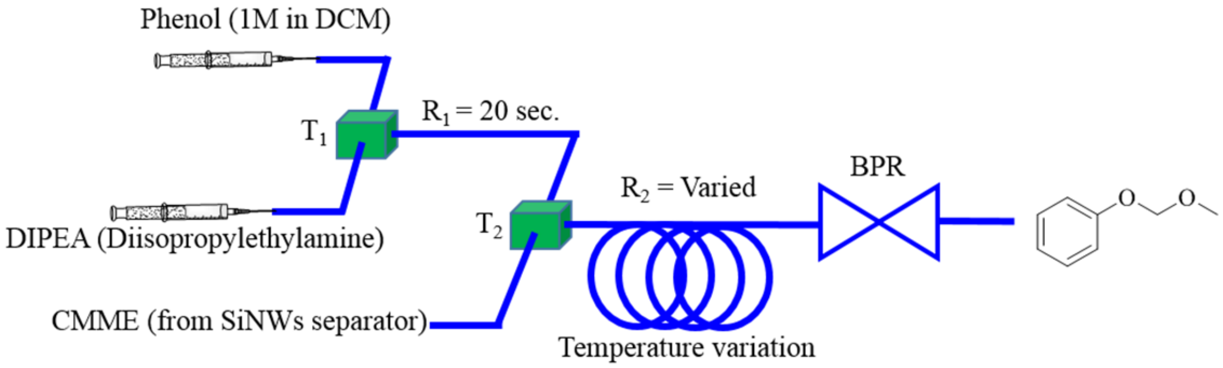

Supplementary Figure 10. Schematic presentation of phenol group protection by a serial process of CMME generation, separation, in situ consumption.

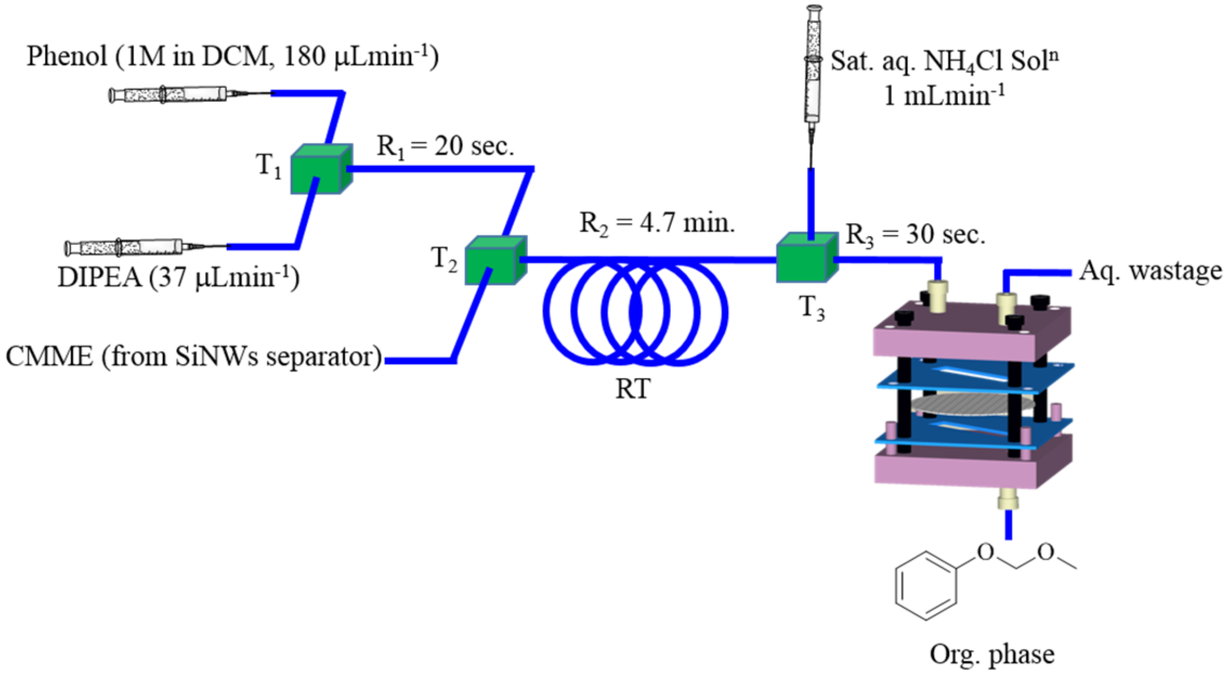

Supplementary Figure 11. Fully automated integrated system for a total process of phenol group protection by serial CMME generation, separation, in situ consumption and quenching step.

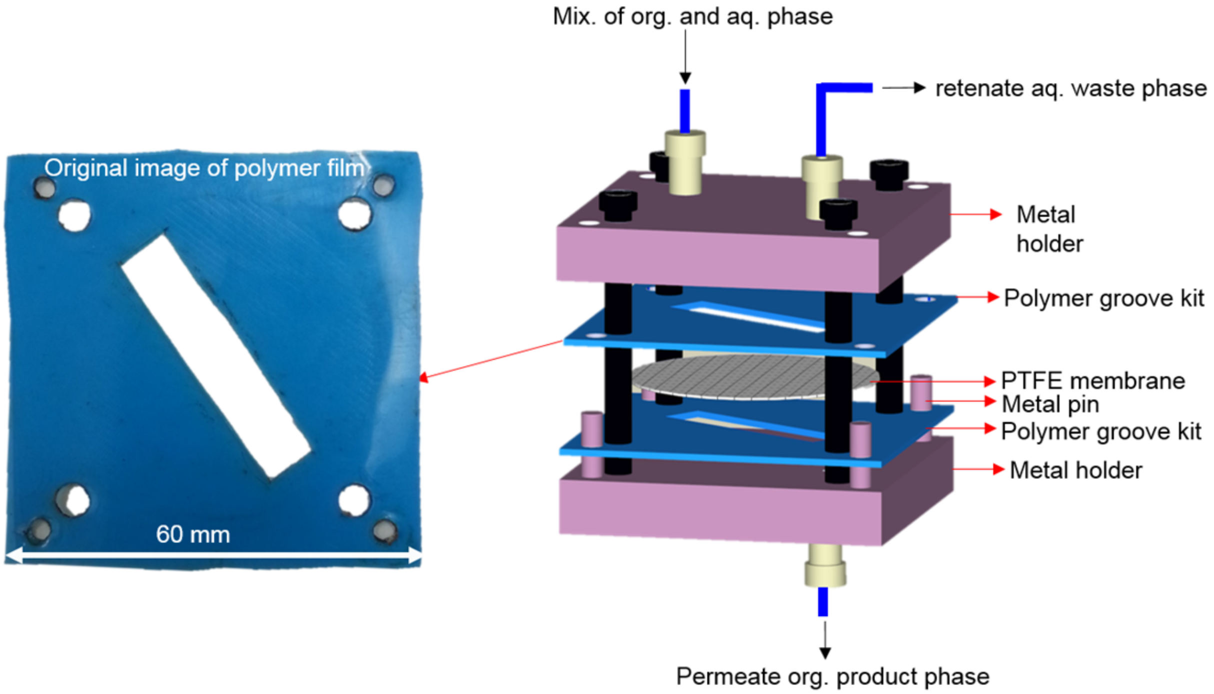

Supplementary Figure 12. Illustration of a fluoropolymer PTFE membrane microseparation sandwiched between two polyethylene films with punched channel.

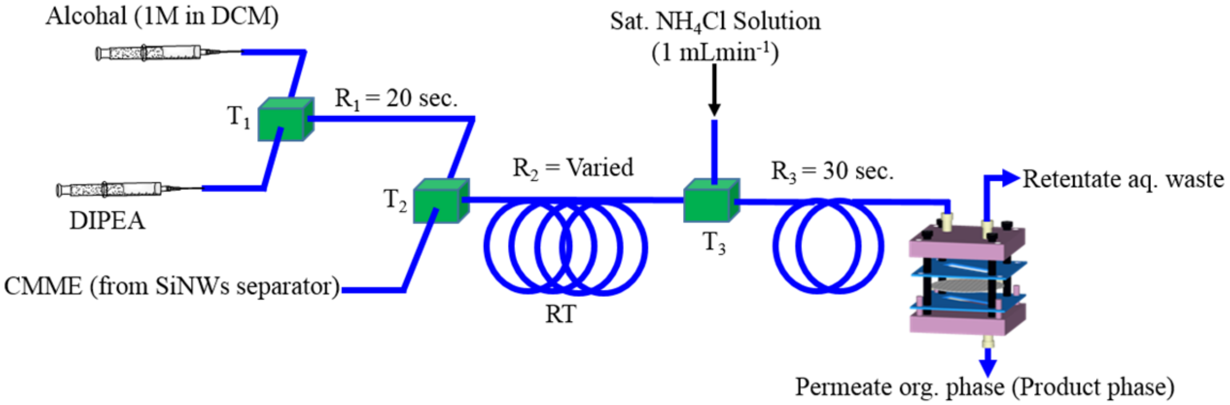

Supplementary Figure 13. Integrated microfluidic process for alcohal group protection by an automated serial process of CMME generation, separation, in situ consumption and quenching step.

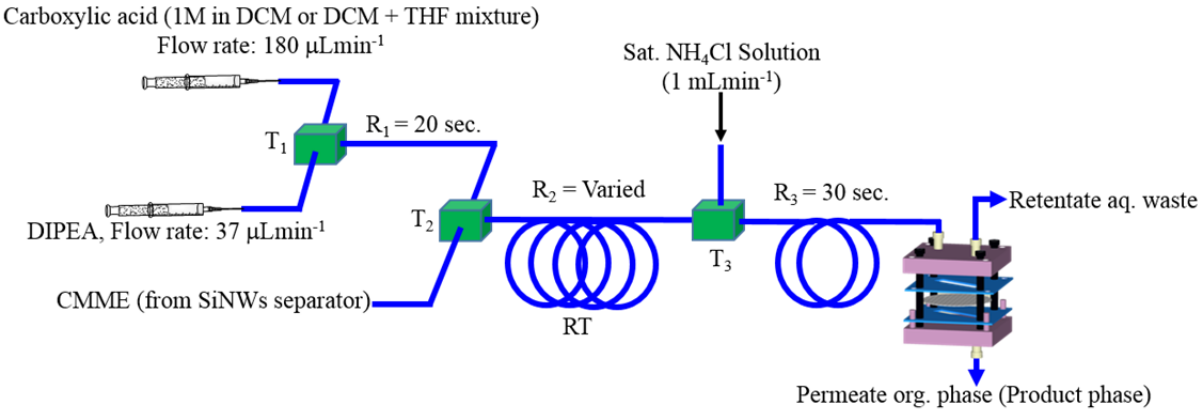

Supplementary Figure 14. Integrated microfluidic process for carboxylic acid group protection by an automated serial process of CMME generation, separation, in situ consumption and quenching step.

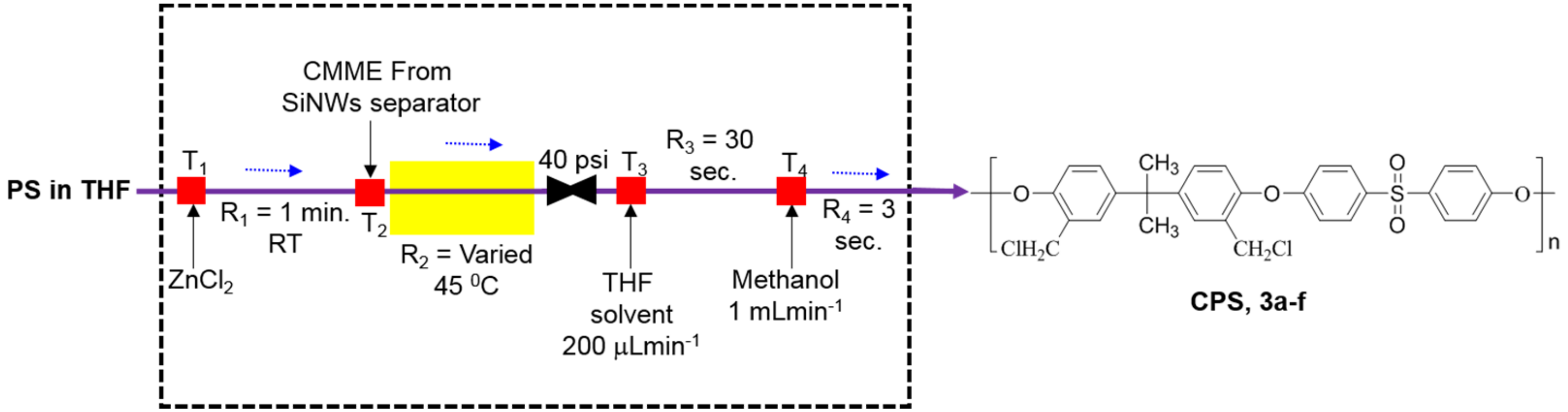

Entry	PS solution flow rate (μLmin^{-1})	$ZnCl_2$ Flow rate (μLmin^{-1})	R_2 capillary length (m)	Retention time R_2 (min)	DCM[(a)]
1	160	14	6	6.2	0.07
2	160	14	8.8	9	0.49
3	160	14	13.5	14	0.8
4	160	14	14.5	15	1.0
5	160	14	17.4	18	1.41
6	160	14	21.3	22	1.9

[(a)]degree of chloromethylation is based on NMR analysis.

Supplementary Figure 15. Scheme and performance of Friedel-Craft chloromethylation of polysulphone by the integrated microfluidic system in an automated serial process of CMME generation, separation, in situ consumption and quenching step.

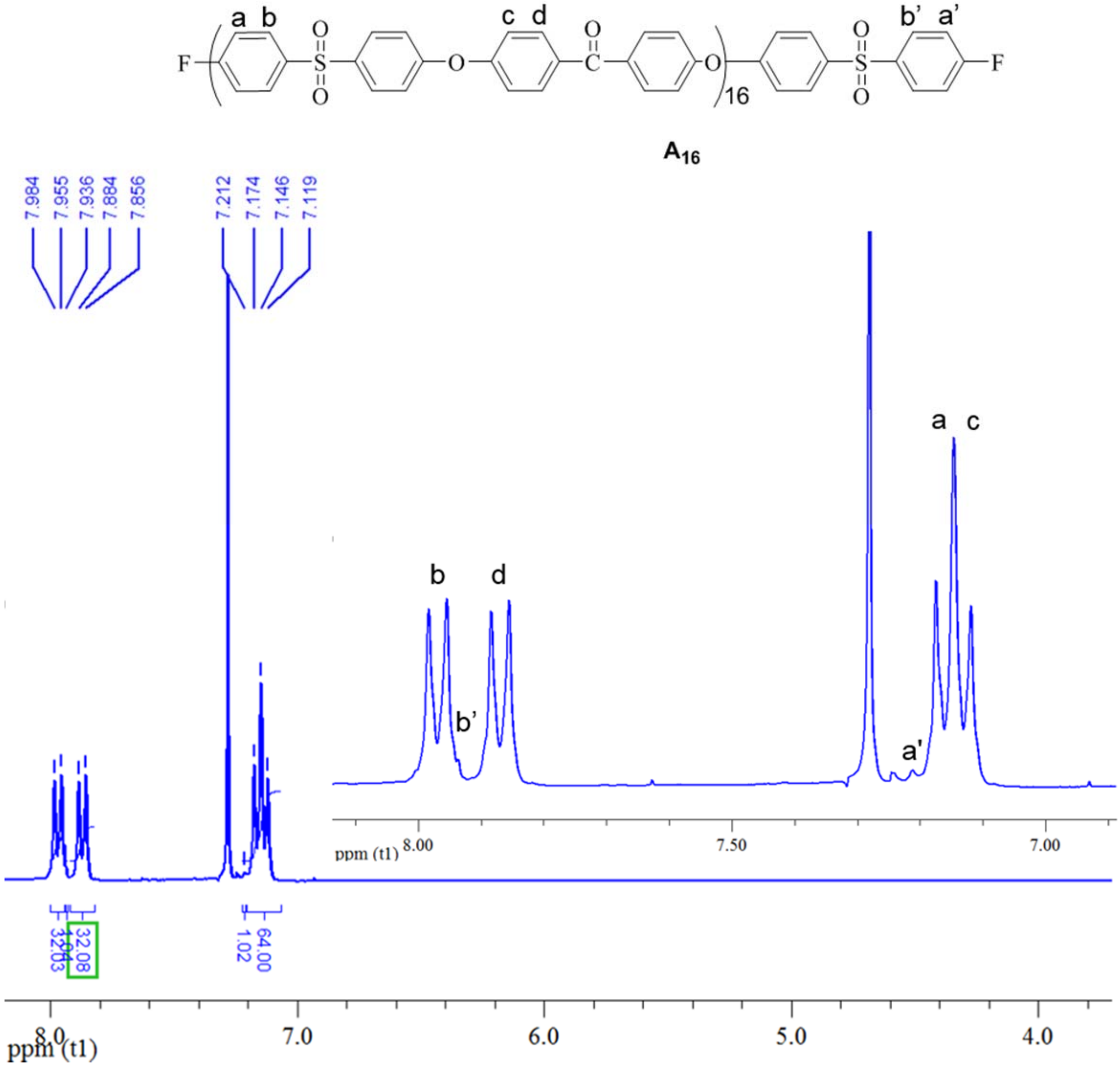

Supplementary Figure 16. ^{1}H NMR spectra of hydrophobic fluorine-terminated oligomer A_{16}.

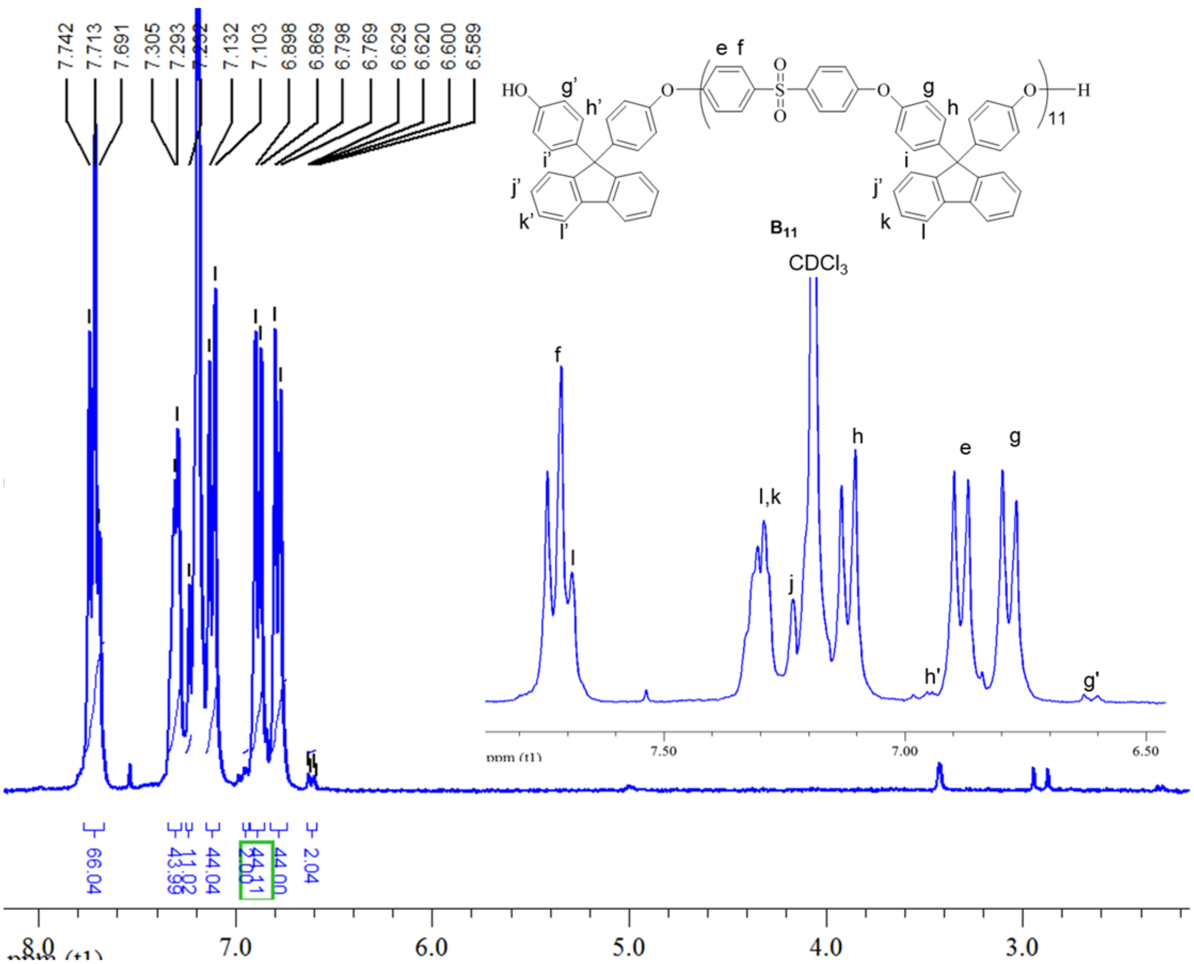

Supplementary Figure 17. ^{1}H NMR spectra of hydrophilic hydroxy-terminated oligomer B_{11}.

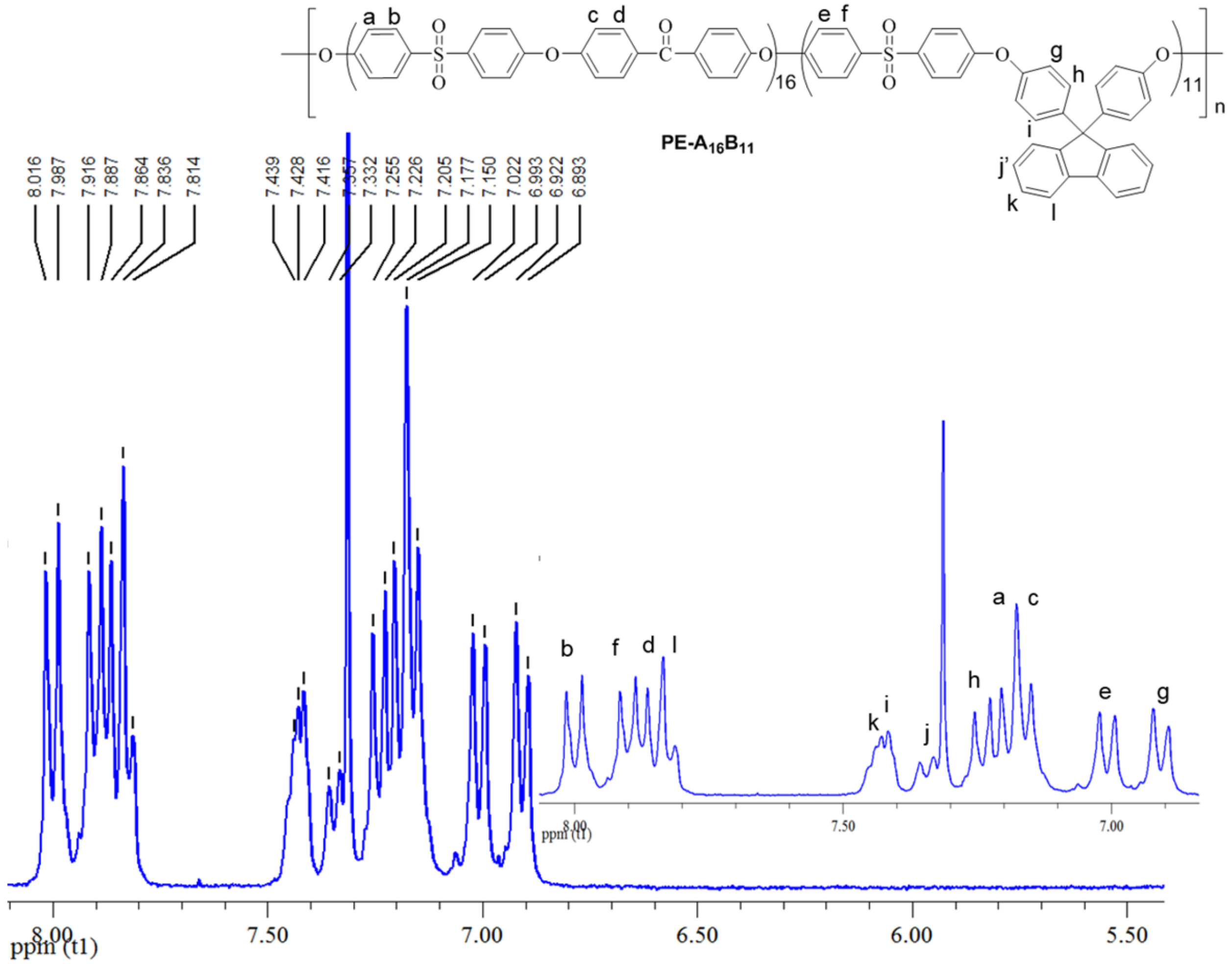

Supplementary Figure 18. ^{1}H NMR spectra of poly(arylene ether) PE-$A_{16}B_{11}$ multiblock copolymer.

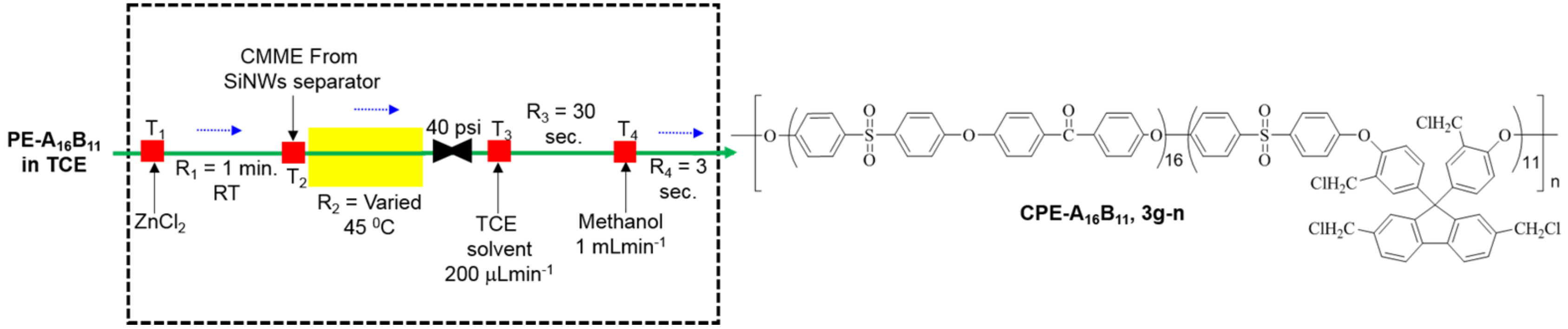

Entry	PE-$A_{16}B_{11}$ solution flow rate ($\mu Lmin^{-1}$)	$ZnCl_2$ solution flow rate ($\mu Lmin^{-1}$)	R_2 capillary length (m)	Retention time R_2 (min)	DCM[(b)]
1	101	11	0.7	1.0	0.09
2	101	11	2.0	3.0	0.19
3	101	11	3.3	5.0	0.39
4	101	11	9.8	15.0	1.58
5	101	11	16.3	25.0	1.8
6	101	11	22.8	35.0	2.18
7	101	11	29.4	45.0	2.44
8	101	11	39.1	60.0	2.94

[(b)]degree of chloromethylation is based on NMR analysis.

Supplementary Figure 19. Scheme and performance of a total process for Friedel-Craft chloromethylation of PE-$A_{16}B_{11}$ polymer by integrated microfluidic system in an automated serial CMME generation, separation, in situ consumption and quenching step.

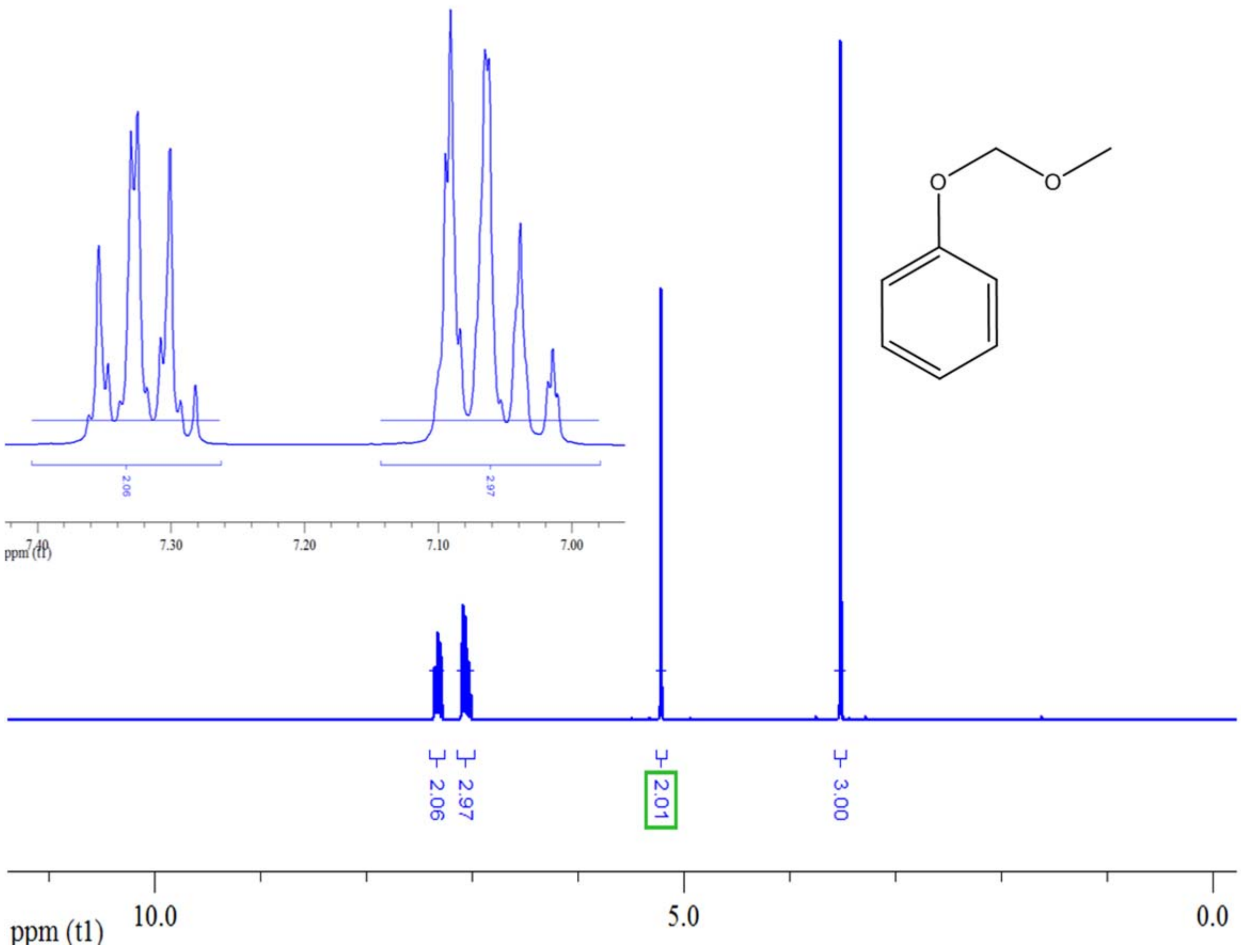

Supplementary Figure 20. ^{1}H NMR Spectra of (Methoxymethoxy)benzene (**2a**)

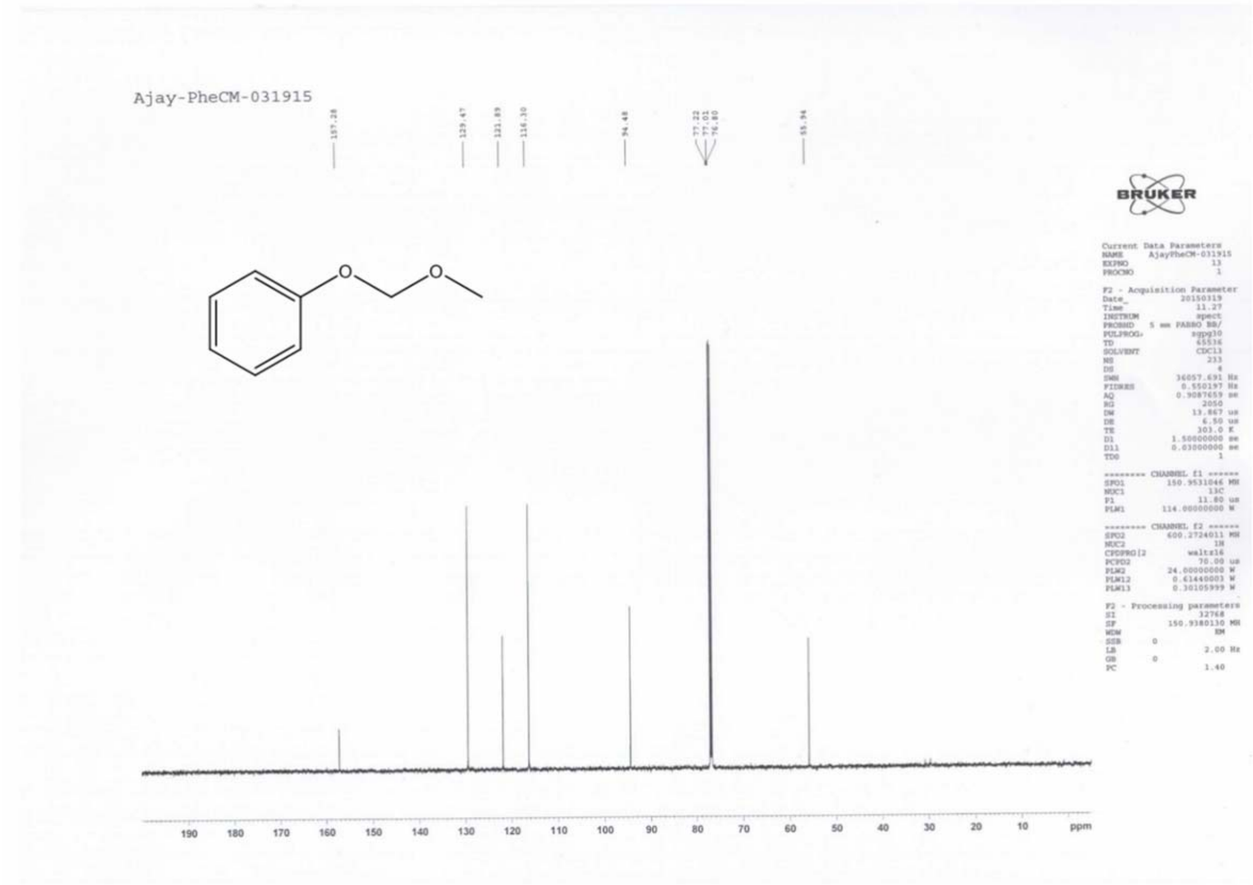

Supplementary Figure 21. ^{13}C NMR Spectra of (Methoxymethoxy)benzene (**2a**)

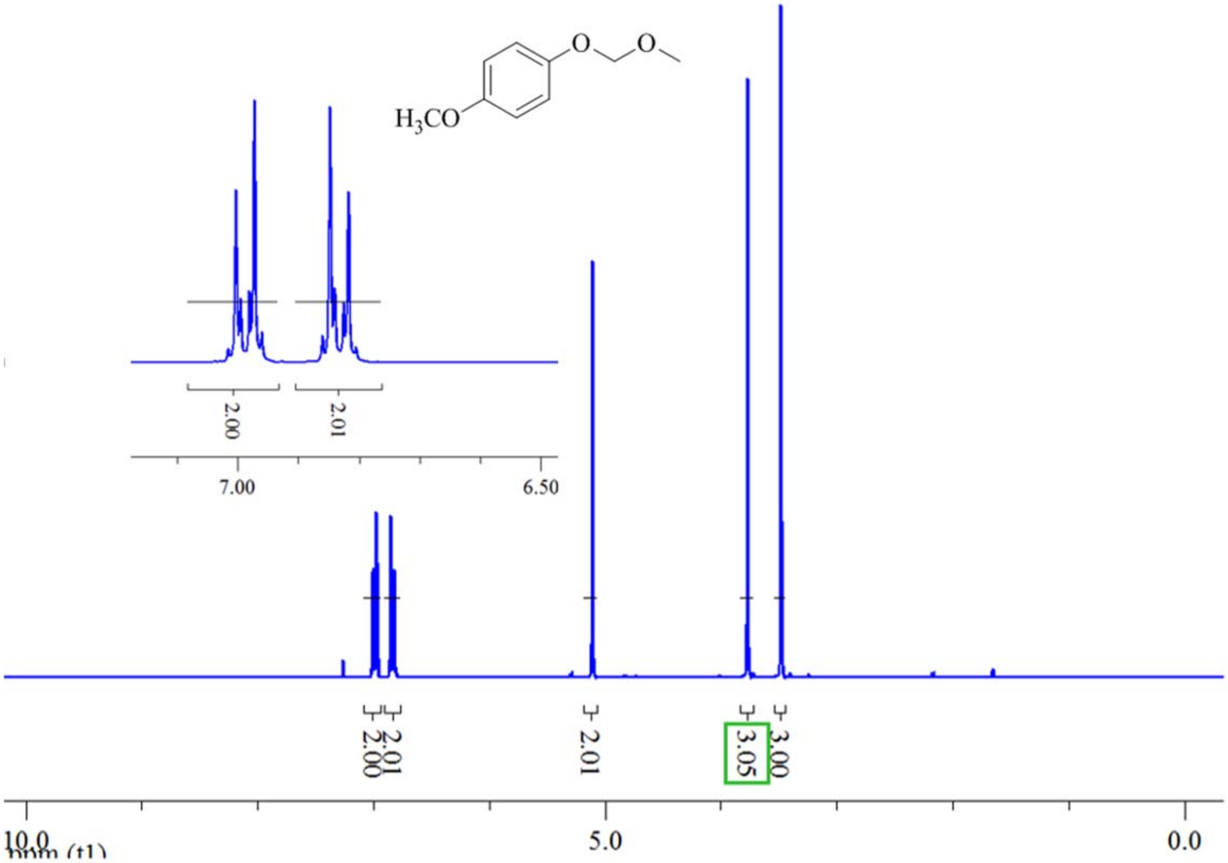

Supplementary Figure 22. ^{1}H NMR Spectra of 1-Methoxy-4-(methoxymethoxy)benzene (**2b**).

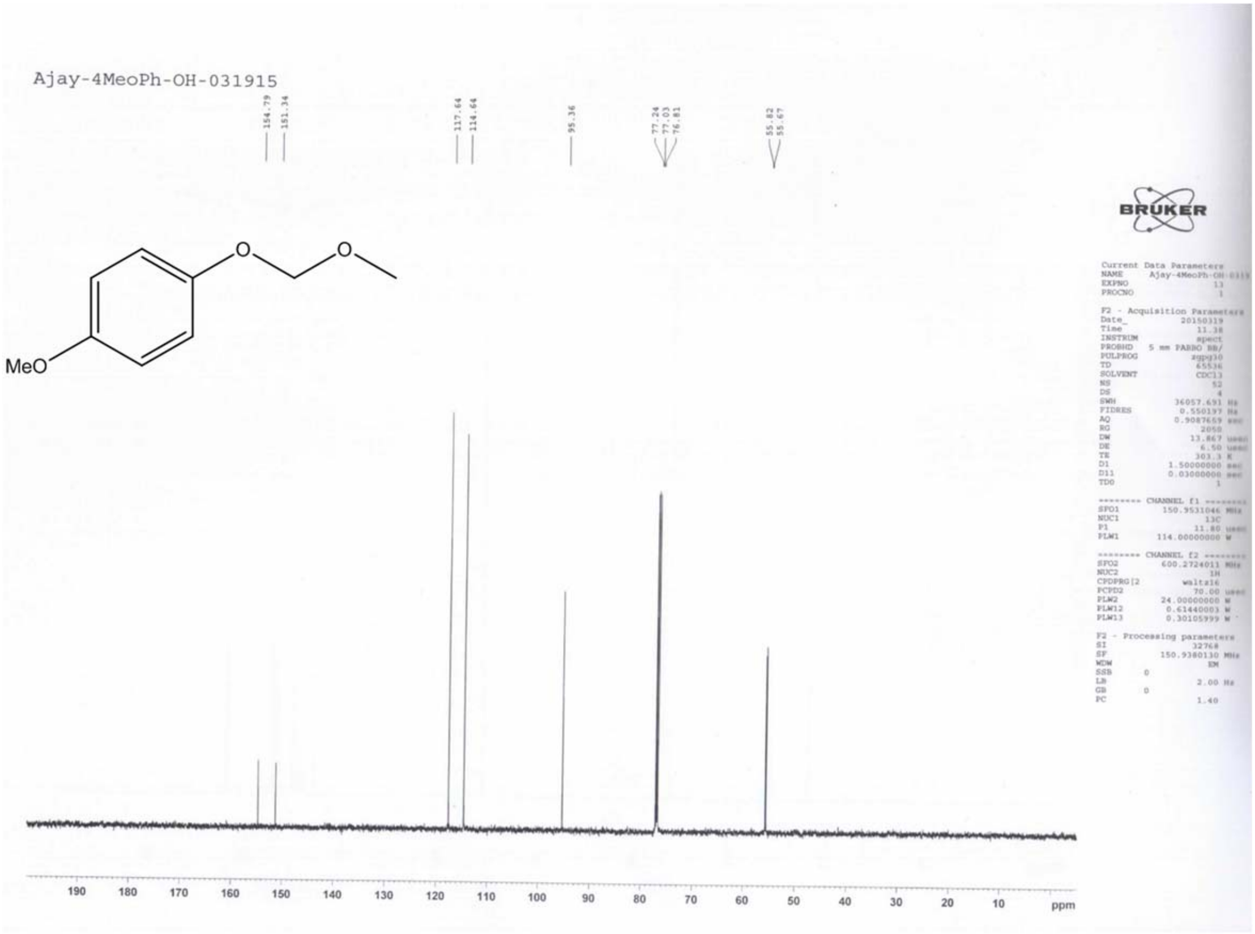

Supplementary Figure 23. ^{13}C NMR Spectra of 1-Methoxy-4-(methoxymethoxy)benzene (**2b**).

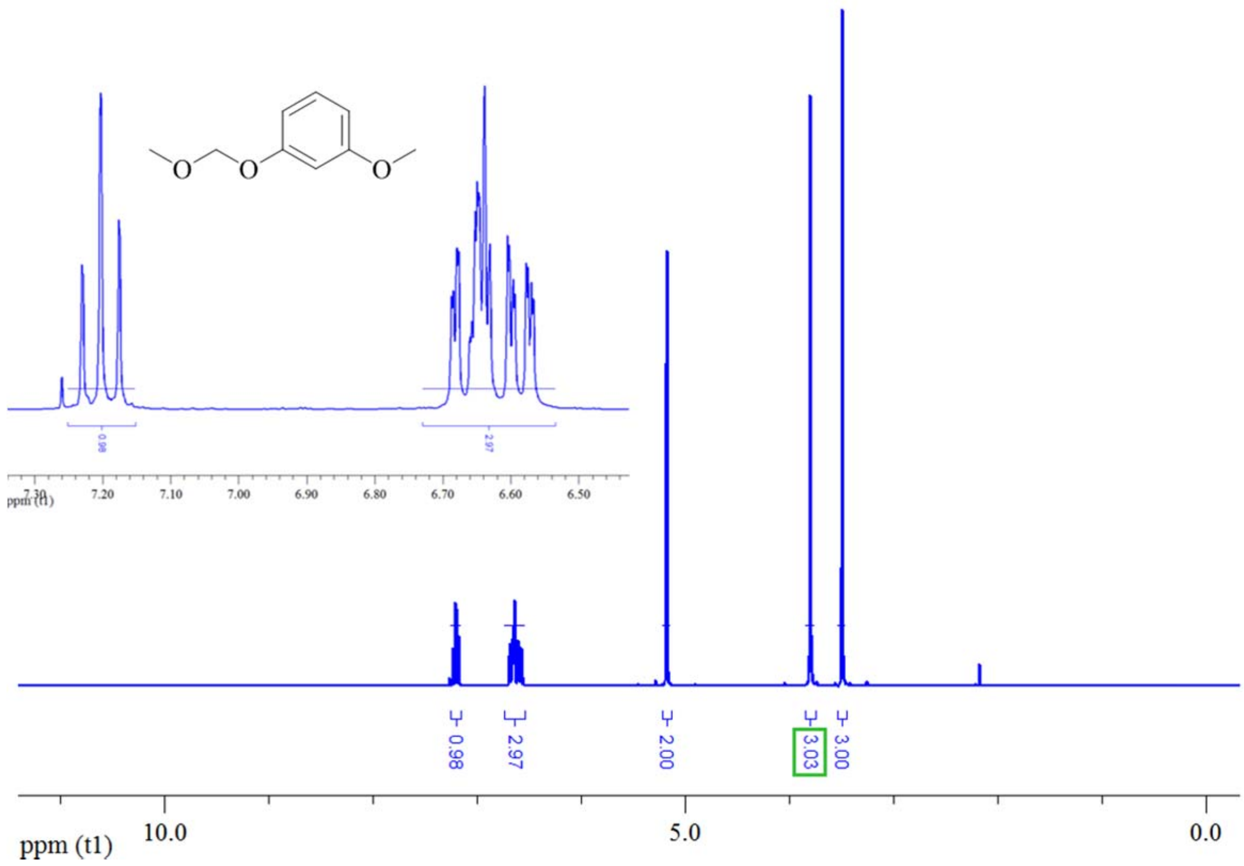

Supplementary Figure 24. ^{1}H NMR Spectra of 1-Methoxy-3-(methoxymethoxy)benzene (**2c**).

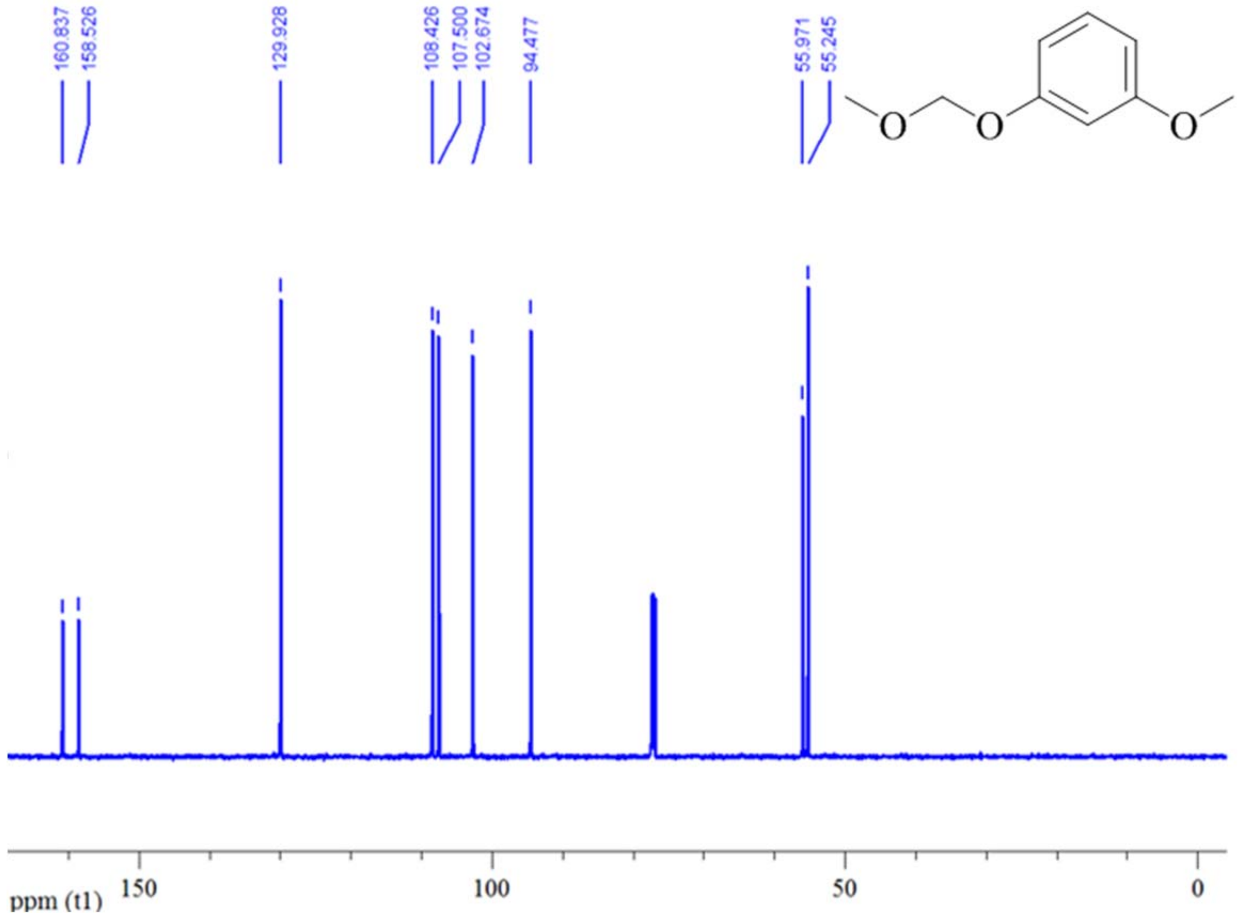

Supplementary Figure 25. ^{12}C NMR Spectra of 1-Methoxy-3-(methoxymethoxy)benzene (**2c**).

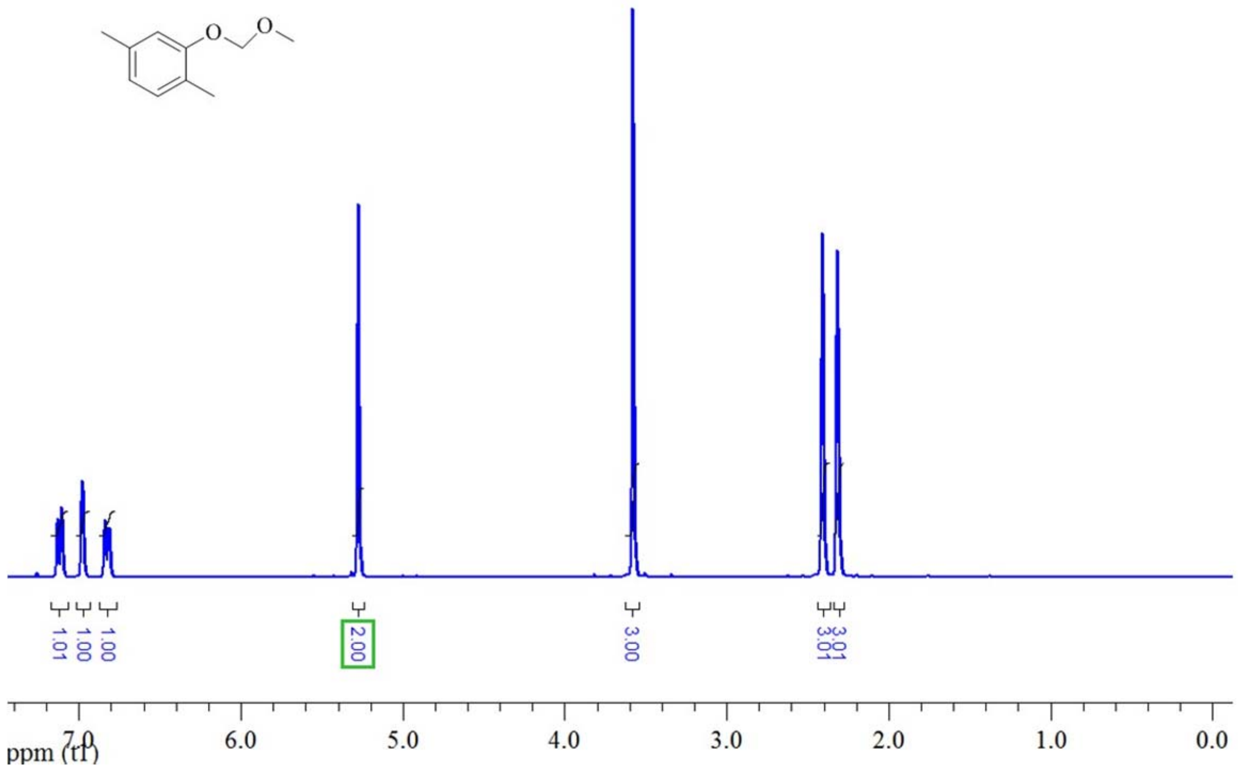

Supplementary Figure 26. ^{1}H NMR Spectra of 2-(methoxymethoxy)-1,4-dimethylbenzene (**2d**).

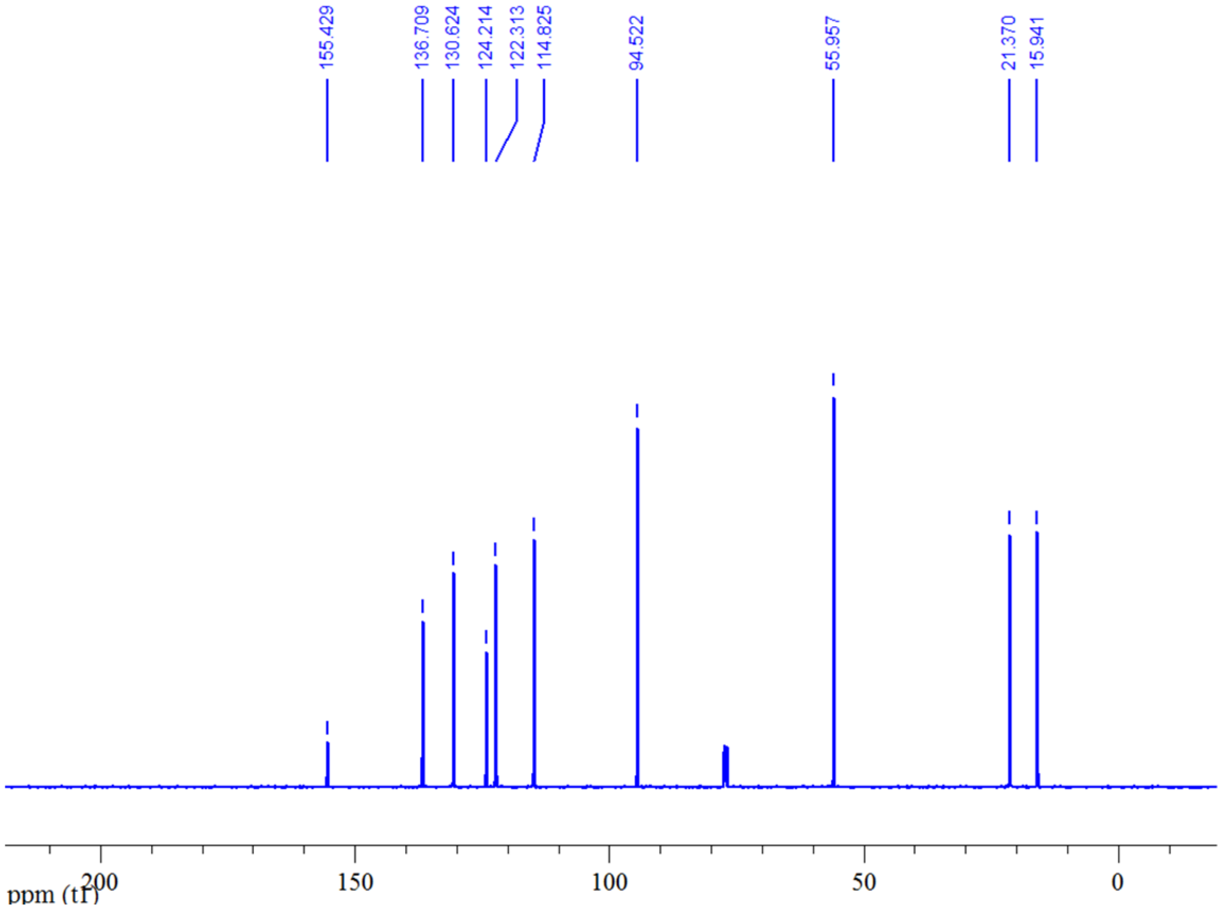

Supplementary Figure 27. ^{13}C NMR Spectra of 2-(methoxymethoxy)-1,4-dimethylbenzene (**2d**).

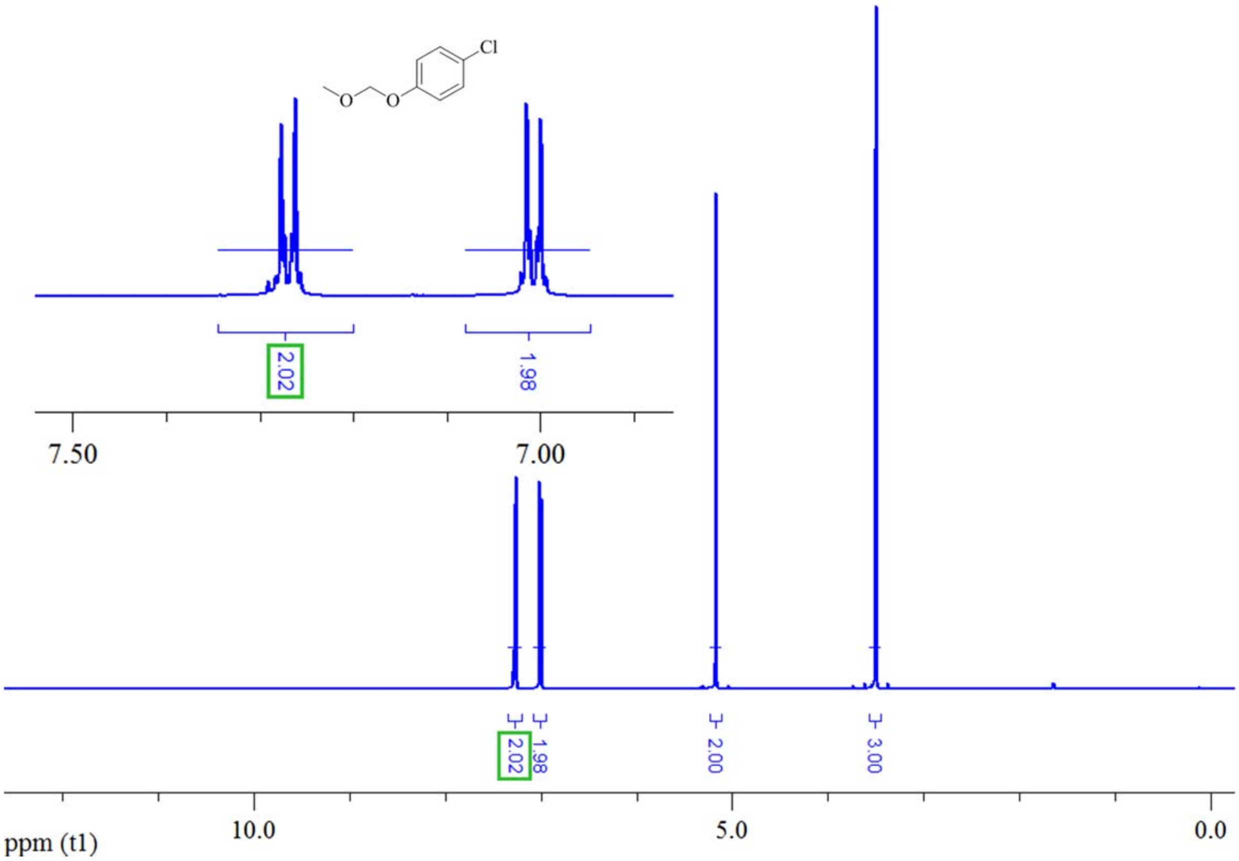

Supplementary Figure 28. ^{1}H NMR Spectra of 1-chloro-4-(methoxymethoxy)benzene (**2e**).

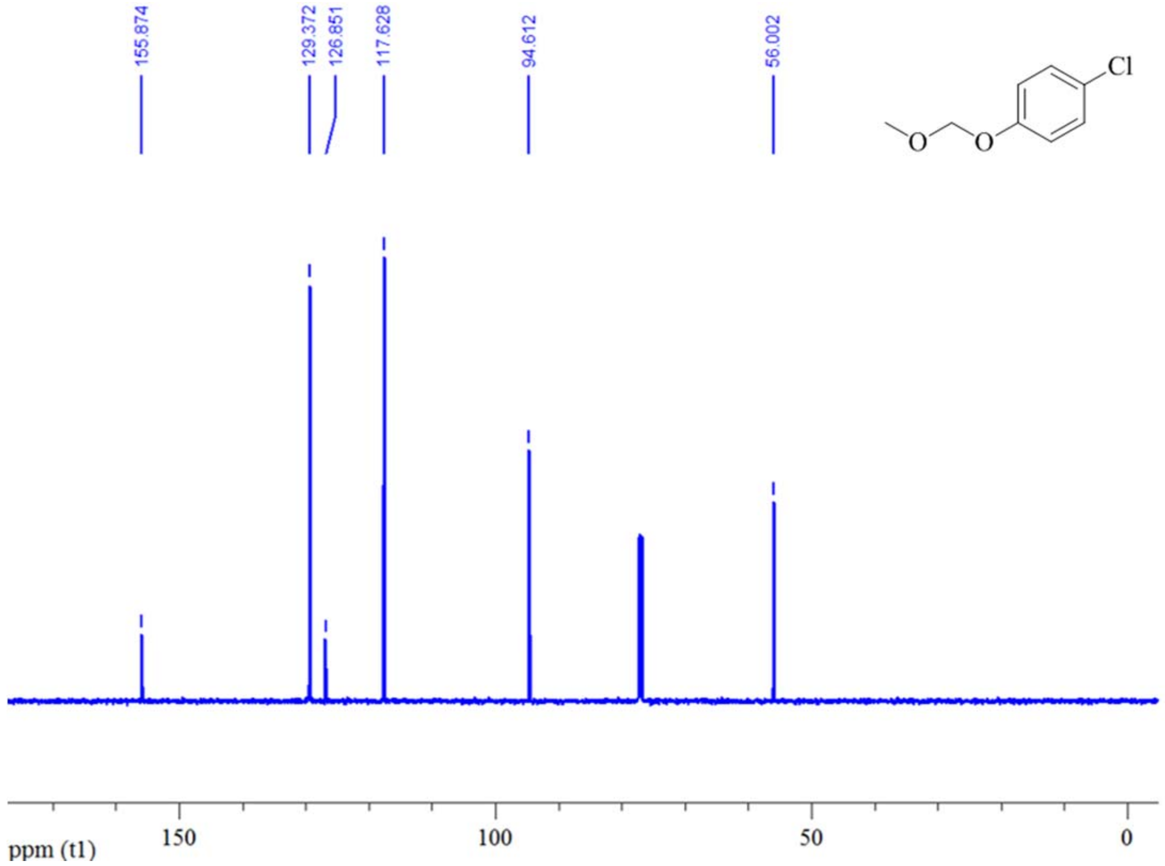

Supplementary Figure 29. ^{13}C NMR Spectra of 1-chloro-4-(methoxymethoxy)benzene (**2e**).

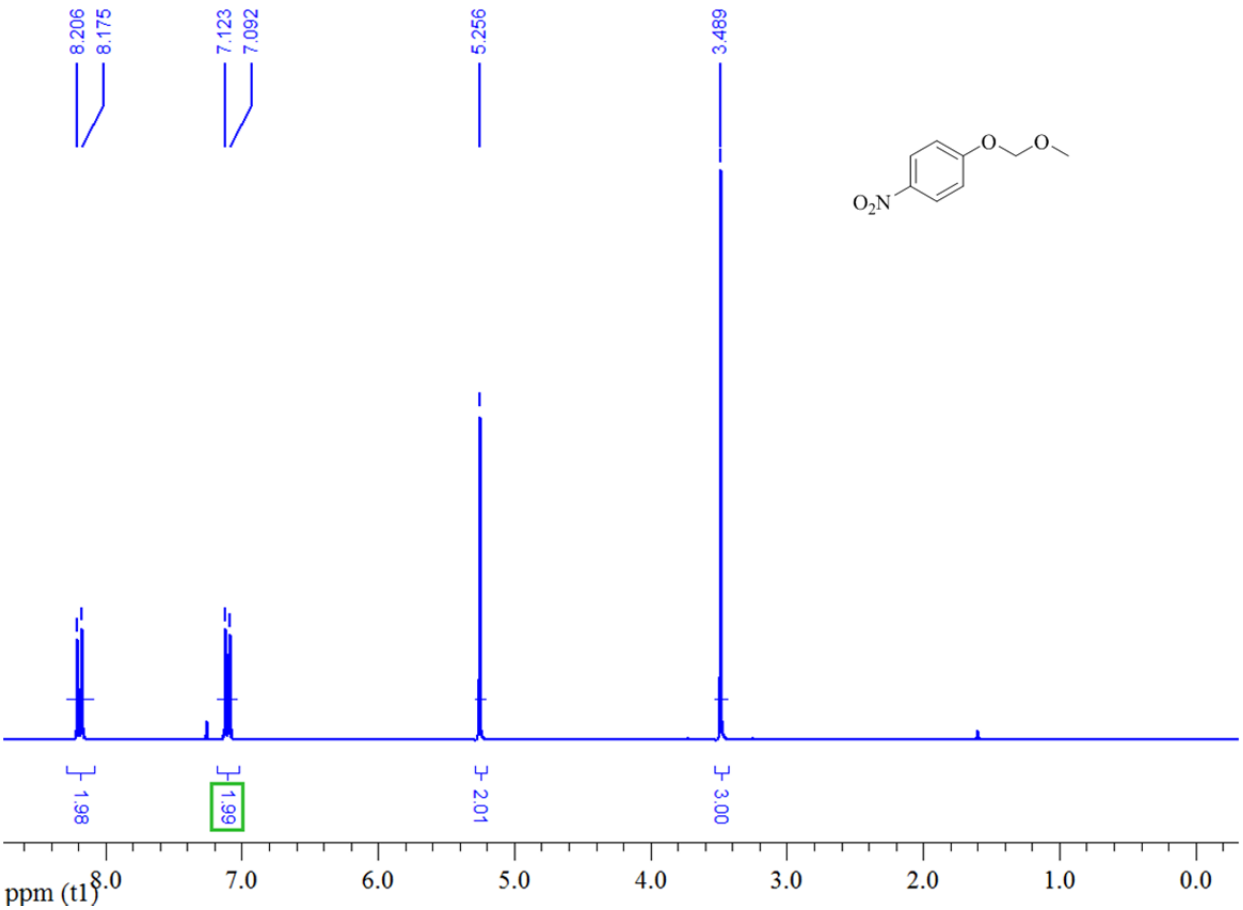

Supplementary Figure 30. ^{1}H NMR Spectra of 1-(Methoxymethoxy)-4-nitrobenzene (**2f**).

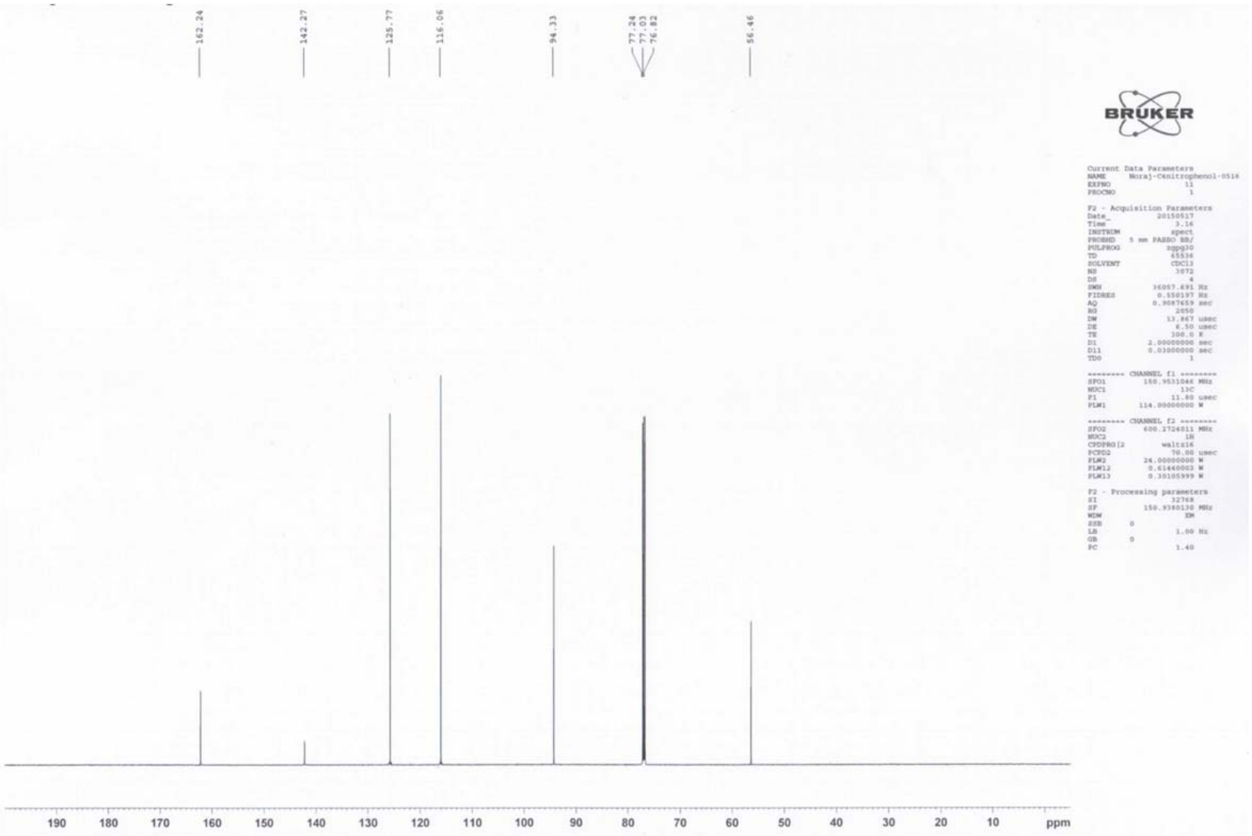

Supplementary Figure 31. ^{13}C NMR Spectra of 1-(Methoxymethoxy)-4-nitrobenzene (**2f**).

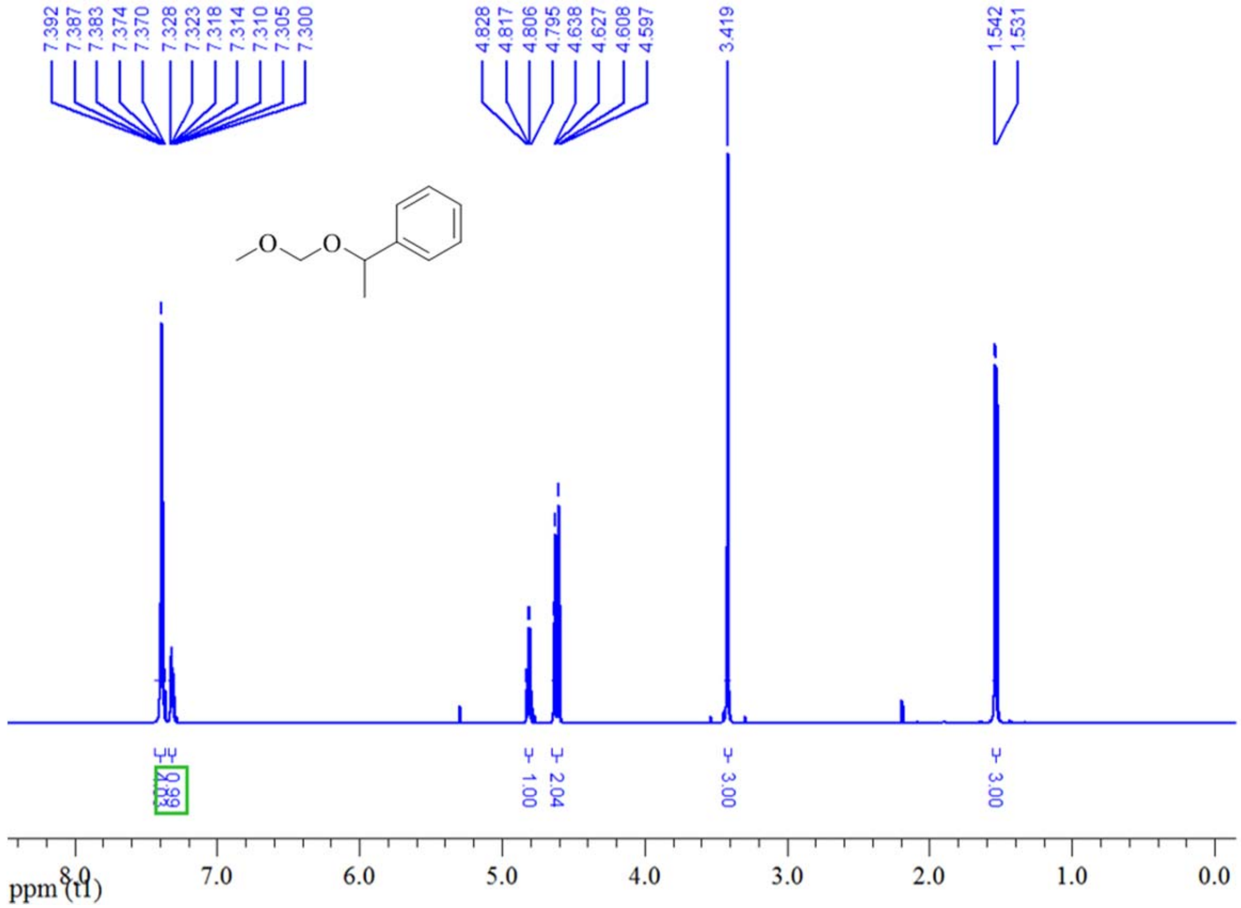

Supplementary Figure 32. ^{1}H NMR Spectra of (1-(Methoxymethoxy)ethyl)benzene (**2g**).

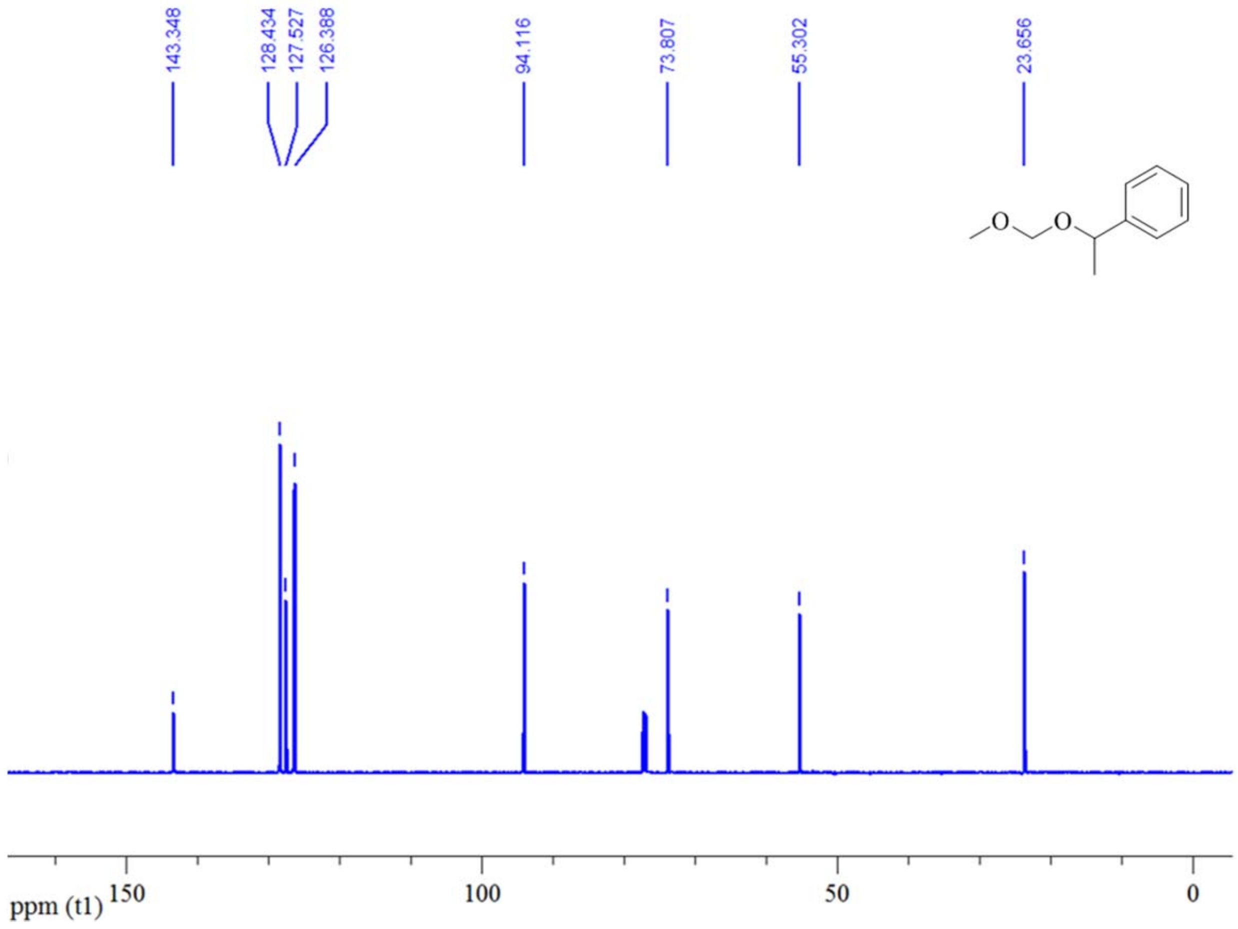

Supplementary Figure 33. ^{13}C NMR Spectra of (1-(Methoxymethoxy)ethyl)benzene (**2g**).

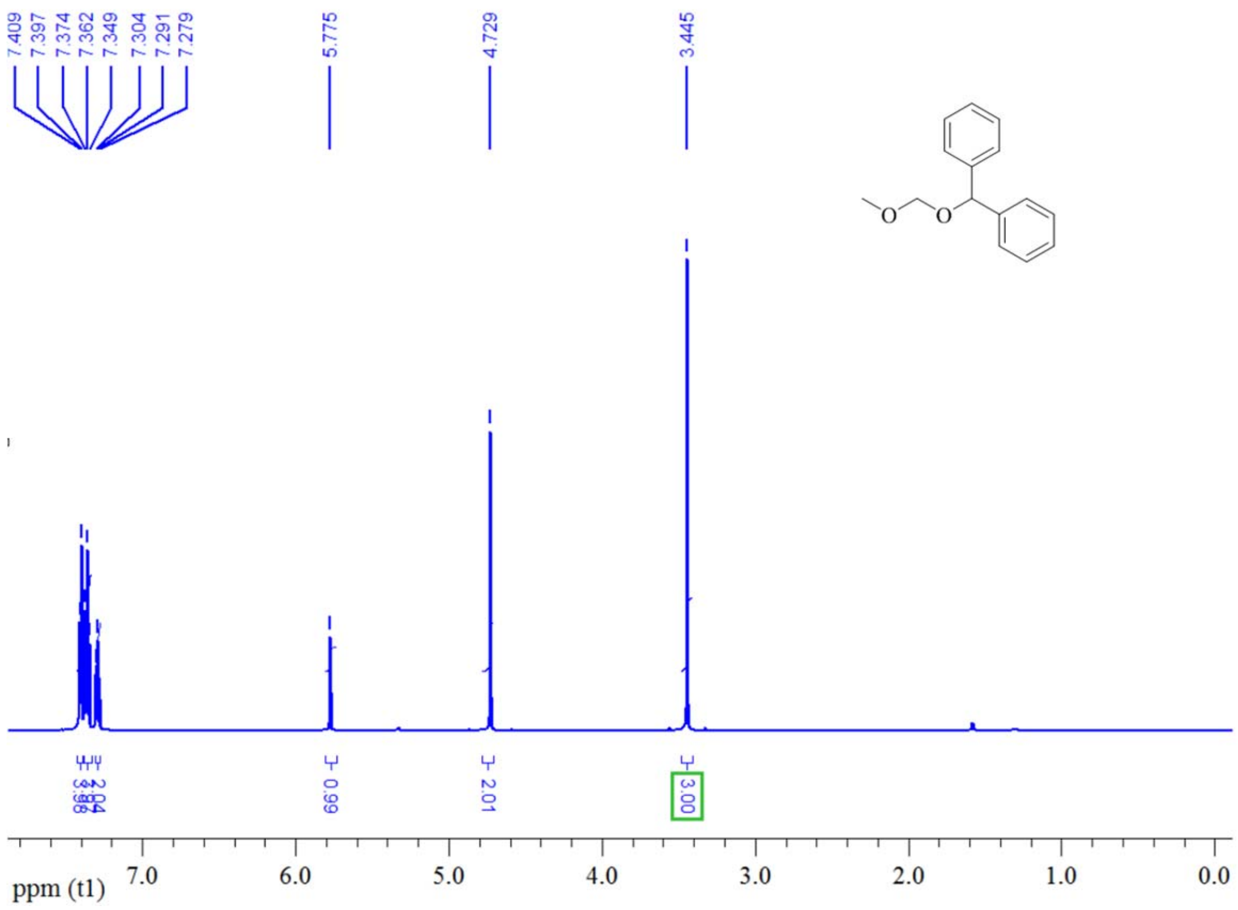

Supplementary Figure 34. ^{1}H NMR Spectra of ((Methoxymethoxy)methylene)dibenzene (**2h**).

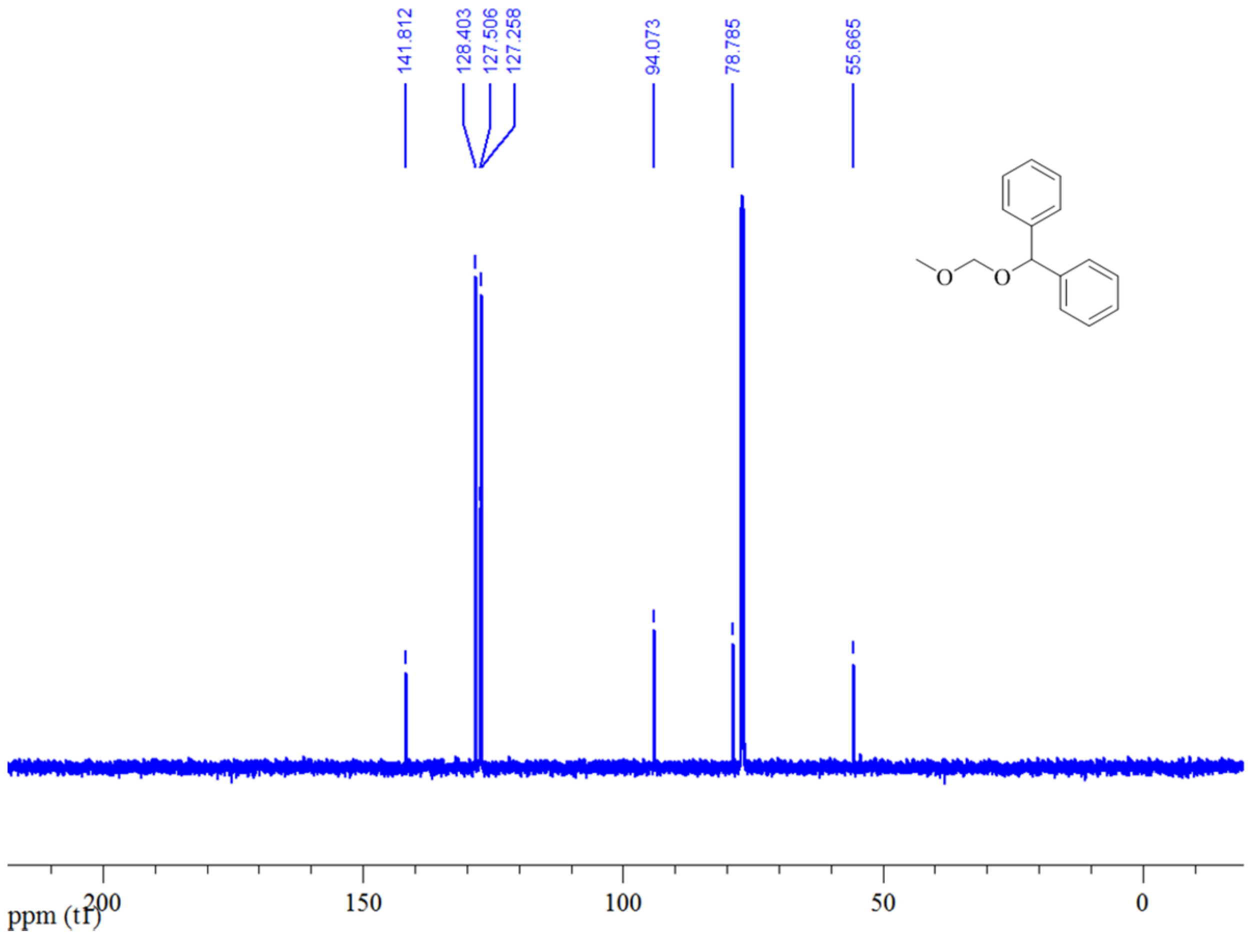

Supplementary Figure 35. ^{13}C NMR Spectra of ((Methoxymethoxy)methylene)dibenzene (**2h**).

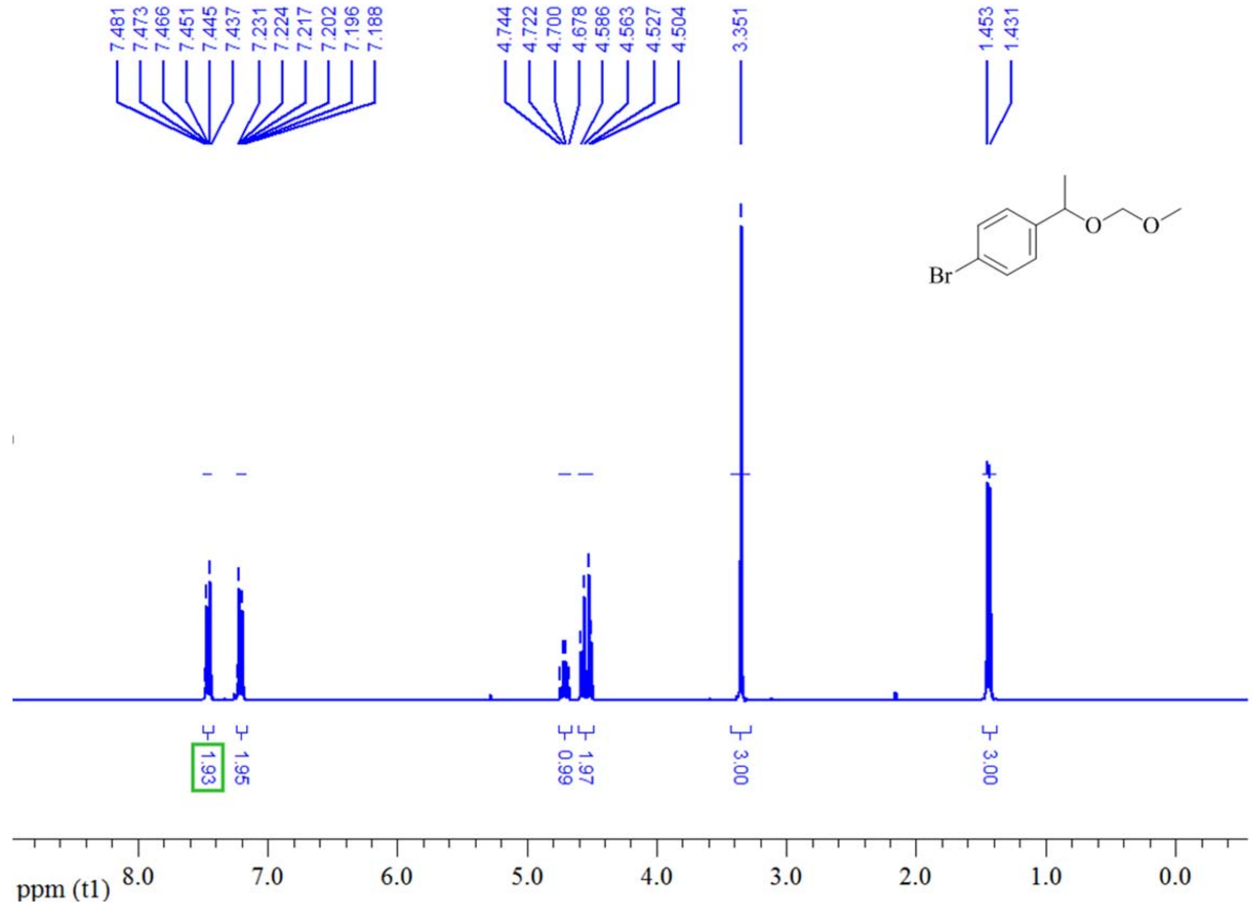

Supplementary Figure 36. ^{1}H NMR Spectra of 1-Bromo-4-(1-(methoxymethoxy)ethyl)benzene (**2i**).

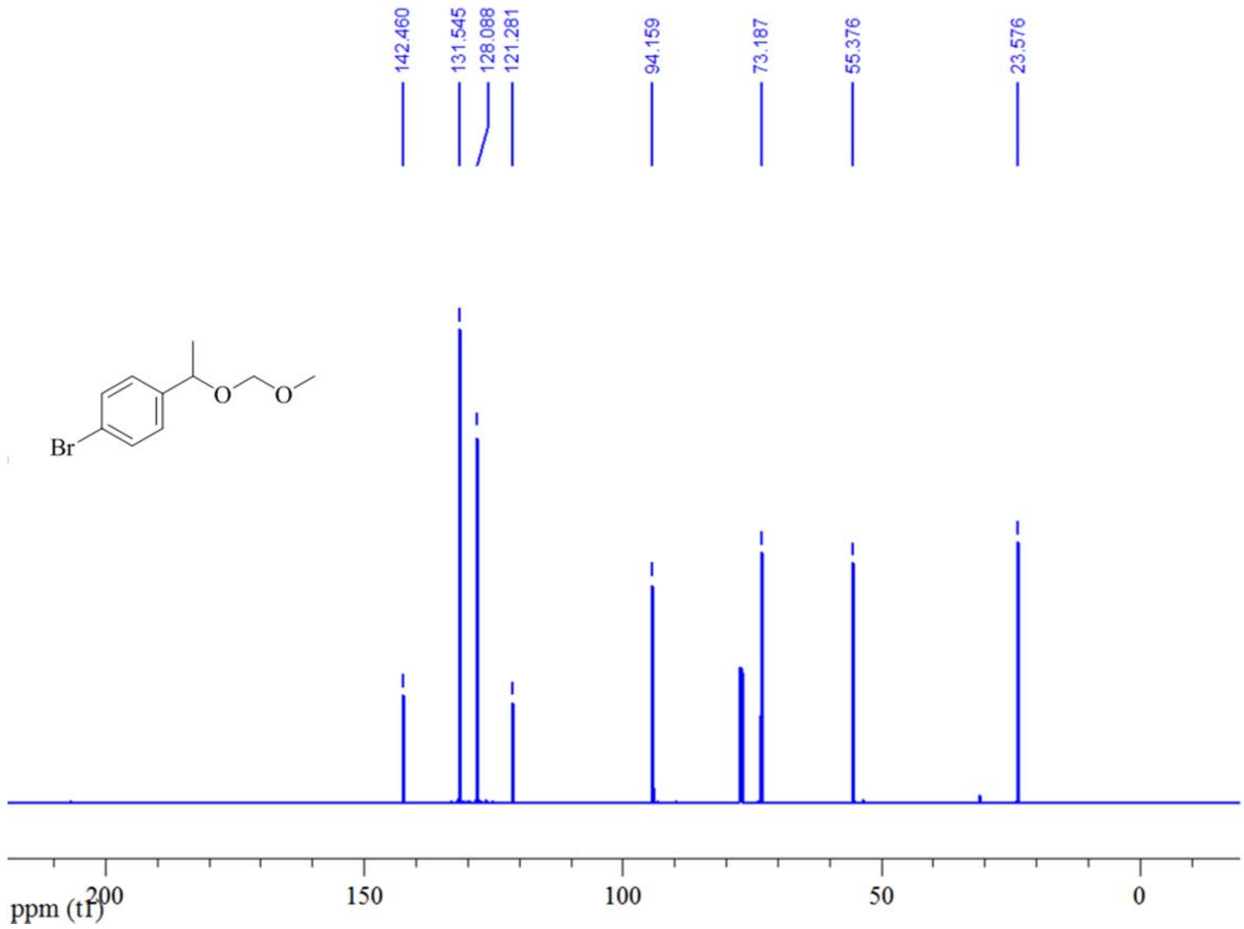

Supplementary Figure 37. ^{13}C NMR Spectra of 1-Bromo-4-(1-(methoxymethoxy)ethyl)benzene (**2i**).

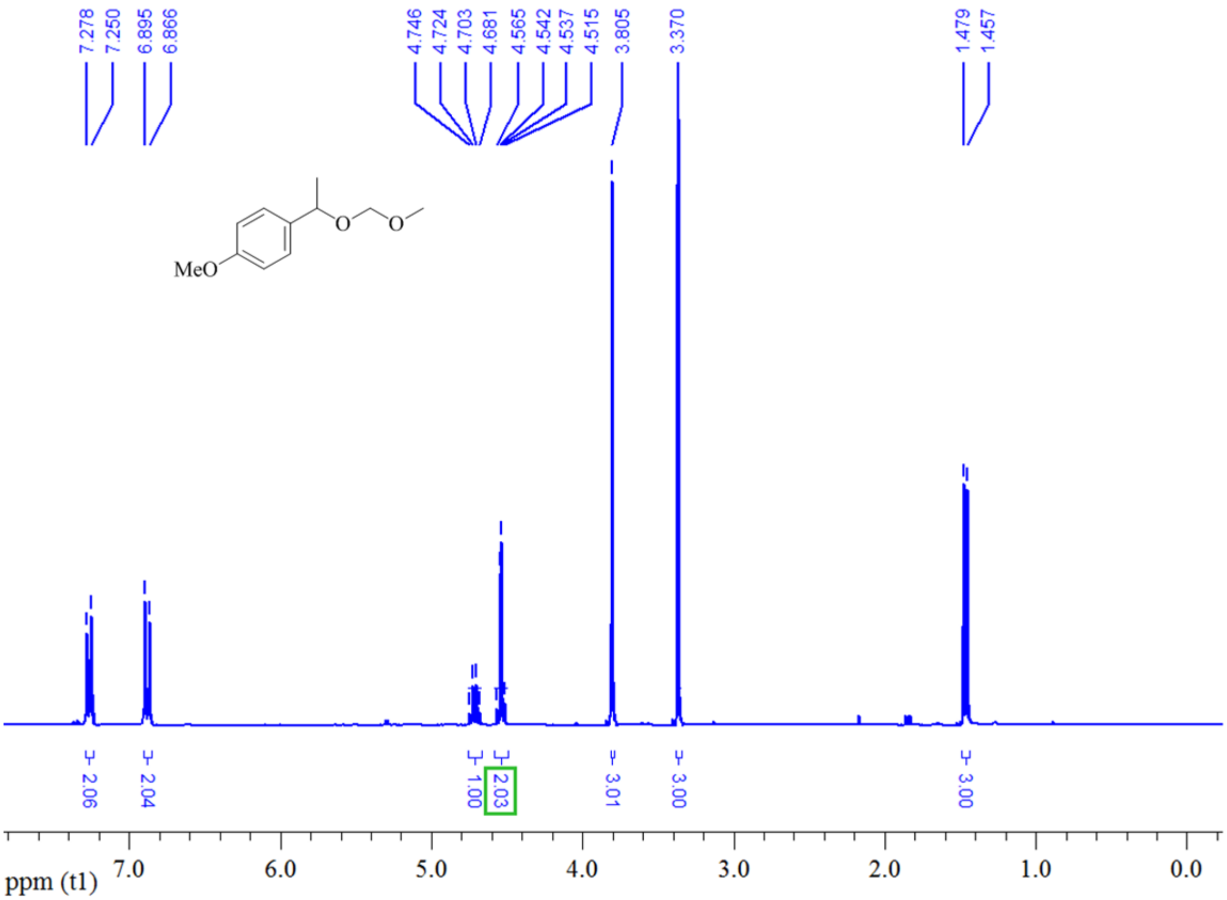

Supplementary Figure 38. ^{1}H NMR Spectra of 1-Methoxy-4-(1-(methoxymethoxy)ethyl)benzene (**2j**).

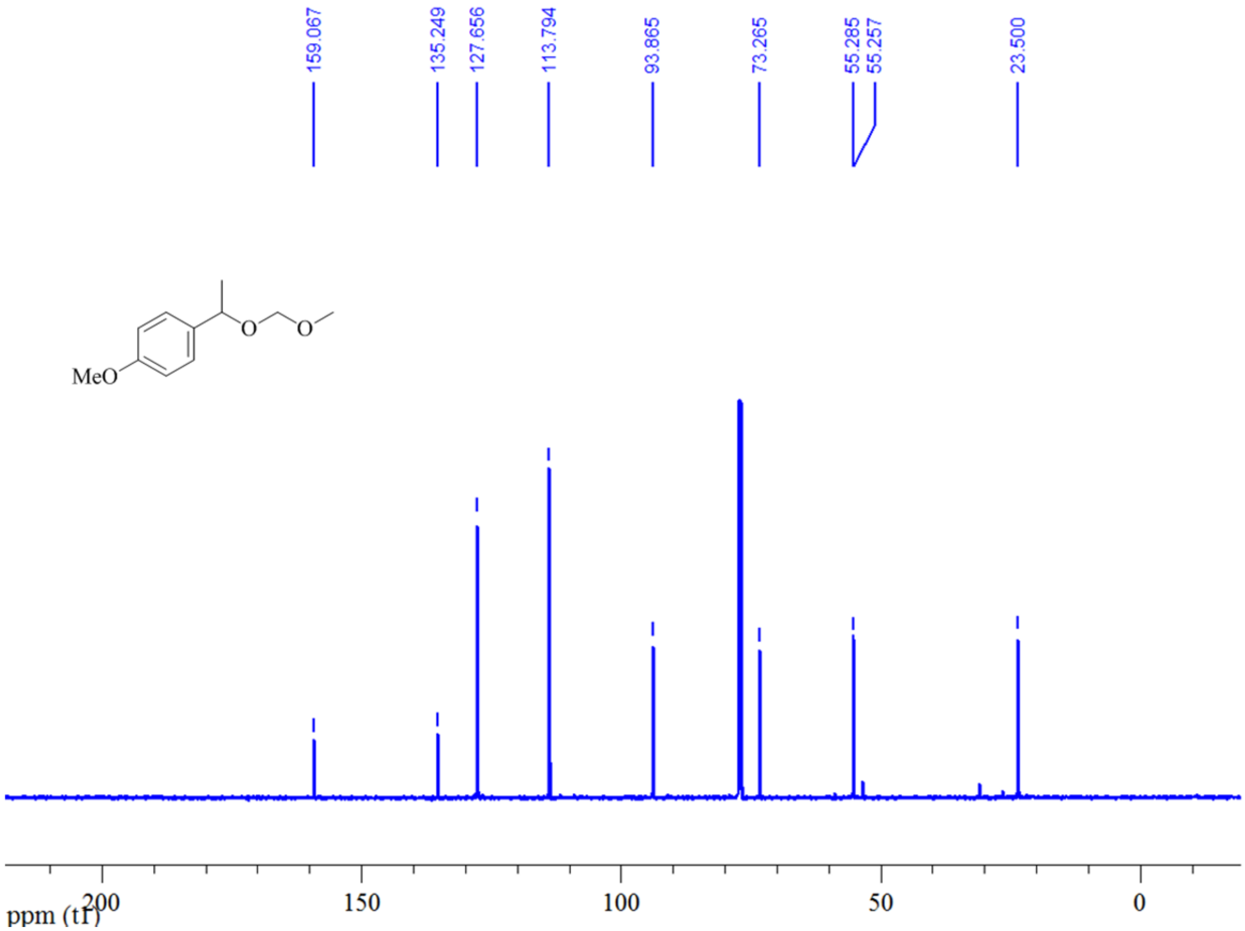

Supplementary Figure 39. ^{13}C NMR Spectra of 1-Methoxy-4-(1-(methoxymethoxy)ethyl)benzene (**2j**).

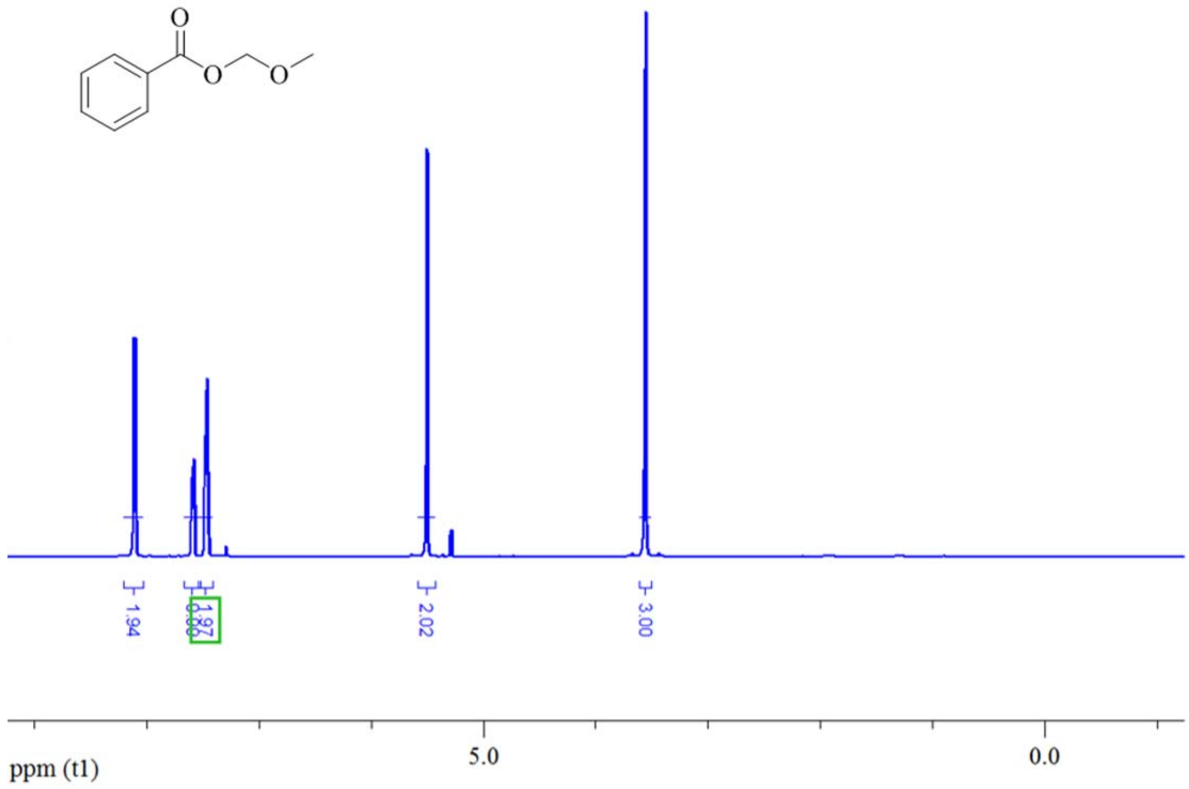

Supplementary Figure 40. ^{1}H NMR Spectra of Methoxymethyl benzoate (**2k**).

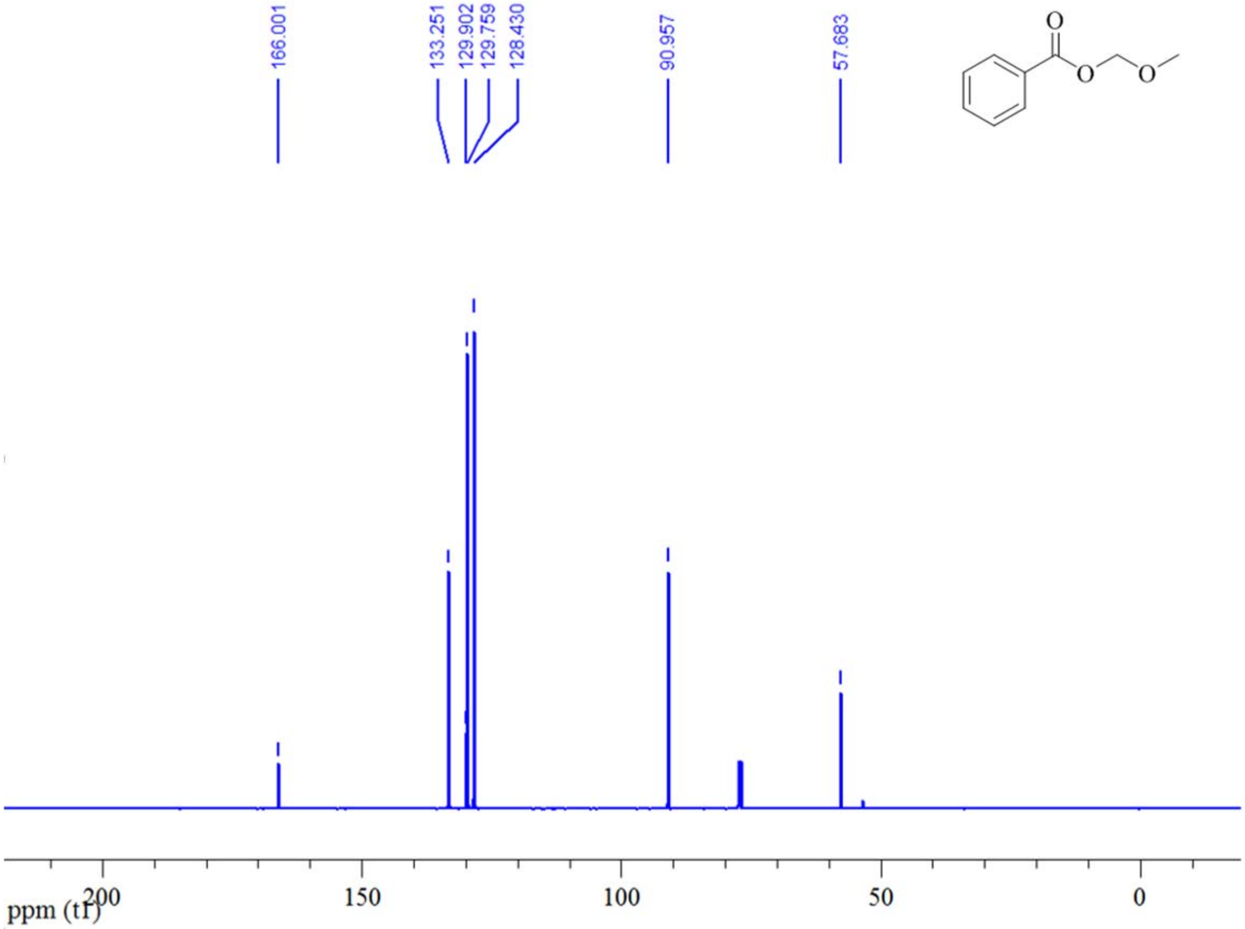

Supplementary Figure 41. ^{13}C NMR Spectra of Methoxymethyl benzoate (**2k**).

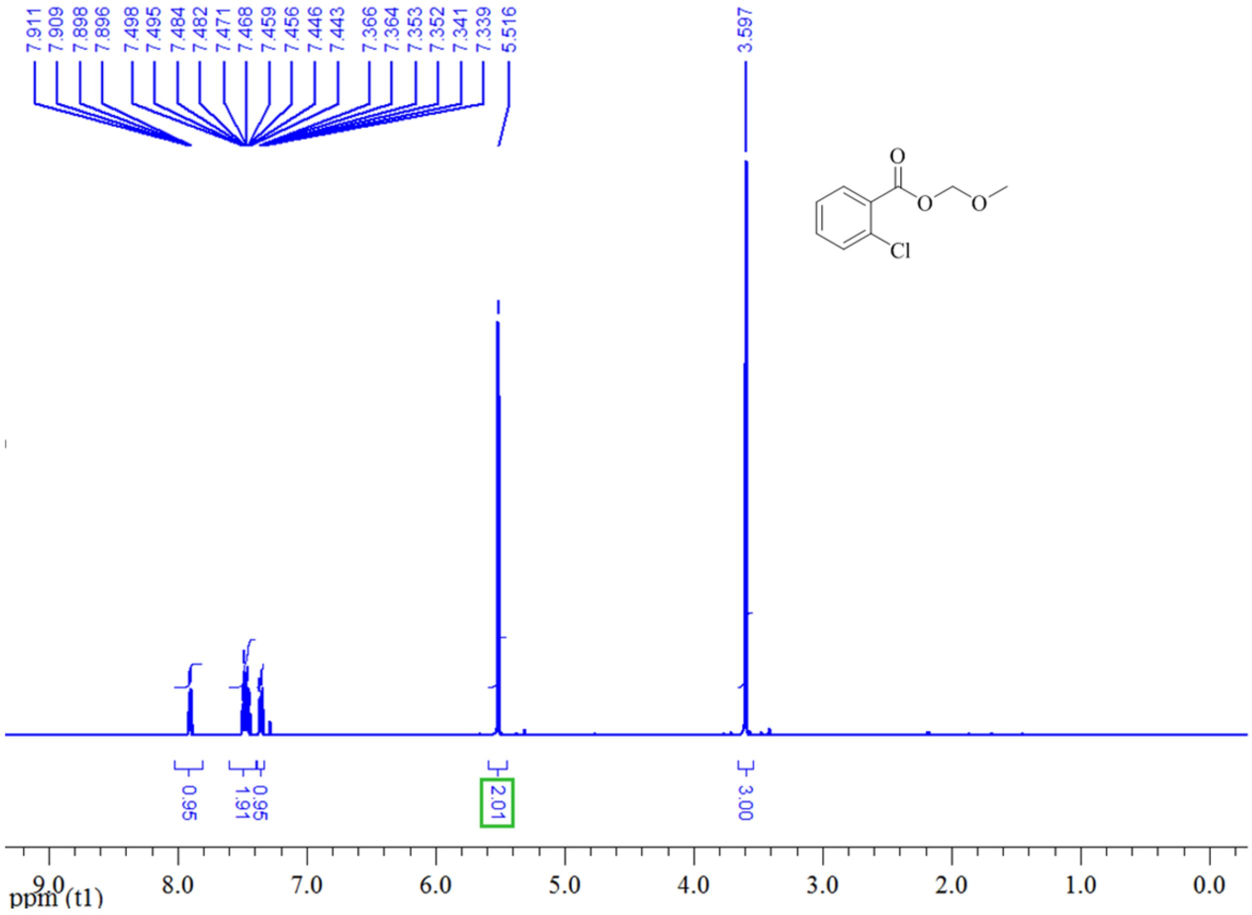

Supplementary Figure 42. ^{1}H NMR Spectra of Methoxymethyl 2-chlorobenzoate (**2l**).

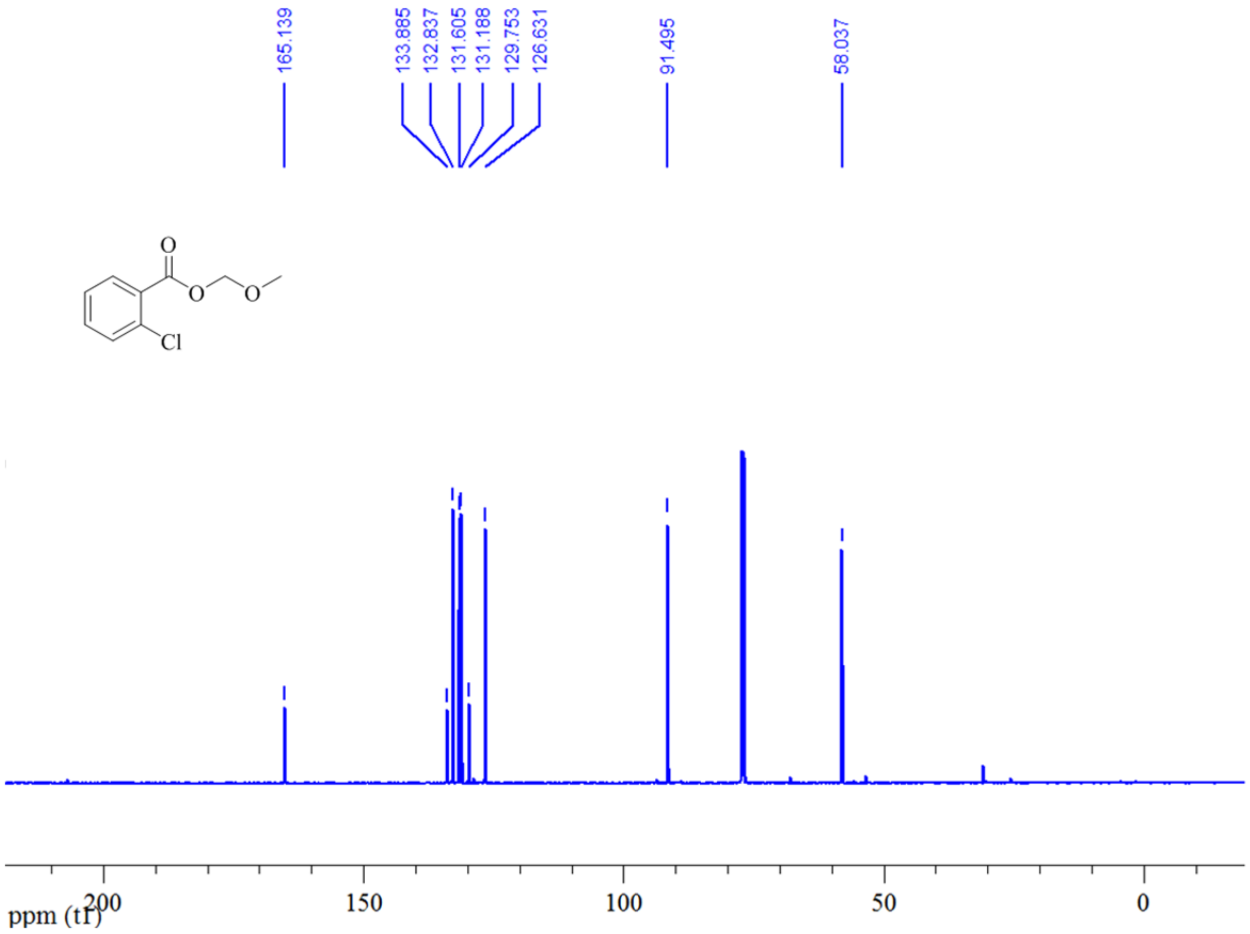

Supplementary Figure 43. ^{13}C NMR Spectra of Methoxymethyl 2-chlorobenzoate (**2l**).

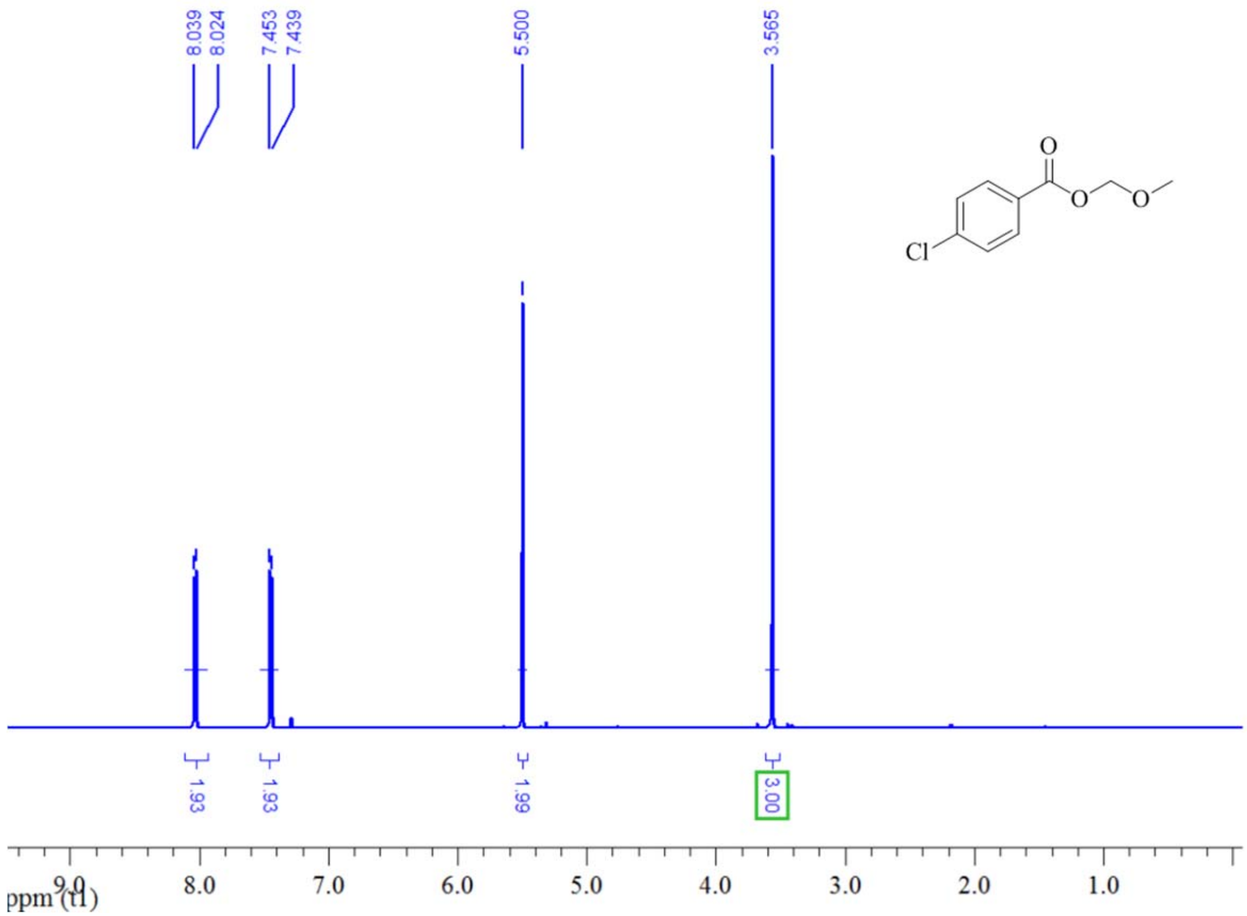

Supplementary Figure 44. ^{1}H NMR Spectra of Methoxymethyl 4-chlorobenzoate (**2m**).

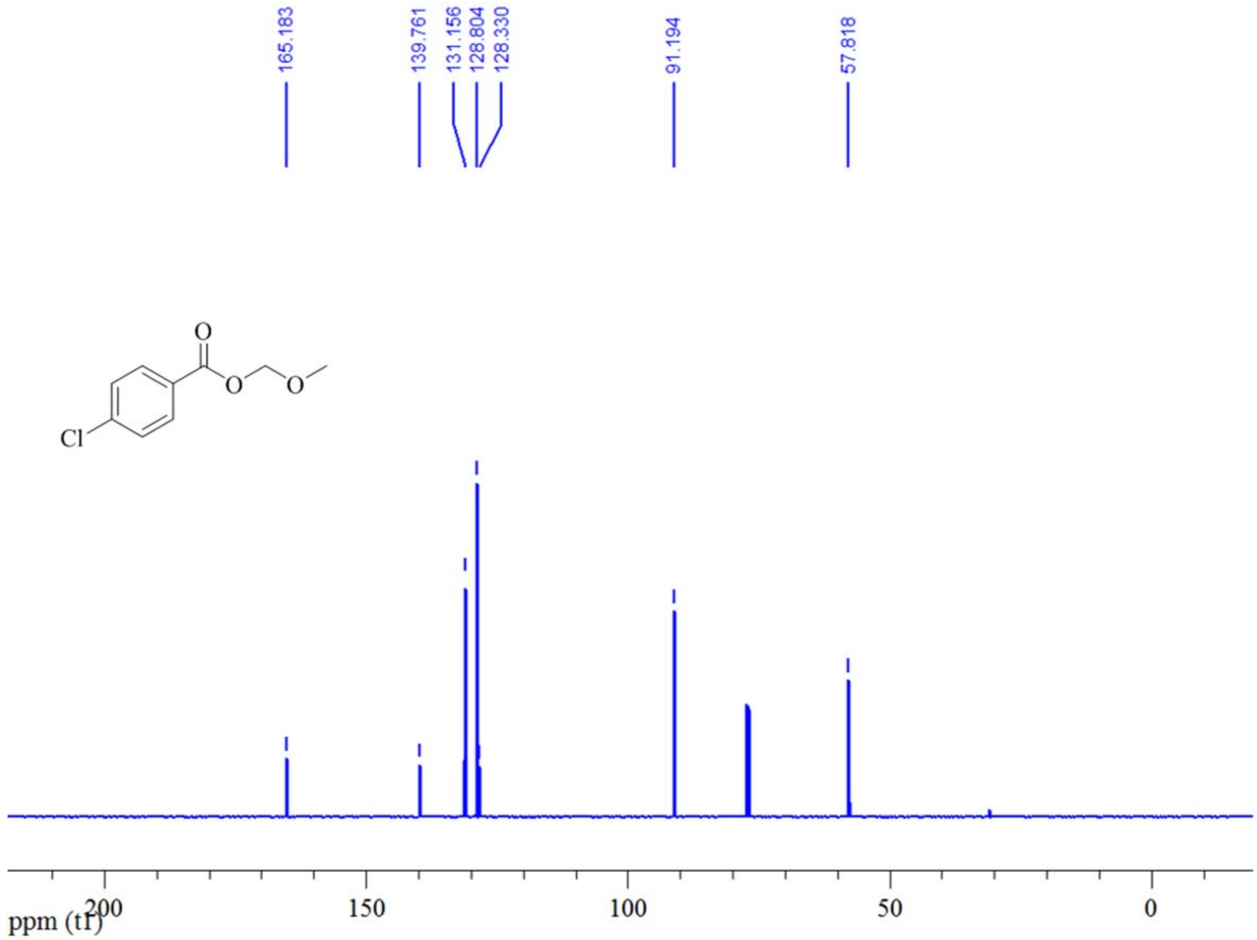

Supplementary Figure 45. ^{13}C NMR Spectra of Methoxymethyl 4-chlorobenzoate (**2m**).

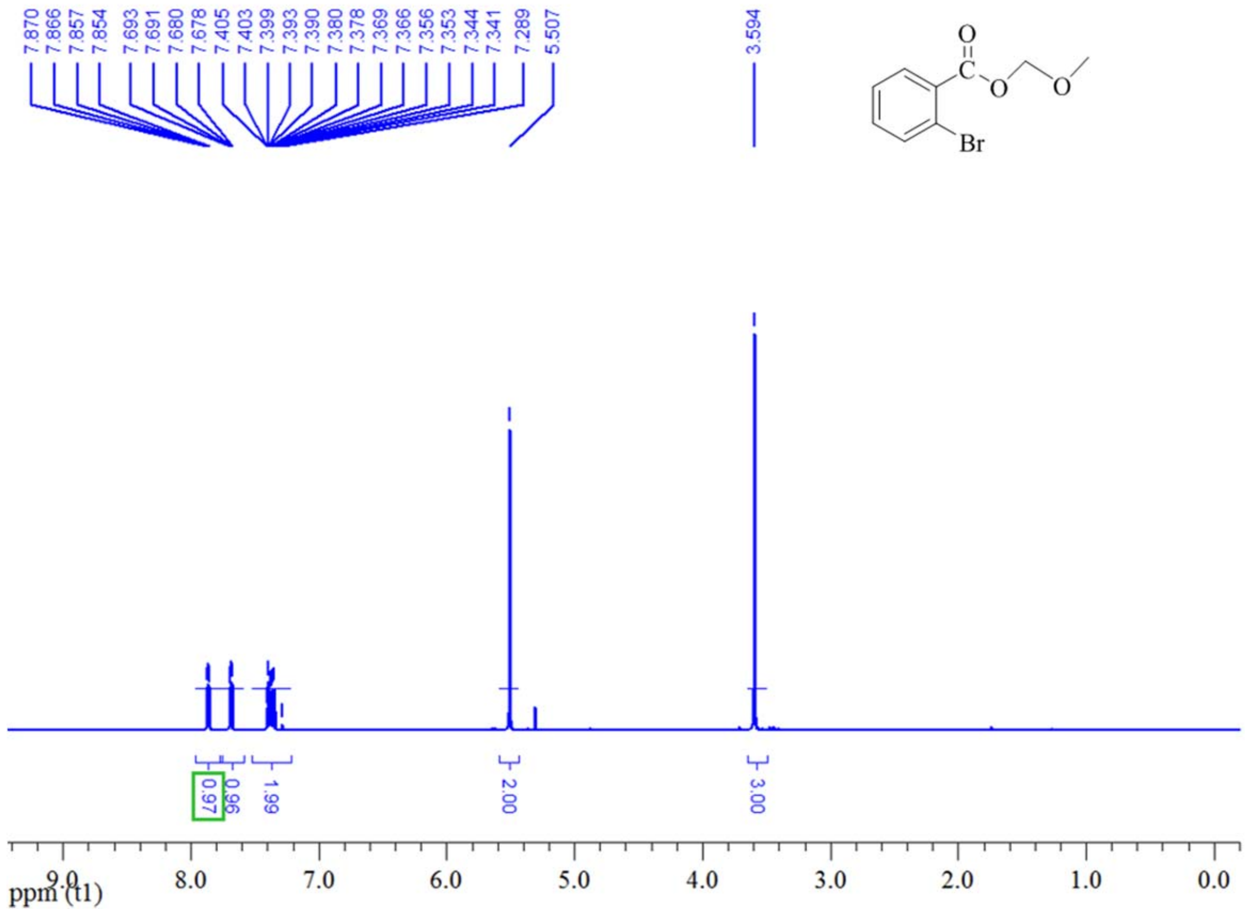

Supplementary Figure 46. ^{1}H NMR Spectra of Methoxymethyl 2-bromobenzoate (**2n**).

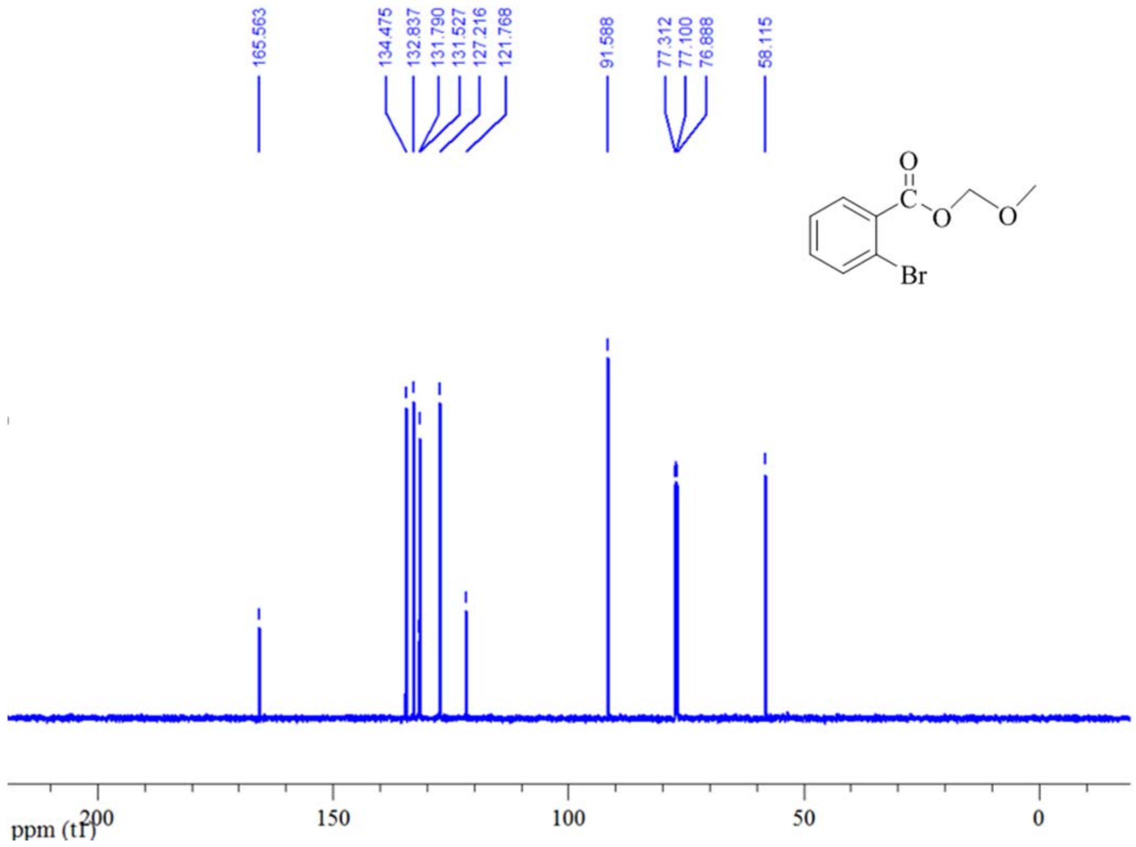

Supplementary Figure 47. ^{13}C NMR Spectra of Methoxymethyl 2-bromobenzoate (**2n**).

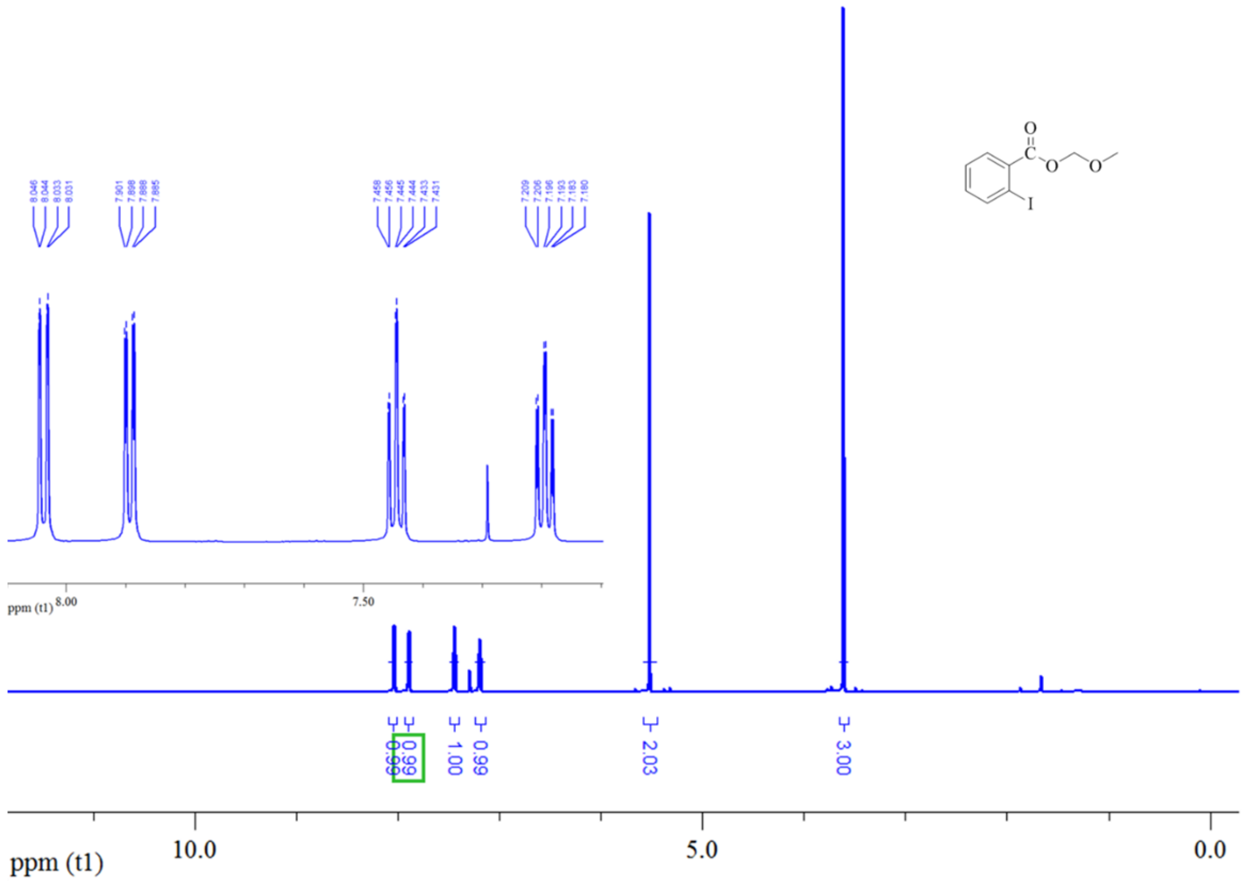

Supplementary Figure 48. ^{1}H NMR Spectra of Methoxymethyl 2-iodobenzoate (**2o**).

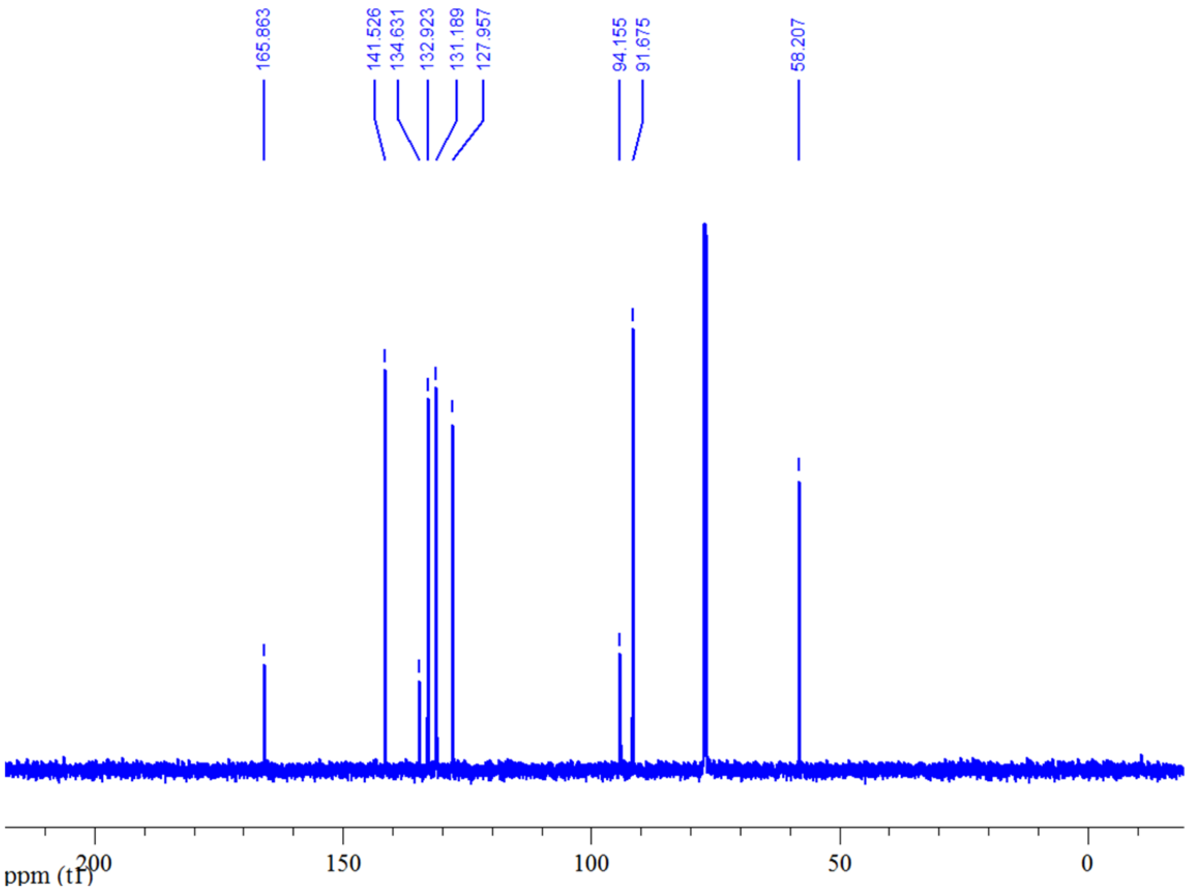

Supplementary Figure 49. ^{13}C NMR Spectra of Methoxymethyl 2-iodobenzoate (**2o**).

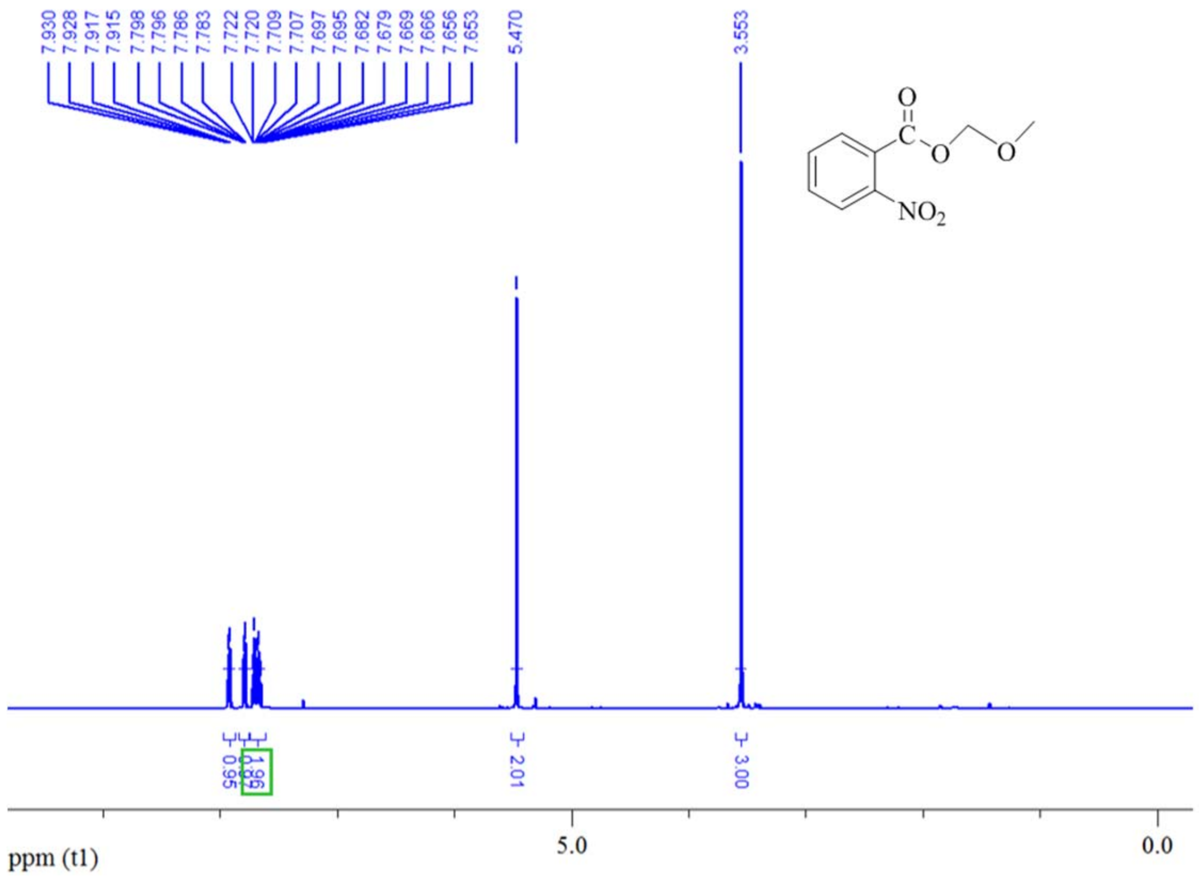

Supplementary Figure 50. ^{1}H NMR Spectra of Methoxymethyl 2-nitrobenzoate (**2p**).

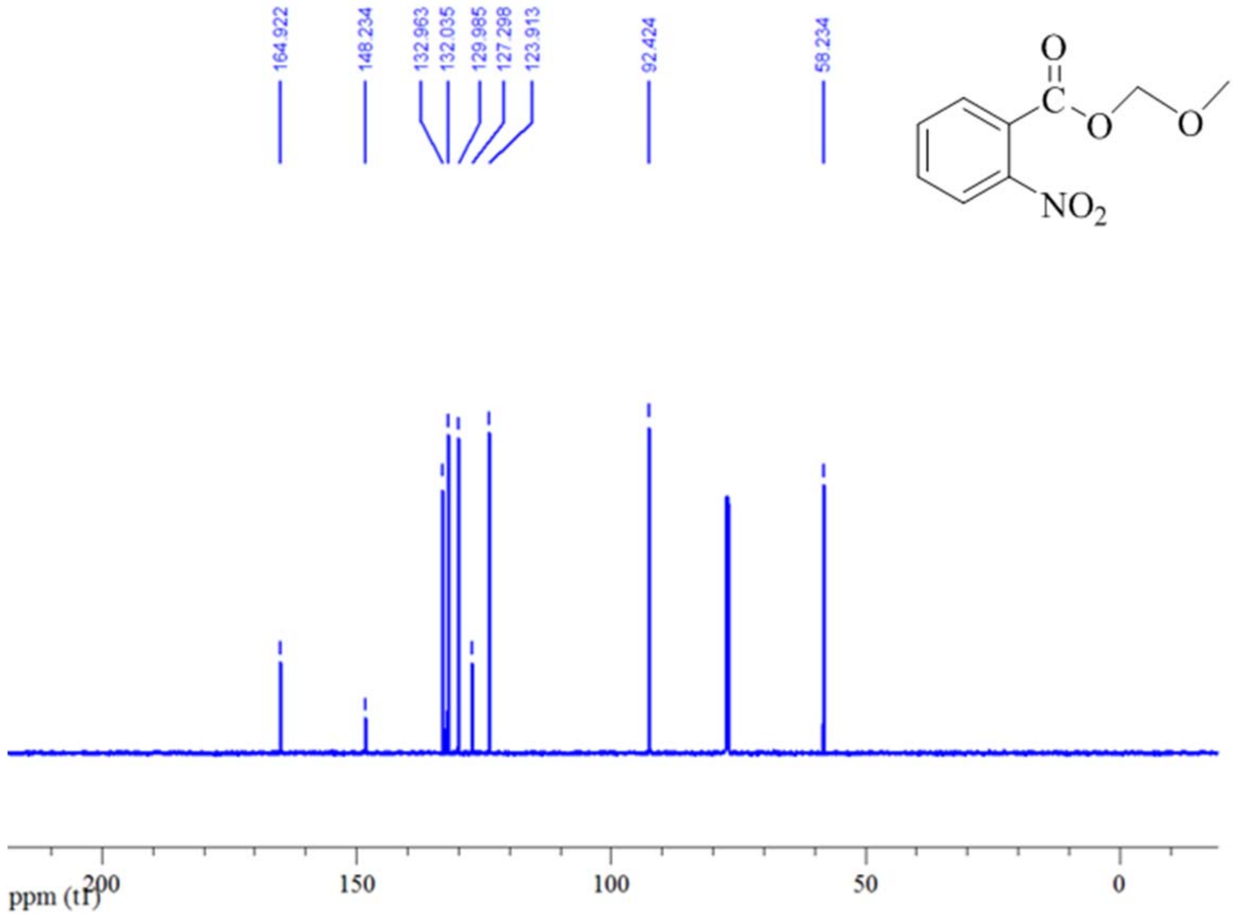

Supplementary Figure 51. ^{13}C NMR Spectra of Methoxymethyl 2-nitrobenzoate (**2p**).

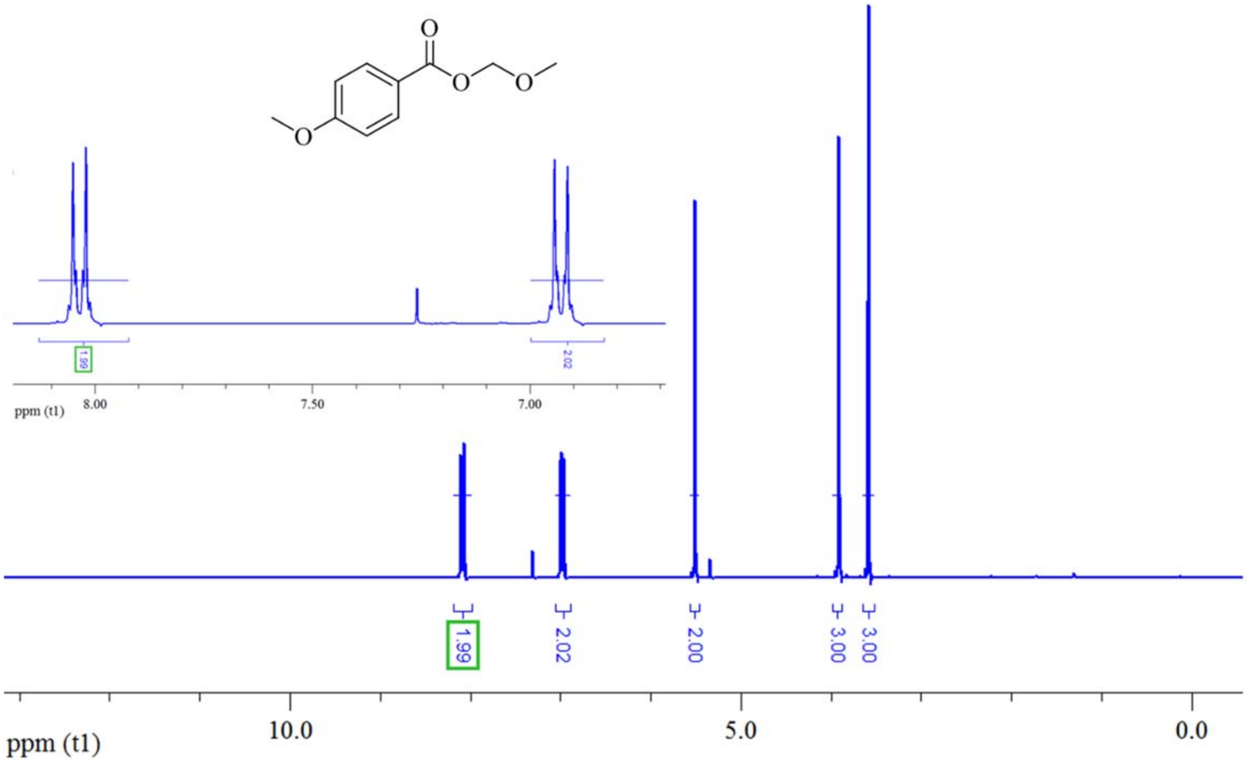

Supplementary Figure 52. ^{1}H NMR Spectra of Methoxymethyl 4-methoxybenzoate (**2q**).

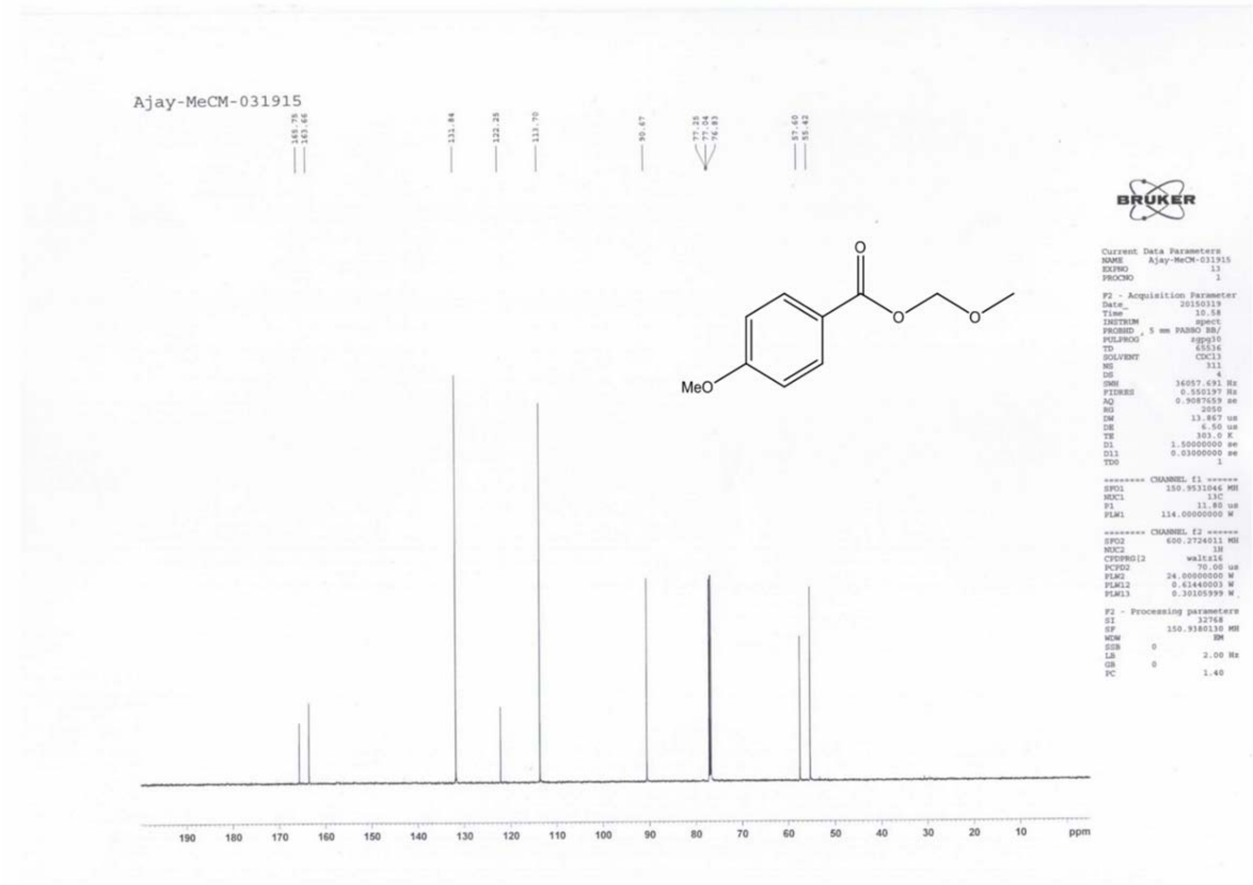

Supplementary Figure 53. ^{13}C NMR Spectra of Methoxymethyl 4-methoxybenzoate (**2q**).

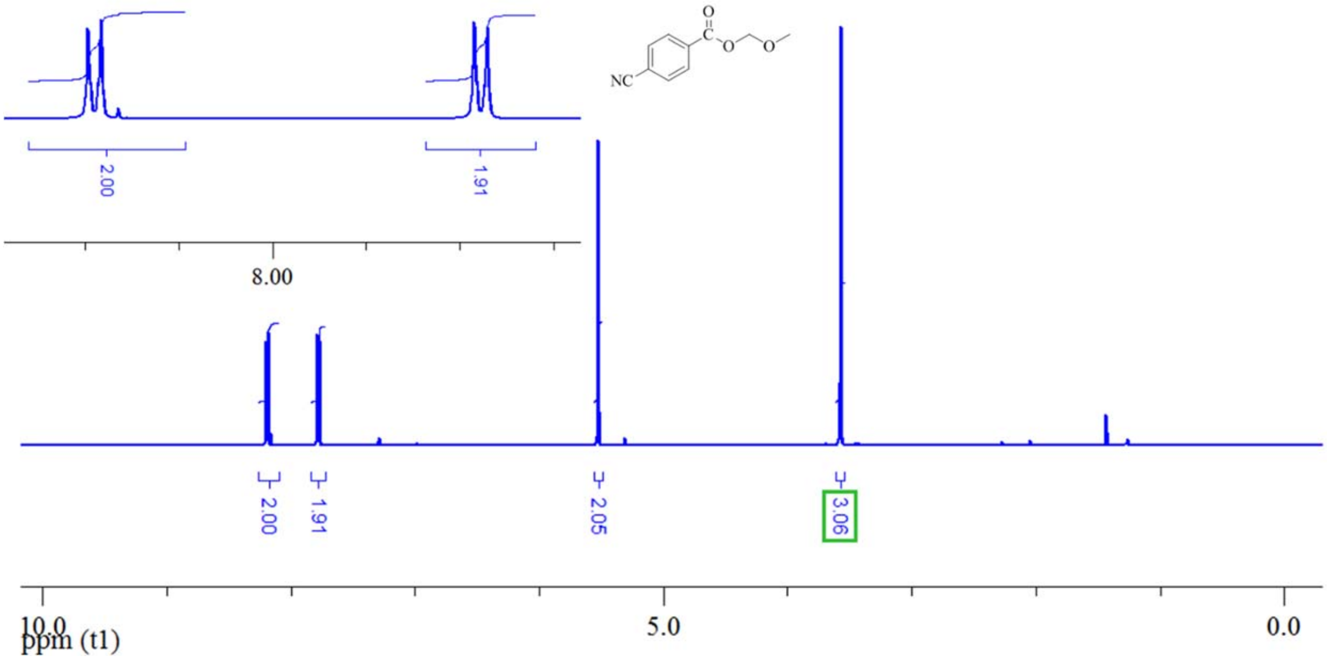

Supplementary Figure 54. ^{1}H NMR Spectra of Methoxymethyl 4-cyanobenzoate (**2r**).

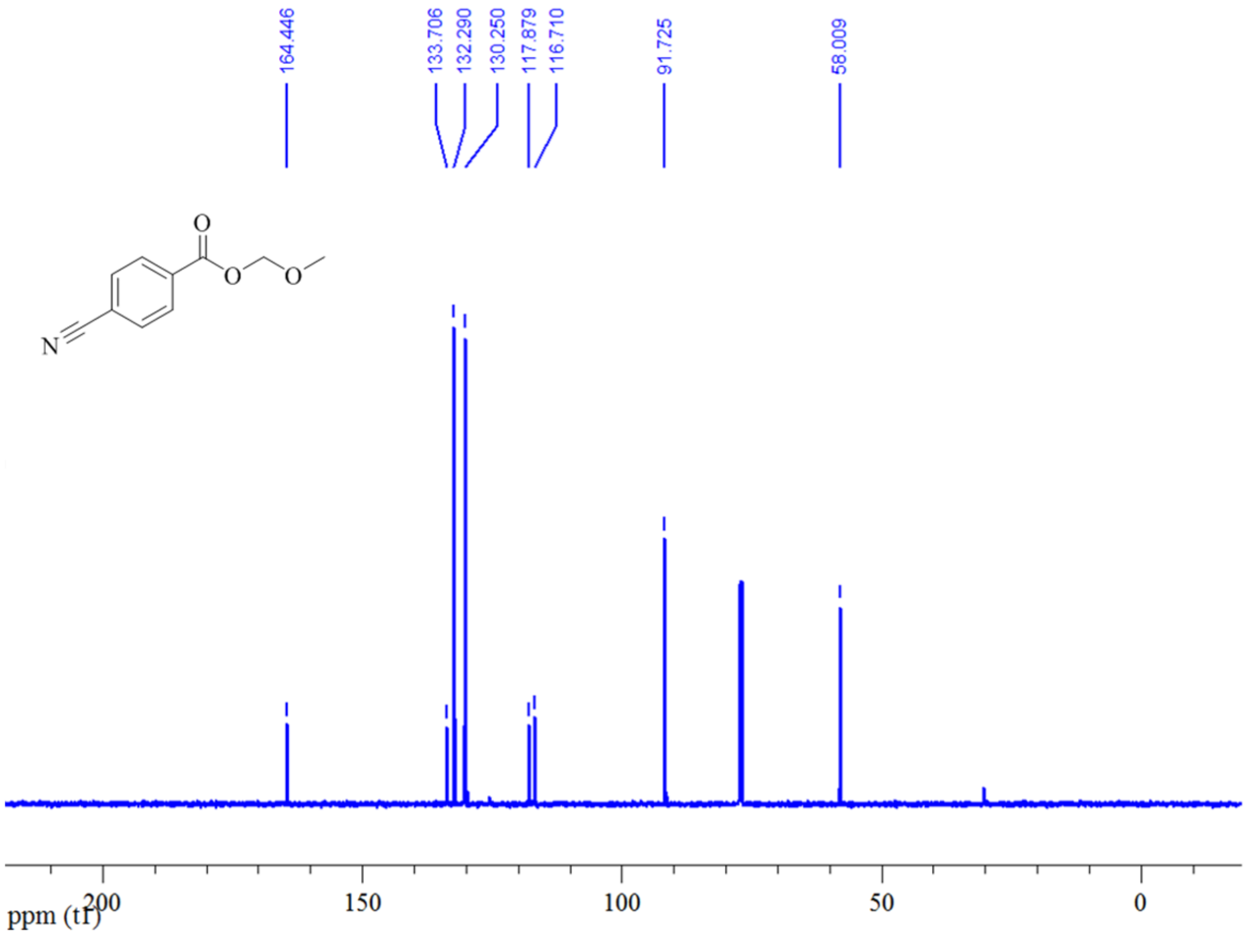

Supplementary Figure 55. ^{13}C NMR Spectra of Methoxymethyl 4-cyanobenzoate (**2r**).

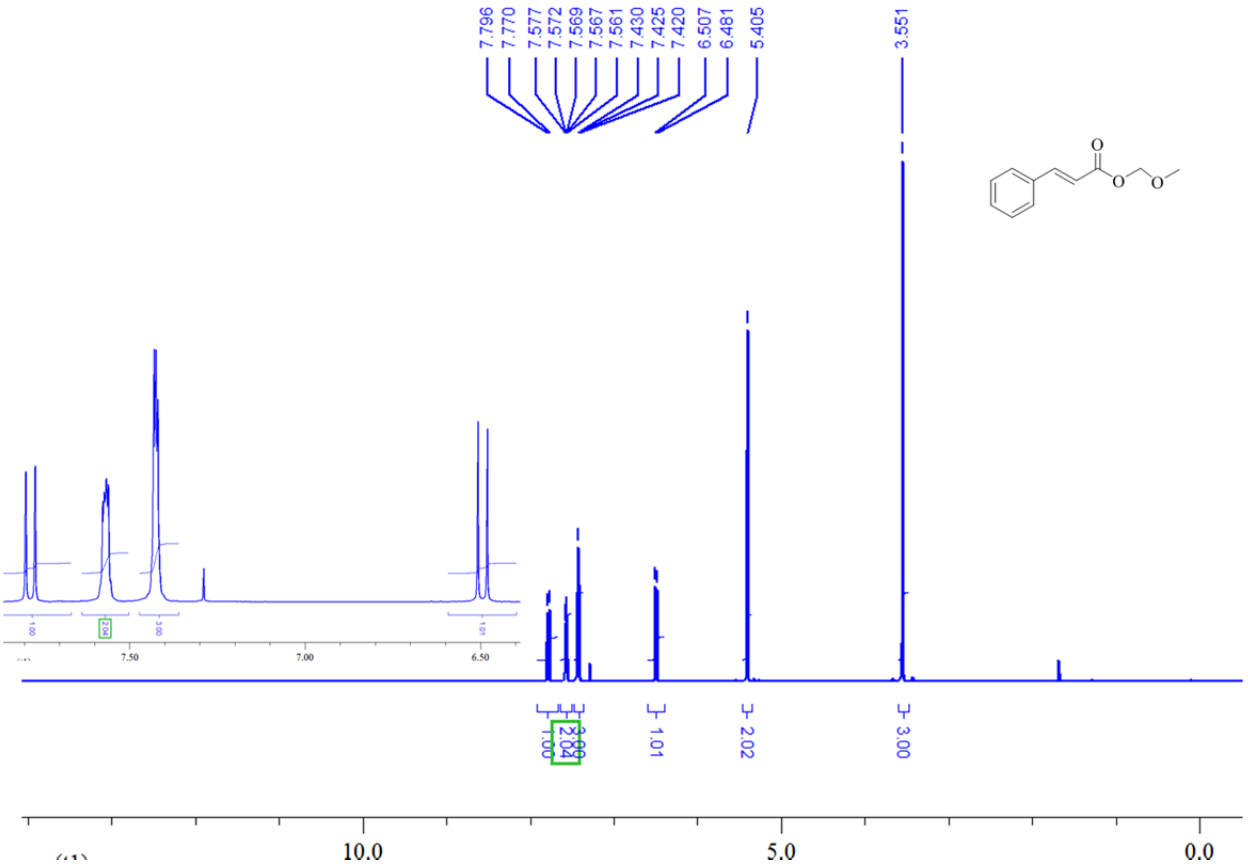

Supplementary Figure 56. ^{1}H NMR Spectra of Methoxymethylcinnamate (**2s**).

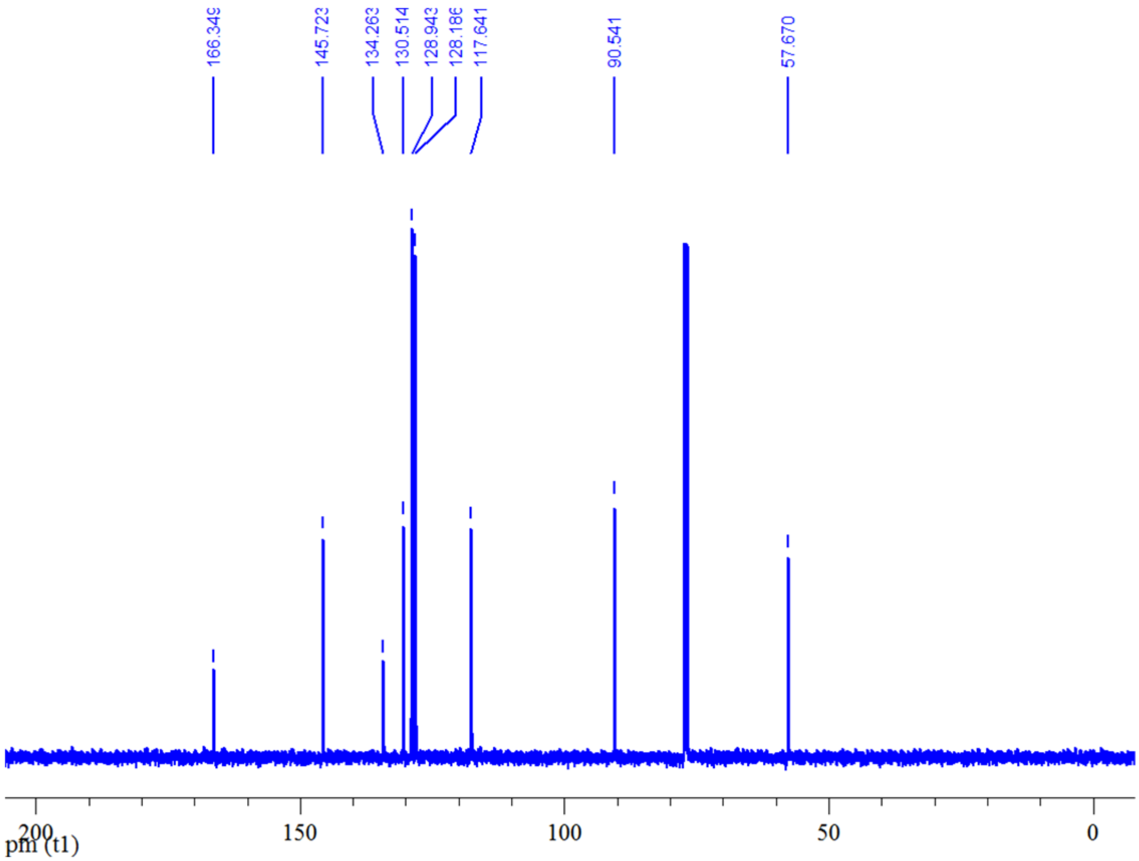

Supplementary Figure 57. ^{13}C NMR Spectra of Methoxymethylcinnamate (**2s**).

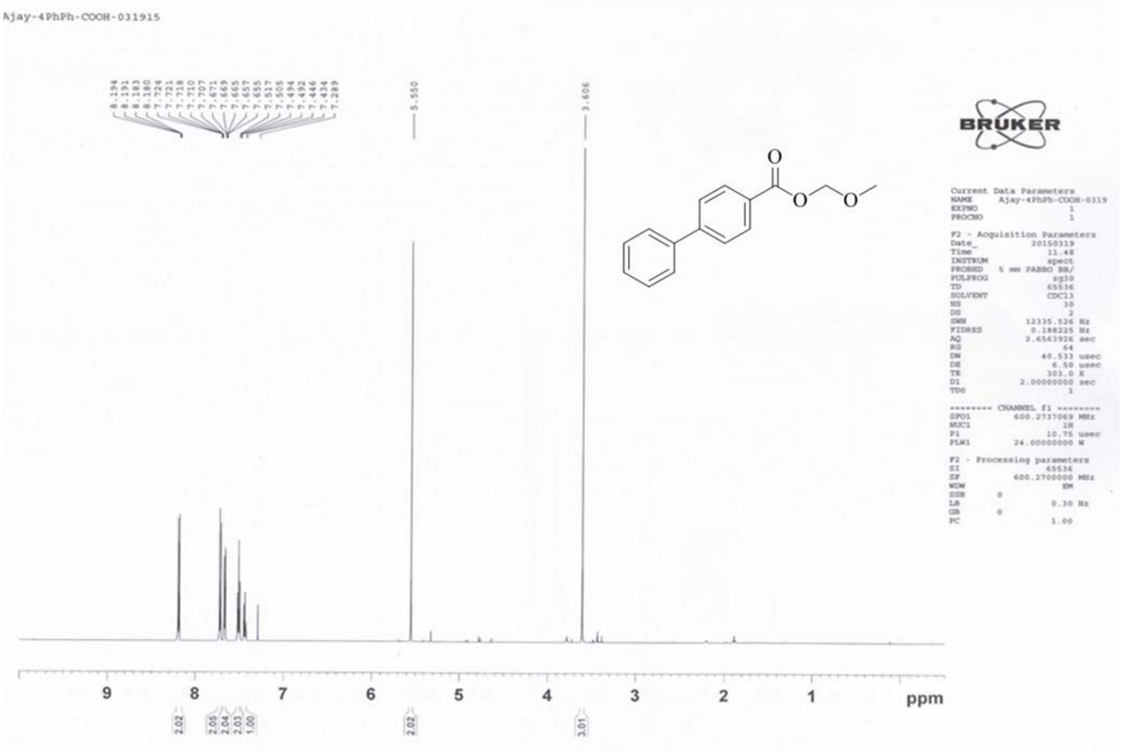

Supplementary Figure 58. ^{1}H NMR Spectra of Methoxymethyl biphenyl-4-carboxylate (**2t**).

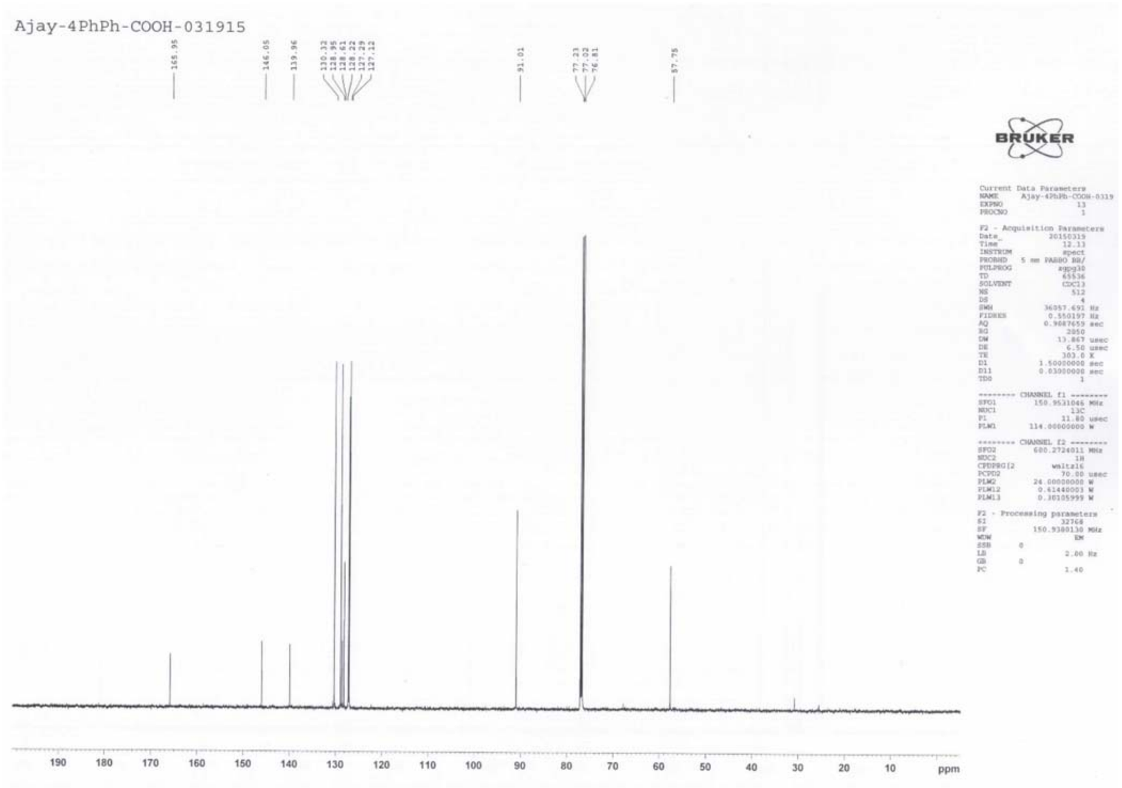

Supplementary Figure 59. ^{13}C NMR Spectra of Methoxymethyl biphenyl-4-carboxylate (2t).

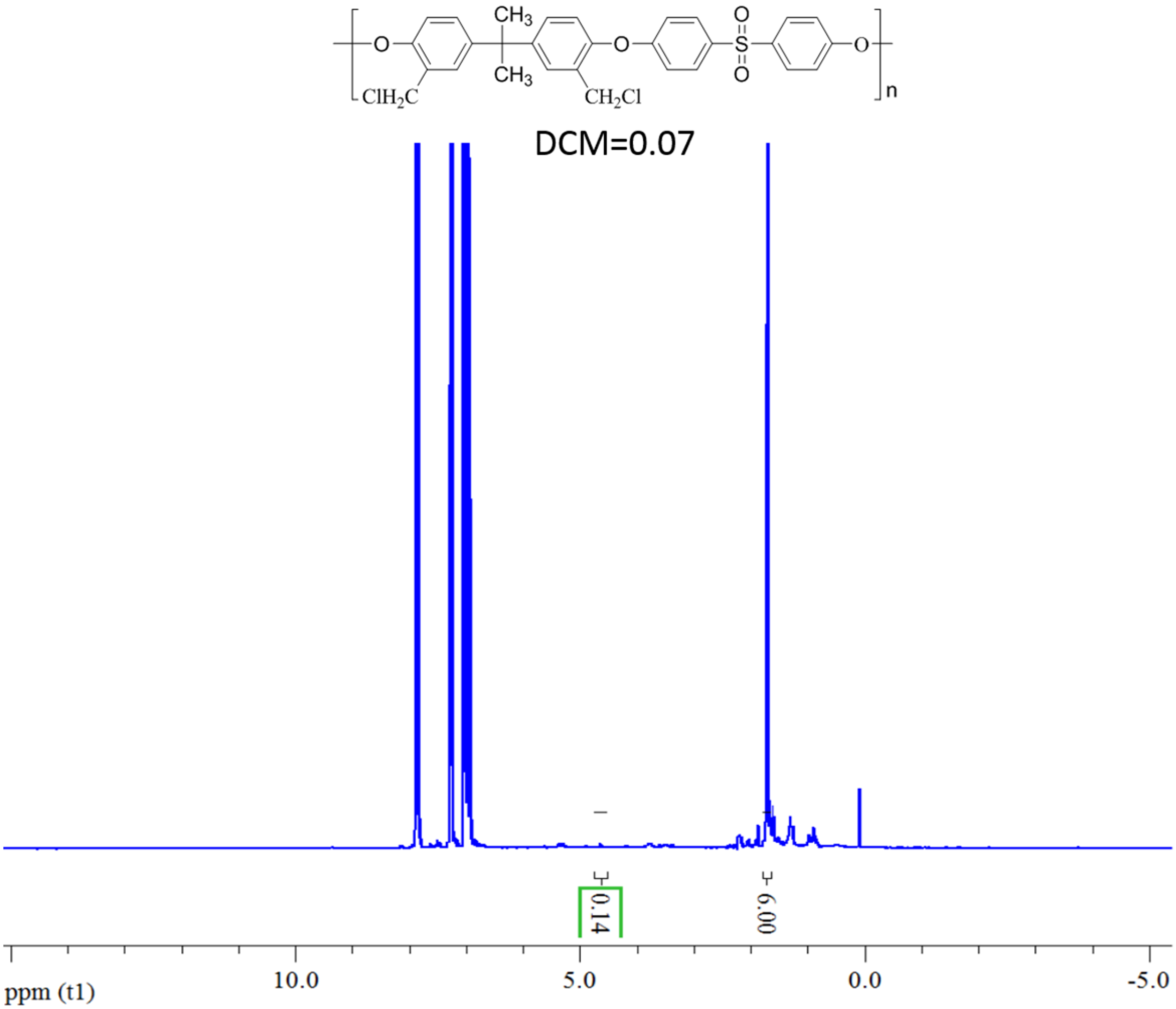

Supplementary Figure 60. ^{1}H NMR Spectra of CPS (**3a**).

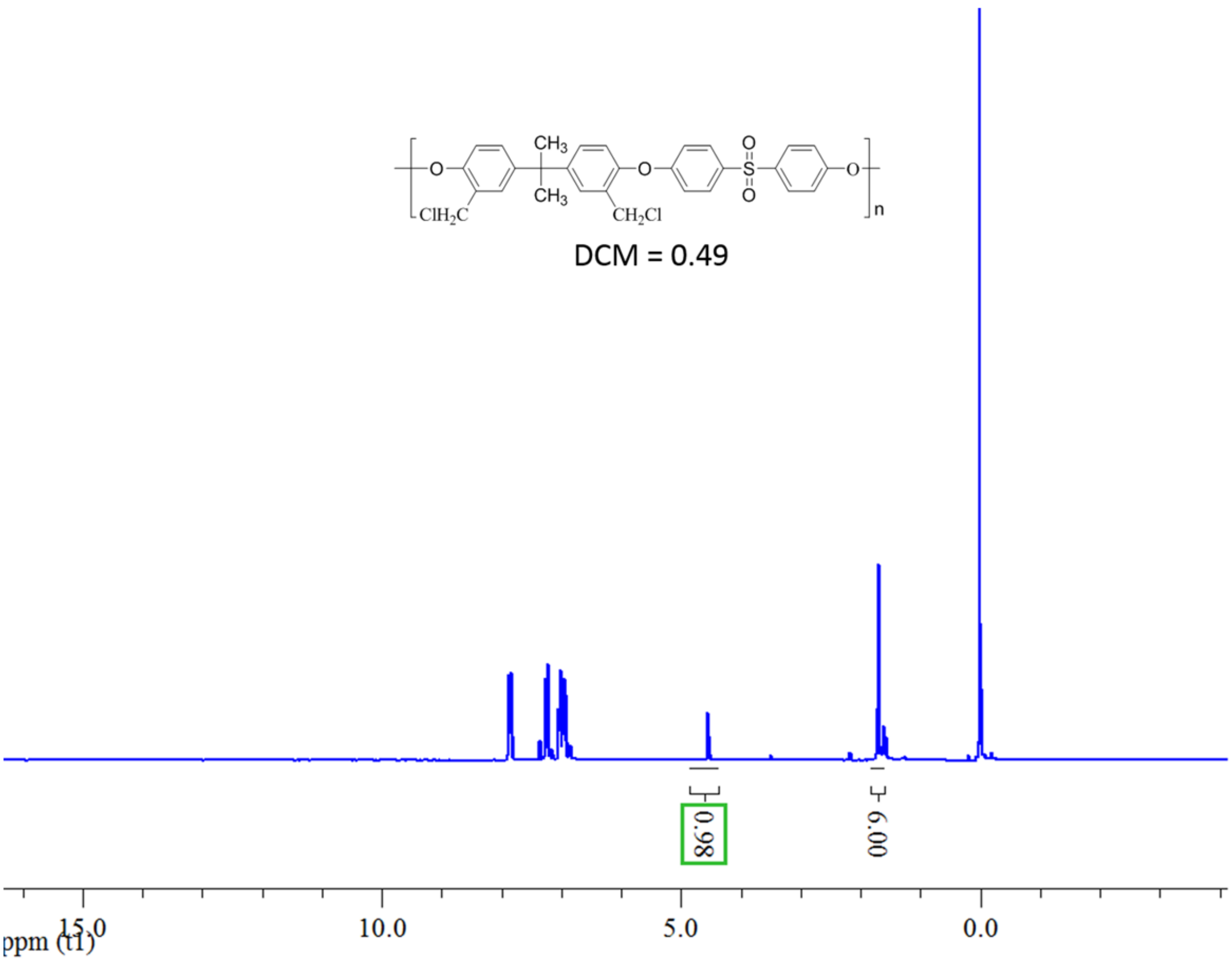

Supplementary Figure 61. ^{1}H NMR Spectra of CPS (**3b**).

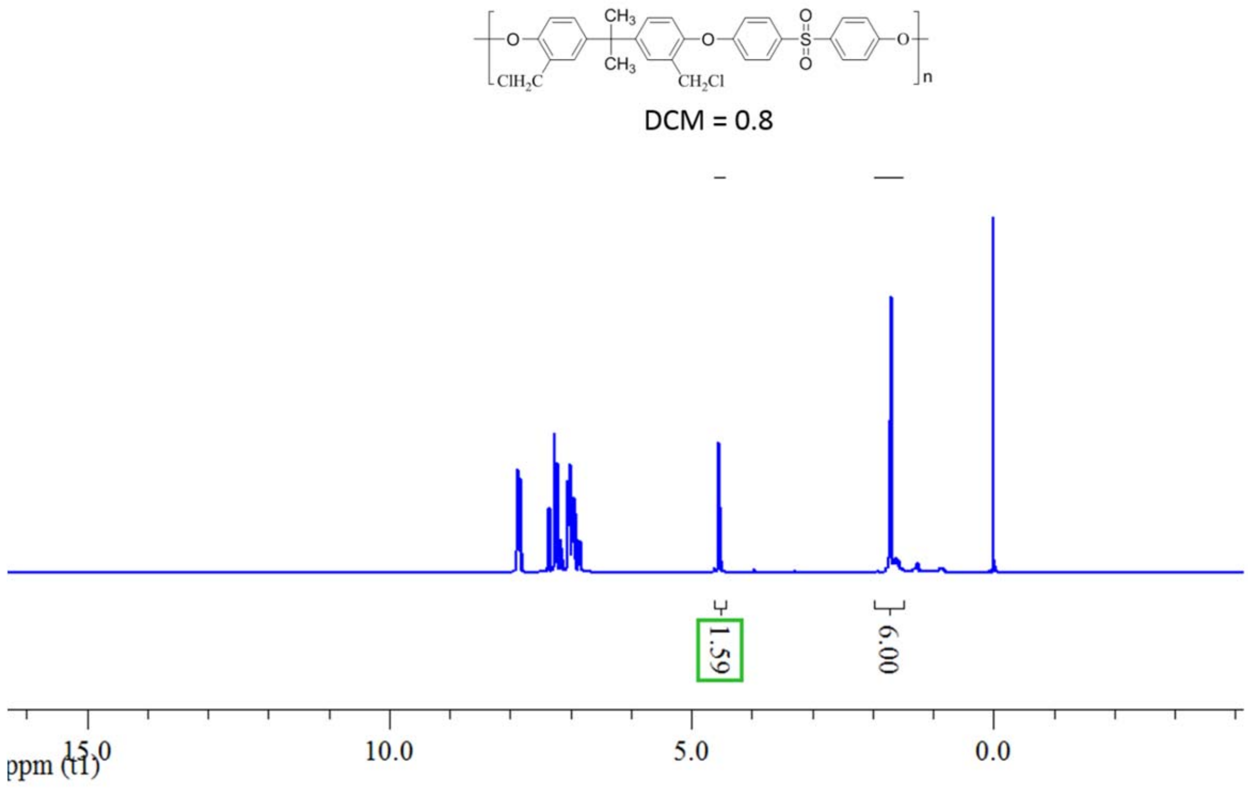

Supplementary Figure 62. ^{1}H NMR Spectra of CPS (**3c**).

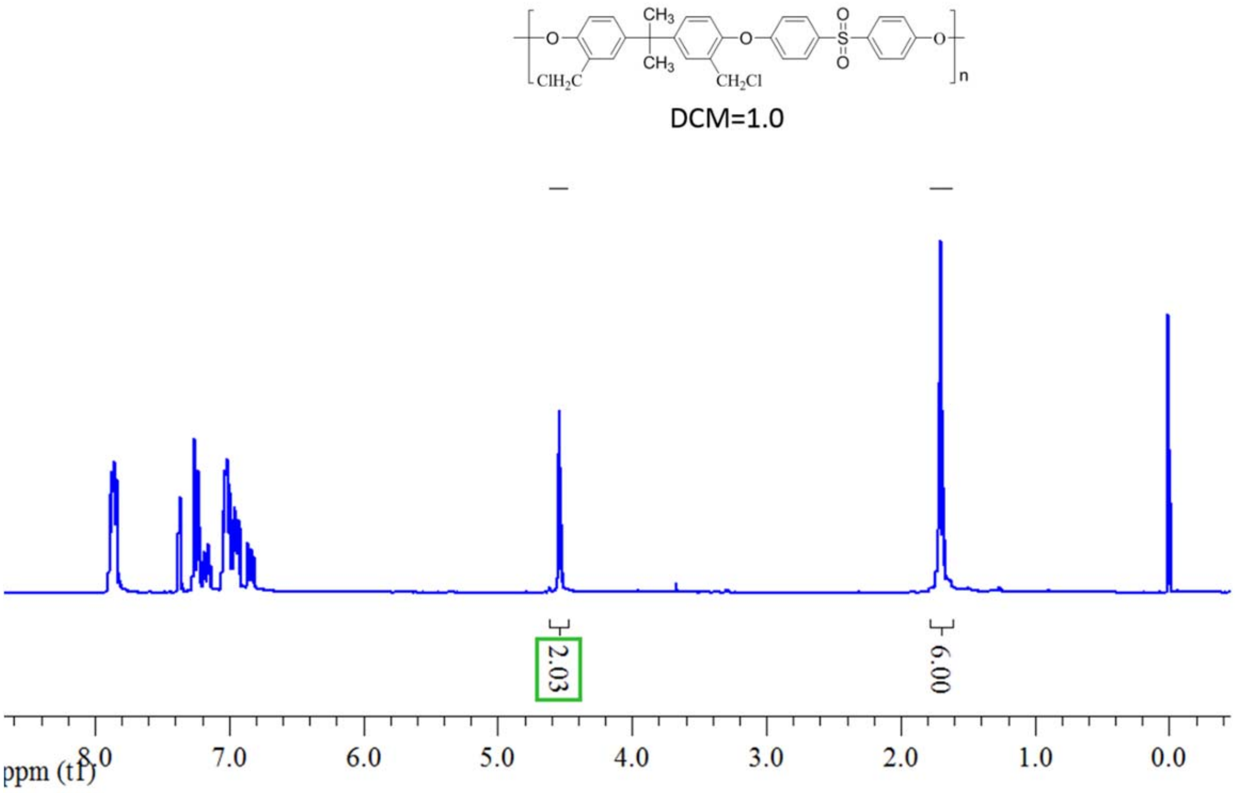

Supplementary Figure 63. ^{1}H NMR Spectra of CPS (**3d**).

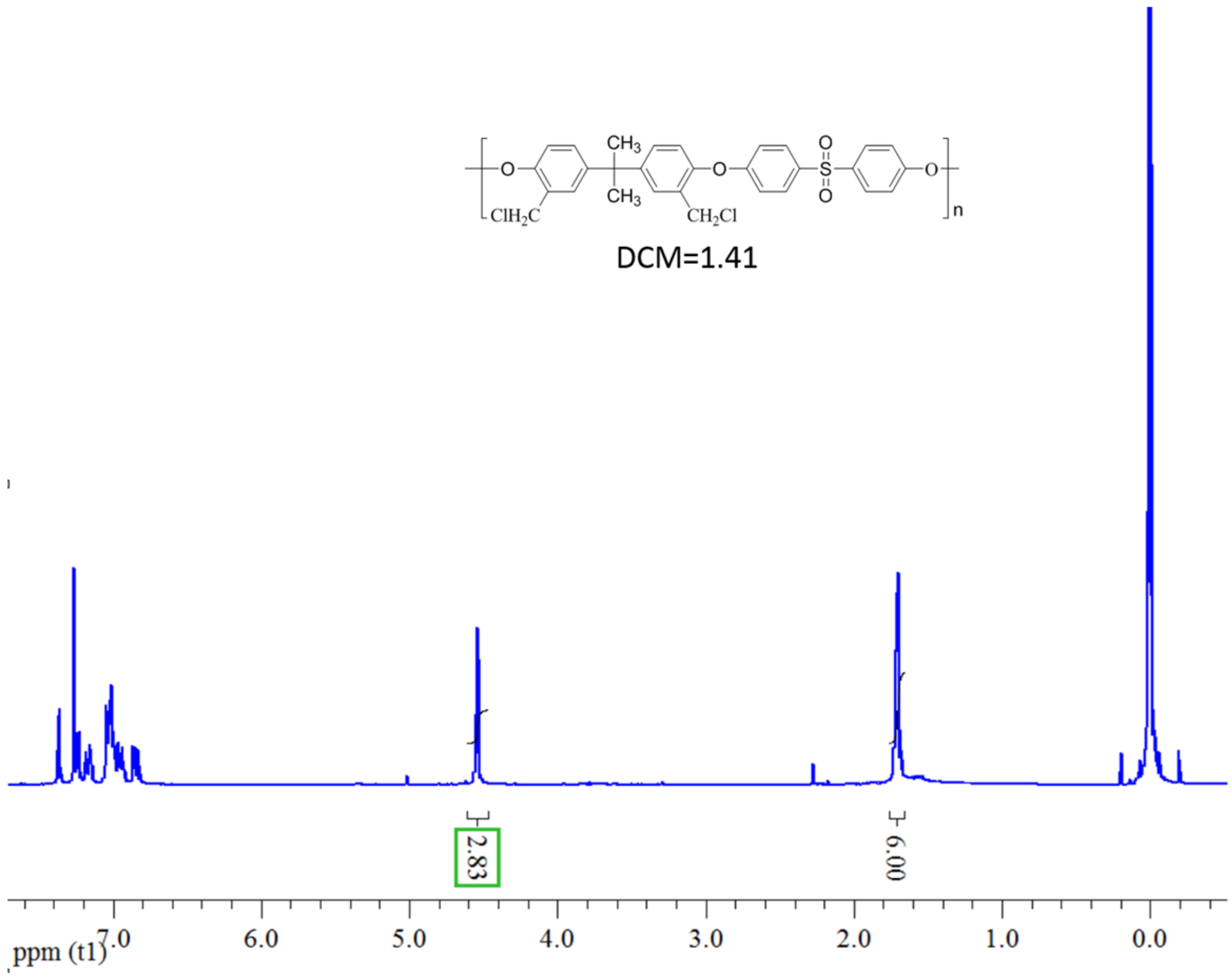

Supplementary Figure 64. ^{1}H NMR Spectra of CPS (**3e**).

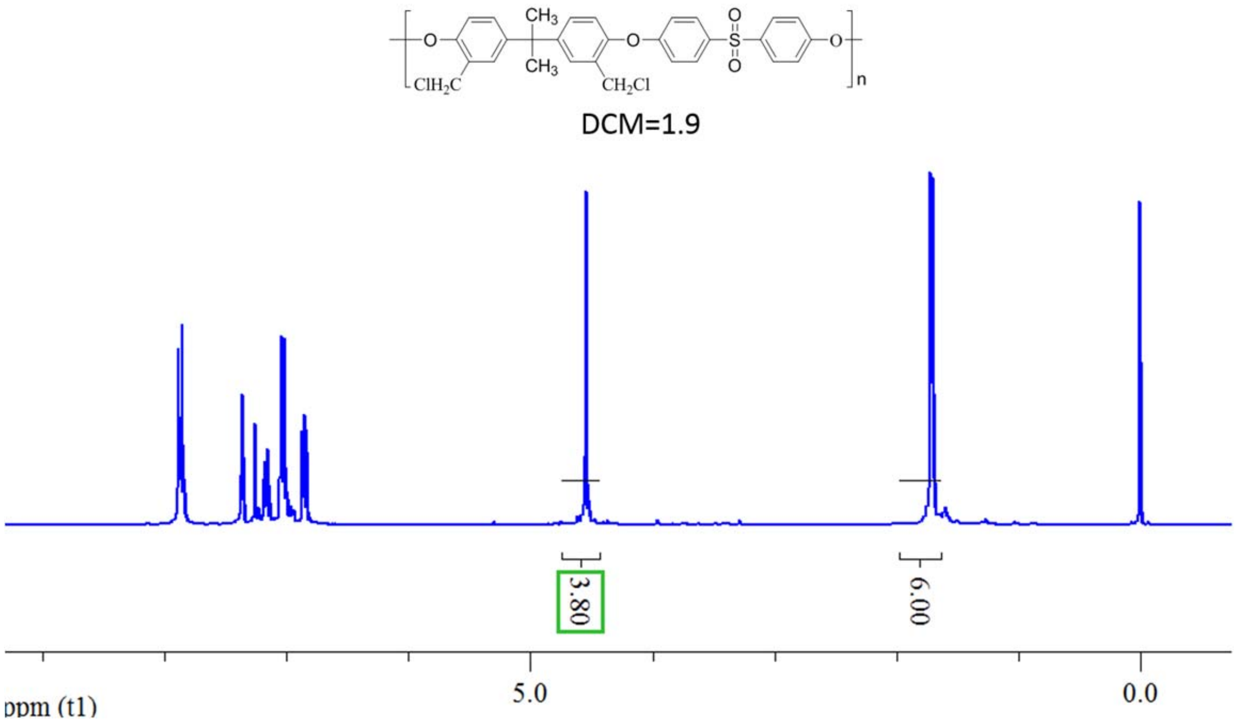

Supplementary Figure 65. ^{1}H NMR Spectra of CPS (**3f**).

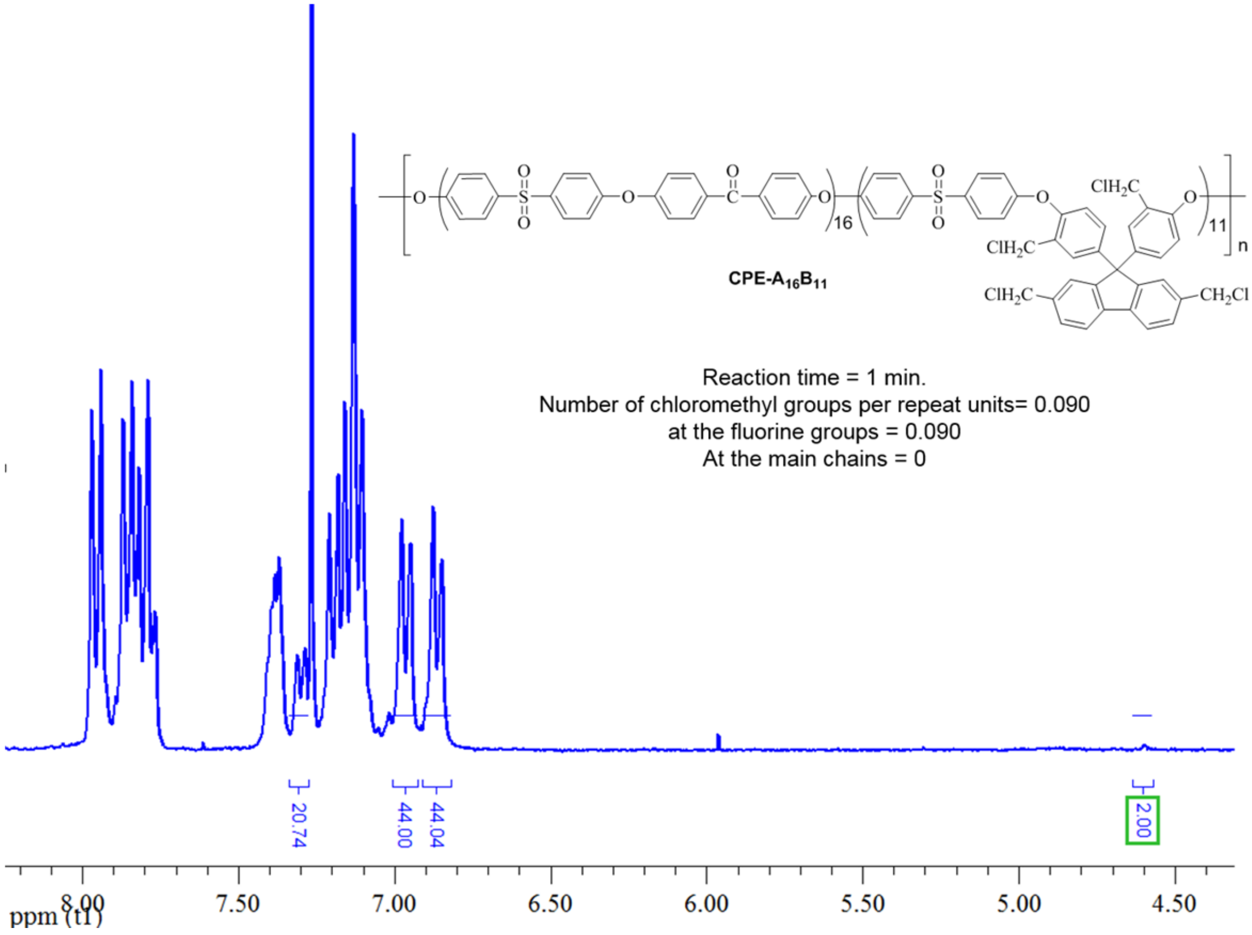

Supplementary Figure 66. ^{1}H NMR Spectra of CPE-$A_{16}B_{11}$ (**3g**).

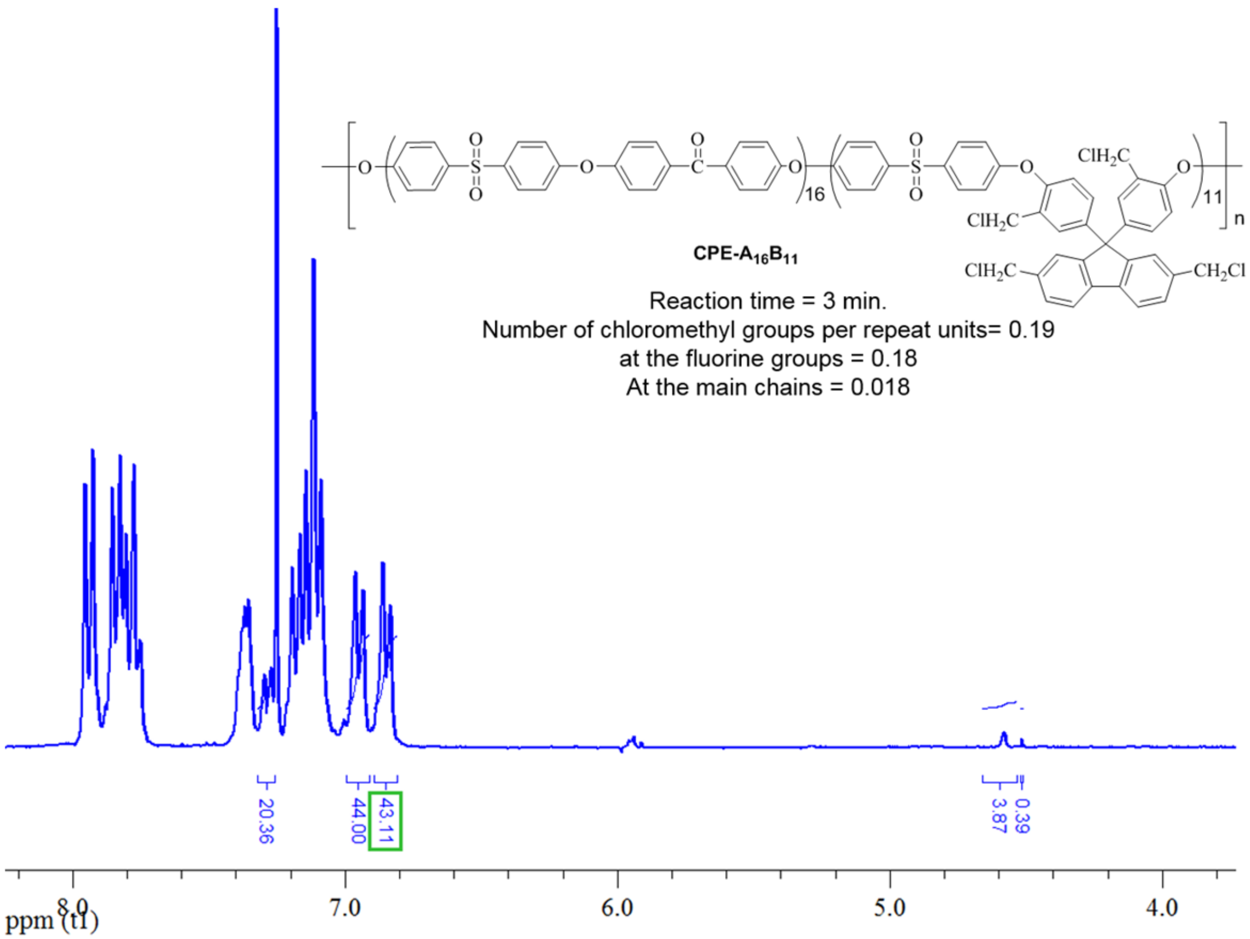

Supplementary Figure 67. ^{1}H NMR Spectra of CPE-$A_{16}B_{11}$ (**3h**).

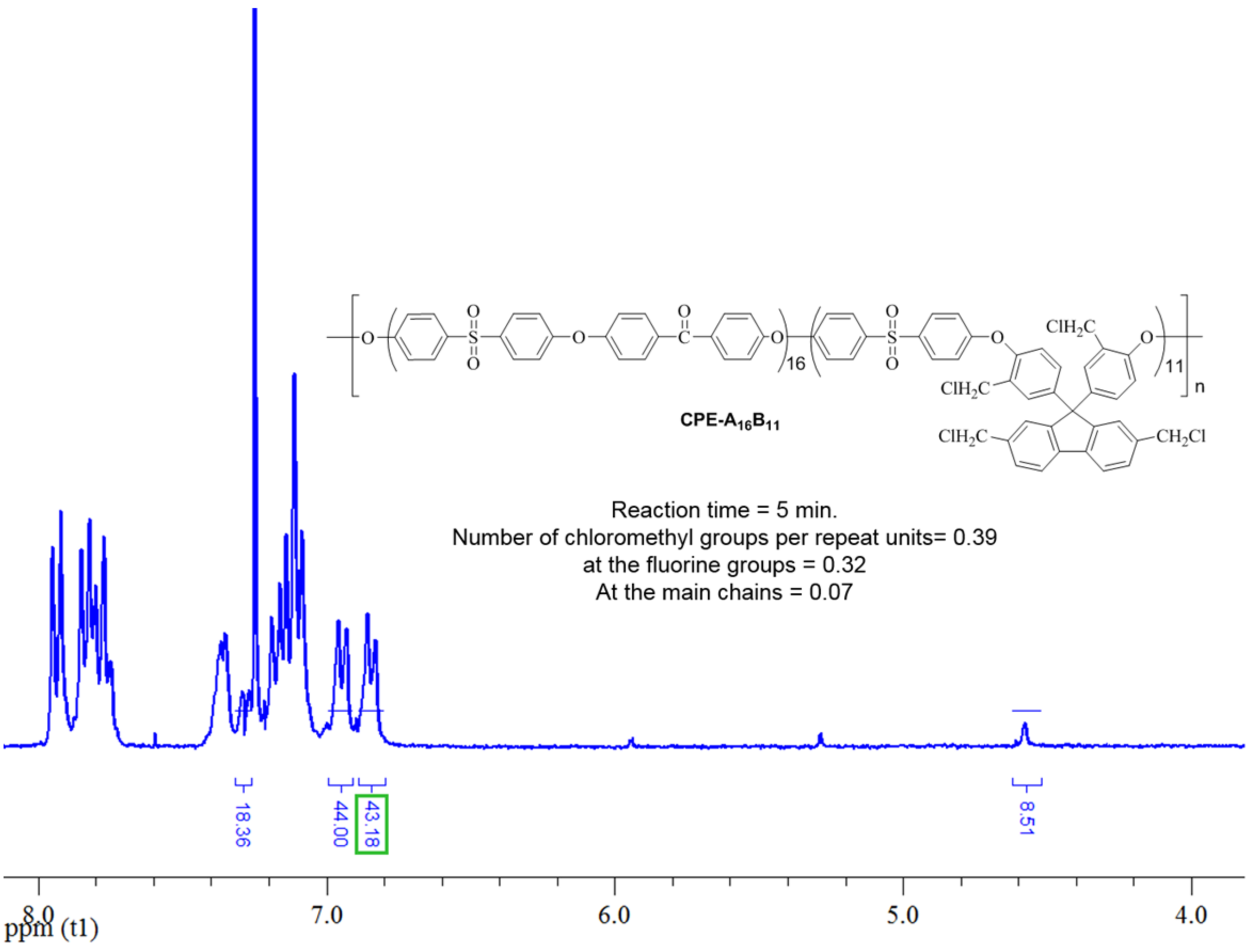

Supplementary Figure 68. ^{1}H NMR Spectra of CPE-$A_{16}B_{11}$ (**3i**).

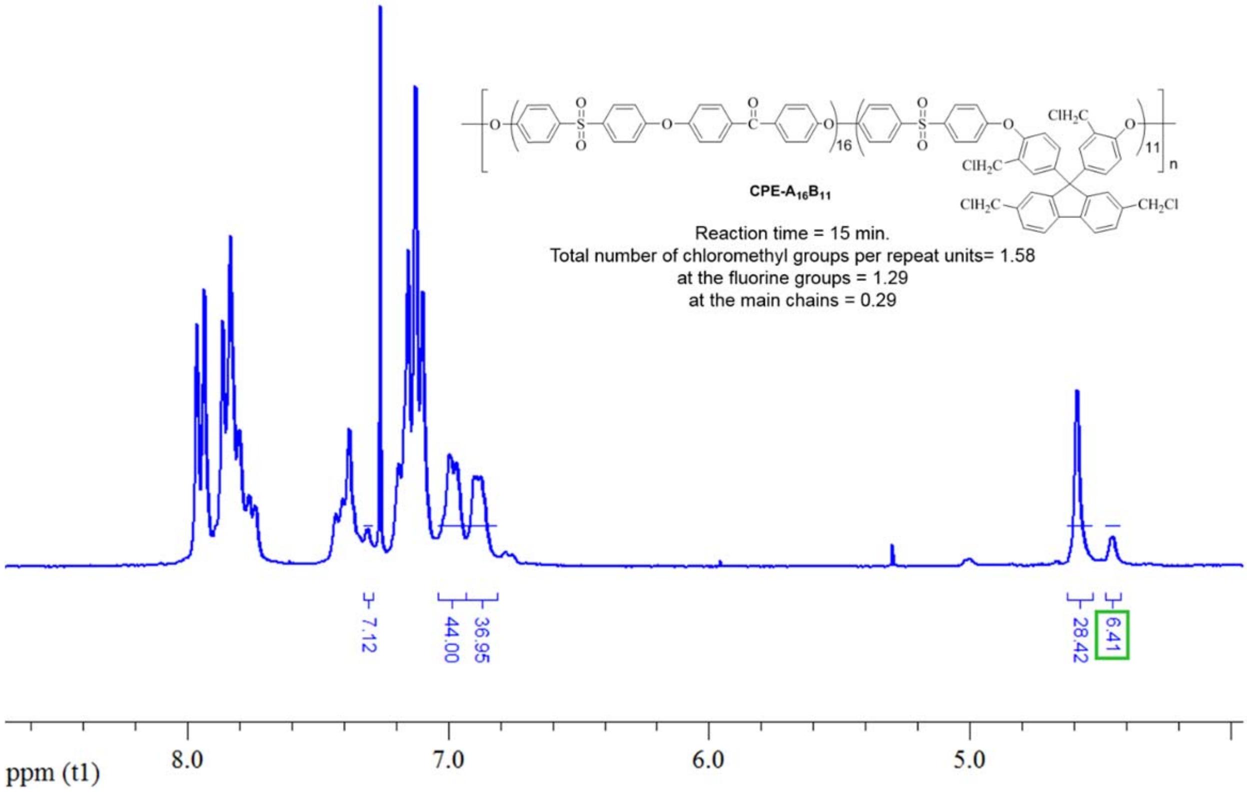

Supplementary Figure 69. ^{1}H NMR Spectra of CPE-$A_{16}B_{11}$ (**3j**).

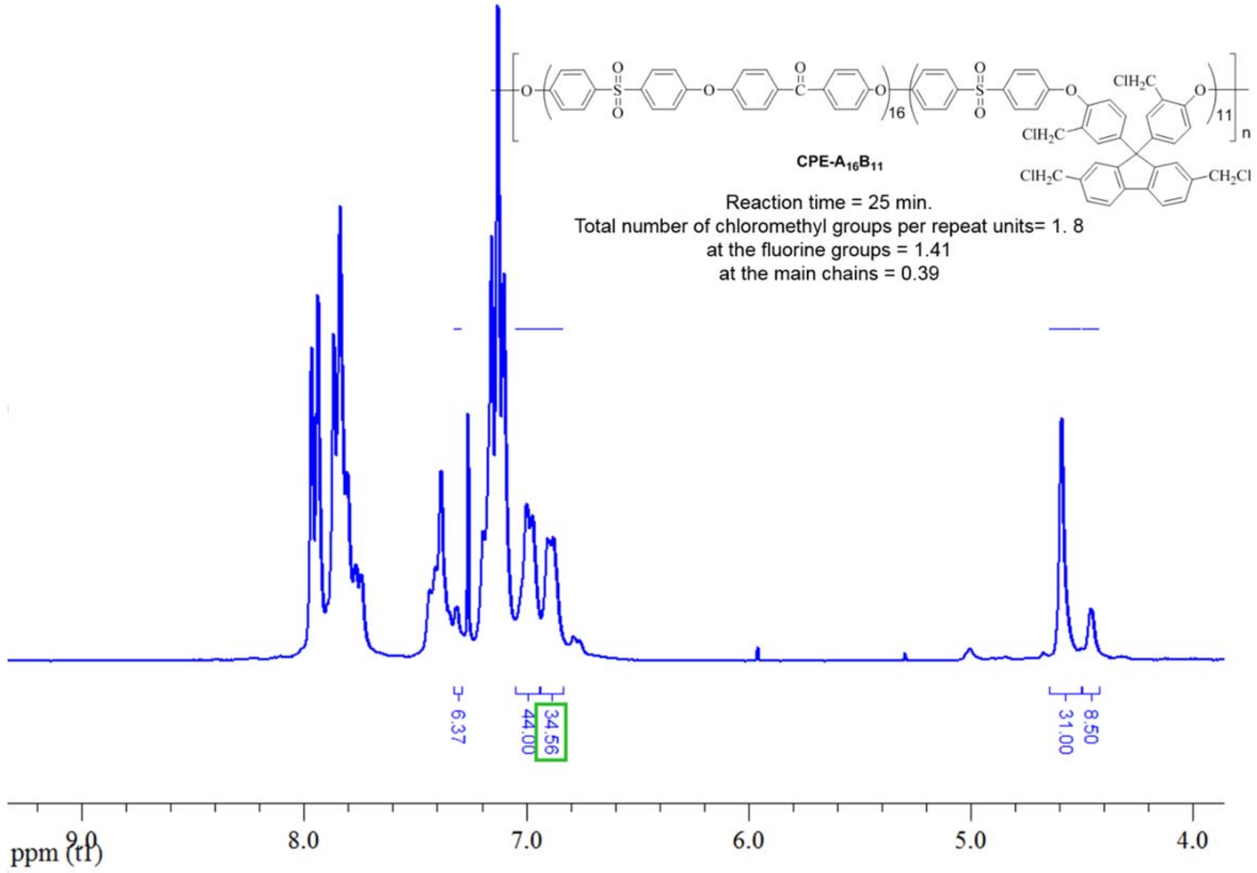

Supplementary Figure 70. ^{1}H NMR Spectra of CPE-$A_{16}B_{11}$ (**3k**).

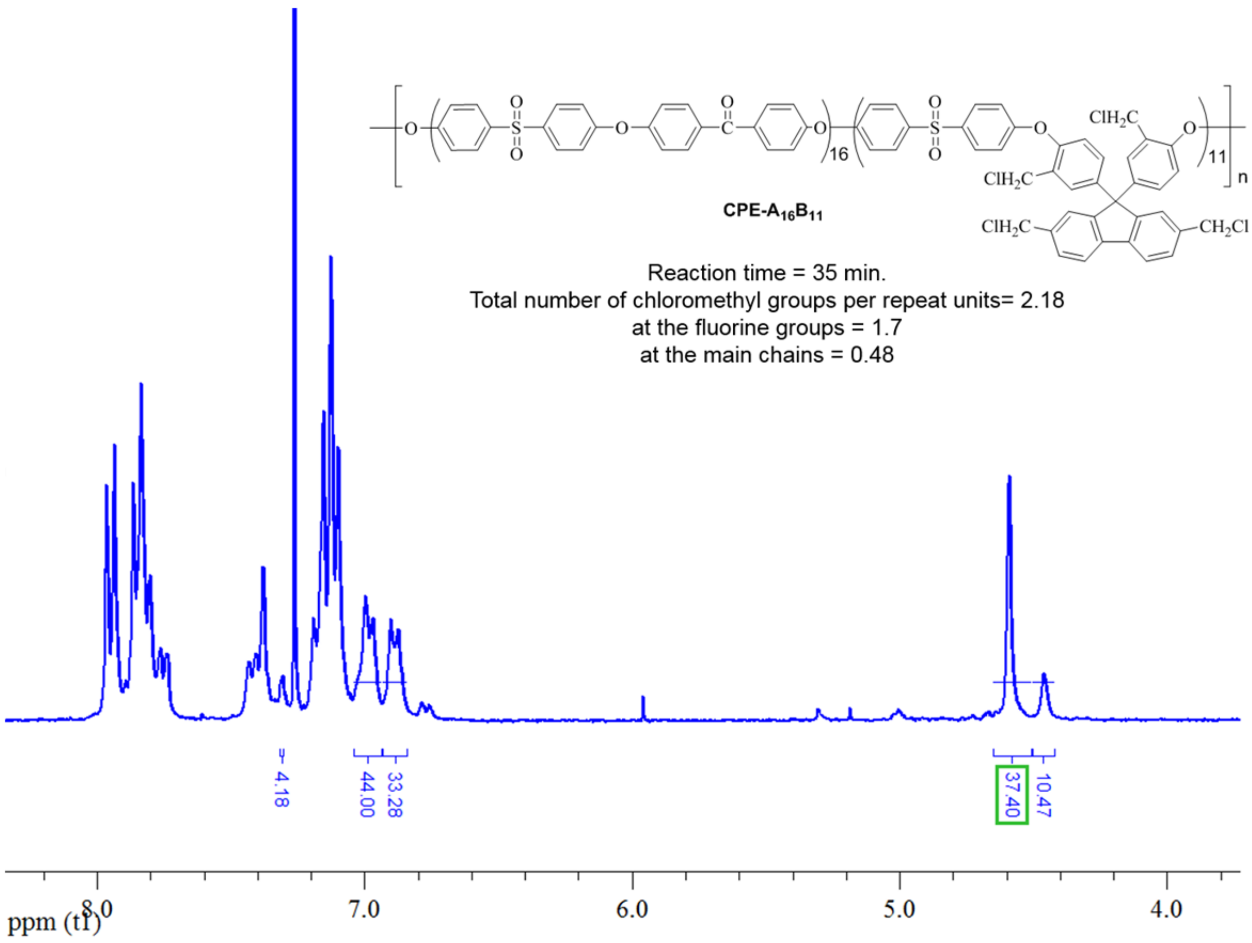

Supplementary Figure 71. ^{1}H NMR Spectra of CPE-$A_{16}B_{11}$ (**3l**).

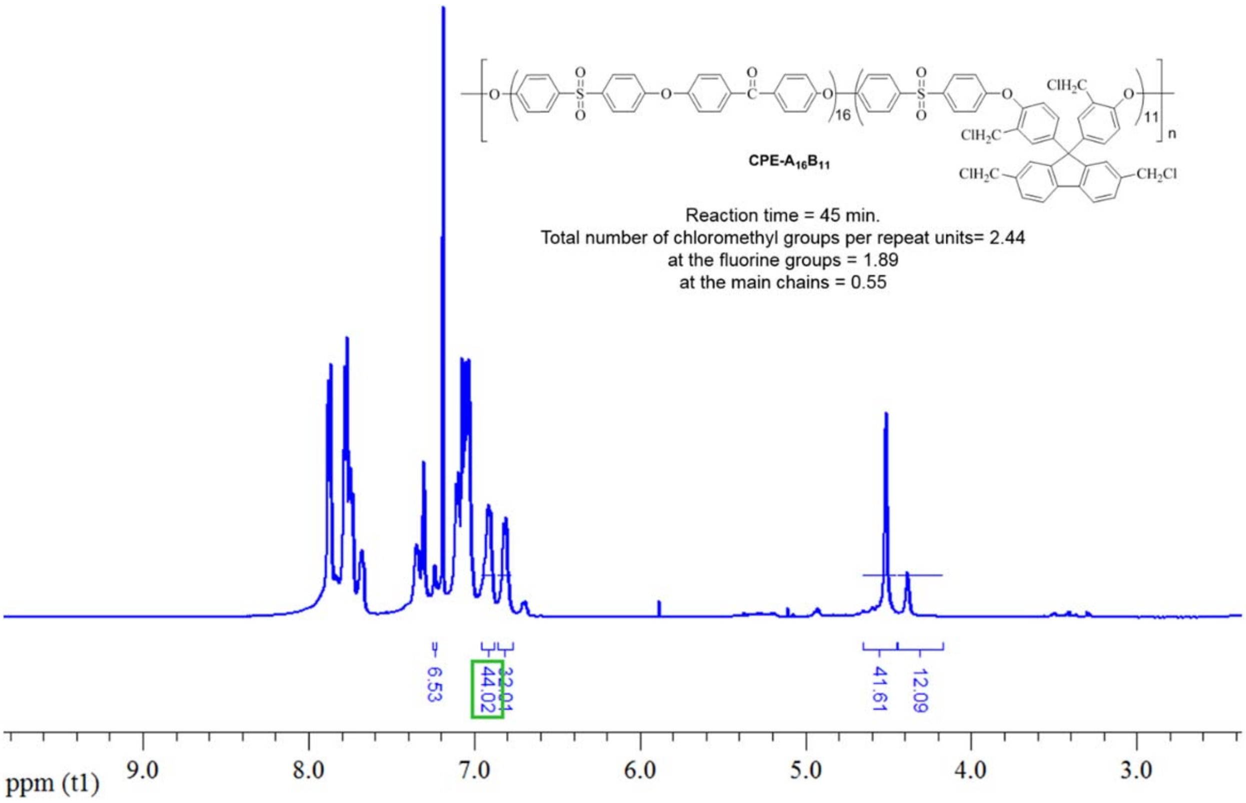

Supplementary Figure 72. ^{1}H NMR Spectra of CPE-$A_{16}B_{11}$ (**3m**).

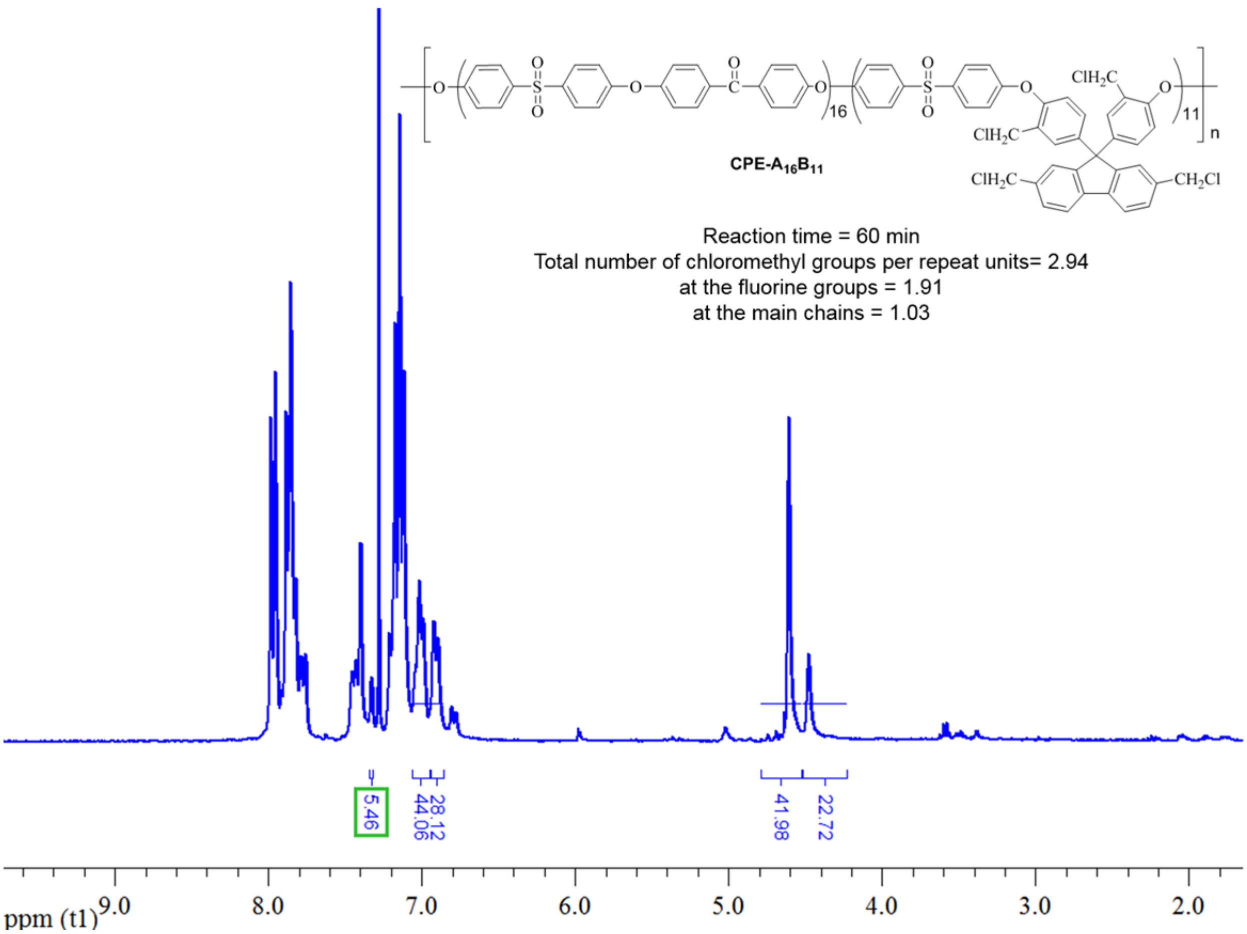

Supplementary Figure 73. ^{1}H NMR Spectra of CPE-$A_{16}B_{11}$ (**3n**).

Supplementary Tables

Supplementary Table 1: Optimization procedure for continuous-flow synthesis of CMME in a capillary reactor

Entry	Hexanoyl chloride flow rate ($\mu Lmin^{-1}$)	Dimethoxy methane flow rate ($\mu Lmin^{-1}$)	Retention time (min)	CH_3OCH_2Cl Yield (%)[a]
1	100	90	1.8	17
2	70	60	2.7	41
3	50	40	3.9	66
4	40	30	5.0	80
5	36	22	6.0	98
6[b]	36	22	6.0	11
7[c]	36	22	6.0	90
8[d]	36	22	6.0	81

Reaction condition: length 5 m, id 300 μm, volume 352 μm, 40 psi BPR, and 55 ^{o}C temperature [a]isolated product distilled by batch process and yield is based on NMR, [b]without BPR, [c]with 75 psi BPR, [d]40 psi BPR, and 65 ^{o}C temperature.

Supplementary Table 2: Reaction optimization of phenol group protection by reaction with the purified CMME

Entry	Phenol Flow rate (μLmin^{-1})	DIPEA Flow rate (μLmin^{-1})	Temp. (^{o}C)	Retention time R_2 (min)	BPR 40 psi	Yield (%)$^{(a)}$
1	180	37	50	4.7	Yes	12
2	180	37	40	4.7	Yes	22
3	180	37	30	4.7	Yes	32
4	180	37	RT	4.7	Yes	42
5	180	37	RT	4.7	No	99
6	200	37	RT	4.7	No	91

Reaction condition: Phenol concentration 1 M in DCM, Molar ratio of phenol:DIPEA:CMME at T_2 was 1:1.15:1.15, room temperature (~25 ^{o}C). (a) yields are based on NMR analysis.

Supplementary Methods

1. General Information

CAUTION: Chloromethyl methyl ether (CMME) is an OSHA registered carcinogen and is listed as an extremely hazardous substance by the EPA and the European Community.[1] Exercise due caution to zero exposure of CMME compound.

1.1. Materials

PTFE (id = 500 μm) tubing, T-junction, was purchased from Upchurch Scientific. Co.; Silicon wafer (P/100, dopant: boron) was purchased from Semi-Materials Co., Ltd.; HF solution (48-51 wt%) was purchased from Avantor Performance Materials, Inc; H_2O_2 solution (30 wt%) and ammonia solution (28-30 wt%) were purchased from Samchun Pure Chemical Co. Ltd; AZ 1512 positive photoresist was purchased from AZ Electronic Materials, PDMS (Sylgard 184) was purchased from Dow Corning, Polyvinylsilazane (HTT 1800) was purchased from Clarient. All organic solvents and reactants from Sigma-Aldrich or Alfa Aesar chemicals were used as received with no further purification. For experiments the deionized water (18.2 mS conductivity) was used.

2. Procedure for in situ generation, and separation of CMME chemicals

2.1. Procedure for in situ generation of CMME

Hexanoyl chloride was taken in one syringe and dimethyl ether was taken in another syringe (Supplementary Figure 1). Two reactants were introduced through T-mixer (T_1) in a flow rate molar ratio of 1:1 to maintain the stoichiometry (Table 1), and then passed through a PTFE tubing (id = 300 μm, length = varied) for the generation of CMME. As mentioned in

Supplementary Table 1 various reaction parameters (retention time, temperature, pressure) were regulated to optimize reaction performance. Eventually, 40 psi back pressure regulator (BPR), 6 min retention time at 55 °C generated the best yield 98% of CMME production (Supplementary Table 1, Entry 5) with no detection of carcinogenic BCME (bis-(chloromethylmethyl ether)) side product. But the increased reaction temperature to 65 °C produced the (bis-(chloromethylmethyl ether)) in reaction medium.

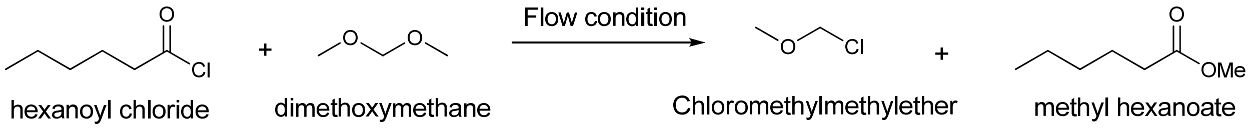

2.2. Continuous-flow separation of the generated CMME

In situ generated CMME was continuously separated by newly designed membrane-free SiNWs microseparator (Figure 1). The SiNWs microseparator was fabricated by following steps:

2.2.1. Fabrication of silicon nanowires (SiNWs) embedded microseparator

Step 1. Patterning of protective AZ photoresist layer on Si

Boron doped p-type silicon (100) wafers) was spin-coated by AZ1512 at 500 rpm for 5 sec and subsequently 2500 rpm for 30 sec to obtain average thickness of 50 µm. The AZ photoresist spin-coated silicon wafer was kept in an oven at 125 °C for 10 min to dry the solvent. The photo-masks (Supplementary Figure 2 right) were kept over soft baked silicon wafer and then irradiated with ultra-violet light (intensity 4.5 $mWcm^{-2}$) for 10 min. The UV treated silicon wafer was post-baked at 95 ^{o}C for 60 sec and kept in AZ developer for 5 min, washed with water and dried by blowing air.

Step 2. Preparation of SiNWs pattern

Oxide layer of boron doped p-type Si (100) wafer was removed by immersing in 1% HF aqueous solution for 1 min., washed with H_2O, and dried with N_2 blow. AgNO3 (5 mM) for 20 sec ~ 5 min to load Ag catalyst. The Ag-coated Si wafer was washed with water, dried with N_2 blow and etched by immersing in the mixture of 40 mL of 10 % HF solution and 0.544 gm of 30 % H_2O_2 (0.4 M) for 30 min ~ 6 hr. The obtained SiNWs wafer was washed with water and dried with N_2 blow.

Step 3. Fabrication of superamphiphobic SiNWs pattern

The etched SiNWs wafer was immersed in a mixture of 30 mL water, 0.1 mL TEOS and 0.1 mL hydrogen chloride solution at 70 ^{o}C for 5 hr to decorate silica nanoparticles on SiNWs surface. Silica nanoparticle coated SiNWs were washed with water and dried under 80 ^{o}C, finally dried sample was washed with SU-8 developer to remove photoresist. Finally, silicon nanowire with cone-shaped morphology was treated by trichloro(1H, 1H, 2H, 2H-perfluorooctyl)silane for providing chemical resistivity and hydrophobicity of nanowires.

In detail, the outstanding superamphiphobic property was tuned by varying the silver catalyst loading time (t_{Ag}), chemical etching time (t_{etch}) and SiO_2 decoration concentration (C_{TEOS}) under the fixed etchant concentration to optimize the NWs density and morphology. Silver catalyst amount was varied by tuning t_{Ag} from 20 sec to 5 min (Supplementary Figure 3). Longer loading times of silver catalyst caused denser deposition of silver, which lead to formation of more scattered SiNWs (Supplementary Figure 4). Upon less than 2 min of t_{Ag}, relatively high density SiNWs maintained vertically aligned morphology. When t_{Ag} was further extended up to 5 min, the SiNWs density with high aspect ratios became aggregated due to capillary force, forming a cone-shaped SiNWs morphology for the superamphiphobic surface property[2, 3]. In case over 5 min of t_{Ag}, the SiNWs partly started detaching from the substrate. Secondly, the longer t_{etch} in range 0.5~6 h generally produced higher etched fraction and longer NW height. When t_{etch} was 4 h or longer, the cone-shaped SiNWs morphology gave the highest DMSO contact angle (CA) (Supplementary Figure 4). Thirdly, when decorated the SiNWs surface by SiO_2 nanoparticles formed by sol-gel process of C_{TEOS} from 8 to 40 mM, smooth surface of the SiNW was gradually nanotextured to form a roughened morphology with re-entrant nanostructures (Supplementary Figure 5). At here, the SiO_2 nanotexturing on the smooth surface of cone-shaped SiNWs bundles

with micron scale intervals formed a hierarchically structured surface that considerably enhances the superamphiphobicity. The chemical stability was measured with CA changes after applying chemical stress by dipping the 2 cm by 2 cm SiNWs specimen into DMSO solvent for 24 hr or placing them in a desiccator with either HCl or NH_3 solution, which generates vapors, for 24 hr. The thermal stability was measured with CA changes after annealing the 2 cm by 2 cm SiNWs specimen at 300 ^{o}C for 1 hr in air (Supplementary Figure 6). Eventually, it indicated that the desirable superamphiphobic surfaces with highest liquid-repellency behavior (water CA 164°, DMSO CA 155°, methyl hexanoate 105.5 ^{o} (Supplementary Figure 7)) was achieved by optimizing three parameters of silver catalyst loading time at 5 min, chemical etching time for 4 h and SiO_2 decoration concentration at 24 mM TEOS.

Step 4. Fabrication of chemically resistant PDMS channel

PDMS channel was fabricated by following procedure.[4] Silicon wafer was spin-coated with SU-8-25 at 500 rpm for 5 sec and then at 3000 rpm for 30 sec to get average thickness of 15 µm. The spin-coated silicon wafer was kept in an oven at 65 °C for 2 min and then at 95 °C for 5 min for soft baking. The photo-masks (Supplementary Figure 2 left) were kept over soft baked silicon wafer and then irradiated with ultra-violet light for 10 min. The UV treated silicon wafer was post baked at 65°C for 1 min and then 95°C for 5 min. The post baked silicon wafer was kept in a SU-8 developer for 5 min, washed with isopropyl alcohol and dried by blowing air. The patterned silicon master was kept in a petridish, and Sylgard 184 resin and its curing agent as a precursor resin of PDMS were mixed at 10:1 ratio. After degassing the resin mixture was poured over the silicon master and kept in an oven at 60 °C for 3 h. The cured PDMS molds

were peeled off and punctured with needle for tubing connections. The cured PDMS channel was treated by oxygen plasma to activate the surface. Polyvinylsilazane (PVSZ) with 1 wt% 2,2-dimethoxy-2-phenyl-acetophenone photoinitiator was spin-coated at 2000 rpm for 40 sec onto the surface of the oxidized PDMS.

Step 5 & 6. Membrane-free SiNWs microseparator by bonding both resistant PDMS channel and SiNWs embedded channel

The PVSZ coated PDMS layer was then gently placed onto top of silicon nanowire pattern by aligning both channels with care, a sequential UV exposure for 10 min and heating at 150 ^{o}C for 4 h was finally applied to stabilize the bonding between PDMS channel and silicon nanowire pattern channel.

2.3. Typical procedure to separate CMME by distilling the mixture

The CMME was generated in a PTFE capillary microreacrtor by 6 min of residence time at 55 ^{o}C, as aforementioned. As shown Supplementary Figure 1, the newly designed membrane-free SiNWs microseparator was serially assembled with PTFE capillary microreactor by using T-junction and BPR. The SiNW microseparator inlet was connected with out-flowing stream of CMME and methylhexanoate (Supplementary Figure 9). With the help of the PTFE tubing and SiNWs microseparator streams were allowed to pass through the heating bath (varied temperature). The separation efficiency depended on temperatures (50-60 ^{o}C), dimension of microseparator (SiNWs height, diameter, SiO_2 nm deposition, trichloro(1H, 1H, 2H, 2H-perfluorooctyl)silane coating, PDMS height), and back pressure regulator. Eventually, the best CMME separation yield was achieved at 60 ^{o}C for 3 min retention time in the SiNWs

microchannel with unbalanced height of PDMS (15 μm) and SiNWs channel (75 μm). Superamphiphobic SiNWs pattern allowed to permeating only volatile CMME (boiling point 55-57 ^{o}C) with no wetting to liquid phase of organic ester (methyl hexanoate) with high boiling point. The separated CMME was condensed when passed through the capillary zone immersed at the room temperature water bath, and carefully collected the CMME liquid in a closed flask at production rate 17.5 μLmin^{-1} and used for NMR analysis. Liquid phase outlet was connected with 5 psi BPR for making pressure difference between liquid phase and gas phase. Finally, liquid phase stream were quenched with water to make zero exposure of remained CMME in liquid phase.

3. Typical procedure for optimization of phenol group protection

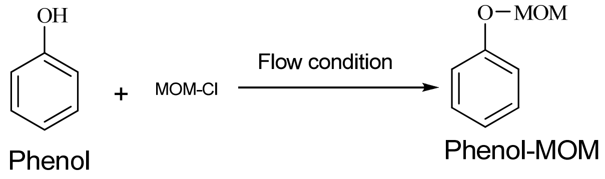

A 1.0 M solution of substituted phenol in DCM (flow rate: 180 mLmin^{-1}) and excess DIPEA reactant (flow rate: 37 μLmin^{-1}) were introduced to a T-mixer (T_1) of molar ratio of 1:1.15 to maintain reaction stoichiometry (Supplementary Table 2), and then passed through a PTFE tubing (id = 500 μm, length = 38 cm) for 20 sec retention time. Properly mixed phenol and DIPEA solution were connected by another T-mixer (T_2) and directly to outlet of the membrane-free SiNWs microseparator. Out-flowing CMME (17.5 μLmin^{-1}) and the substrate were controlled to become molar ratio 1:1.15 (Phenol:CMME). The three components (phenol, DIPEA and out-flowing CMME) were mixed through T-junction (T_2) (Supplementary Figure 10) and infused to PTFE tubing (id = 500 μm, length = 560.0 cm) for 4.7 min. Note that the tube length

was varied with different retention times of chloromethylation. Under the stable condition the product was collected and diluted with DCM and finally washed with sat. NH_4Cl solution to remove impurities. Collected product was analyzed by ^{1}H and ^{13}C NMR and GC-MS (data mentioned in below).

4. Typical procedure to quench the unreacted CMME and to separate the product in a continuous-flow manner

The phenol group protection setup as aforementioned in Fig S10 was connected to a quencher inlet for in situ decomposition of CMME and extraction removal of excess DIPEA/their salt. The saturated aq. NH_4Cl quenching solution was merged to the DCM based reaction mixture by T-junction to form aqueous-organic droplets (Supplementary Figure 11). Sufficient quenching and extraction from the above reaction mixture was observed at 1 $mLmin^{-1}$ flow rate of aq. NH_4Cl. It is generally known that the CMME was decomposed within 6 sec in water, and retention time around 30 sec in microfluidic system must be enough long to decompose completely[5].

To separate organic phase containing the wanted product from the aqueous impurity phase, the additional PFFE membrane embedded separator was added to end of the integrated microreactor system, as similarly reported (Supplementary Figure 12)[6, 7]. The separator was fabricated as following: polyethylene films (PE, 60 mm x 60 mm x 240 µm thickness) were manually punched to form a single groove with rectangular shape (8.2 mm x 35.5 mm) (Supplementary Figure 12). The 4-corners of two PE film were holed (1 mm diameter) to align the film patterns. Polytetrafluoroethylene (PTFE) membrane (Whatmann, 0.45 µm pore, 37 mm dia.) was sandwiched by two PE sheets with identical dimension of groove channels, and

aligned to each other by inserting metal pins through the holes at the film corners. Finally, the metal holder was tightly pressed by screw to seal the device with no leak.

A serial process of droplet formation, extraction and separation for purification of the phenol-MOM was conducted in droplet microfluidics equipped with the PTFE membrane microseparator, as explained in a step-wise manner at the below.

1: Formation of alternating organic-aqueous droplets: aq. NH_4Cl solution was introduced into the product mixture in DCM through T-junction (T_3).

2: Extraction and decomposition: the reaction wastes in the reaction mixture were gradually moved to aqueous droplet phase along a length of 320 cm capillary (id = 500 μm) for 30 sec flow.

3: Complete separation: the organic phase containing product could wet thin PFPE membrane and permeated to the opposite channel of the separator, whereas the waste containing aqueous phase did not wet the membrane and maintained at the original stream. The obtained product dissolved in DCM was analyzed by GC-MS, which showed a no DIPEA, CMME and other impurities as confirmed by absence of the corresponded peaks in NMR analysis (^{1}H and ^{13}C NMR spectra of 2a). Note that there was no workup such as washing the product with aq. NaOH, there was no need to be dried with Na_2SO_4 and no need for column chromatography.

Supplementary Movie 1: Continuous-flow separation of DCM organic droplets (no dye, containing phenol-MOM product) and aqueous droplets (with green dye, containing DIPEA +

DIPEA.HCl, + NH_4Cl + excess CMME) by a PFPE membrane microseparator. The clean separation of organic phase with no contamination by green dye was virtually demonstrated.

4.1. Typical procedure for continuous-flow protection of substituted phenol

Experimental method has been similar to the phenol group protection reaction as aforementioned.

4.2. Typical procedure for continuous-flow protection of substituted alcohol

As mentioned, a similar procedure has been adapted for alcohol group protection:

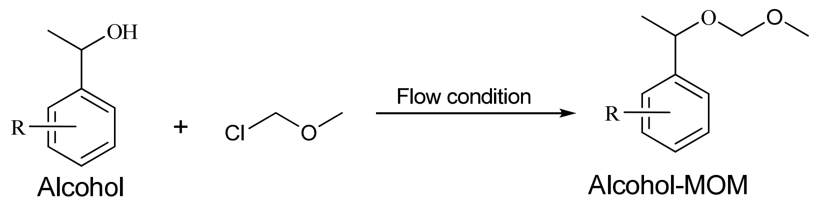

4.3. Typical procedure for continuous-flow protection of carboxylic acid group

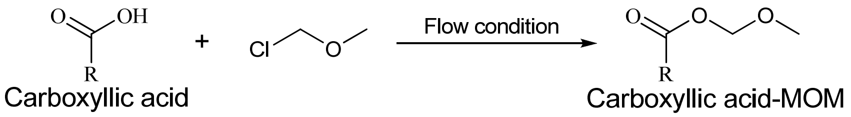

The phenol group protection was adapted for carboxylic acid group protection. The carboxylic acid compounds were dissolved by mixed solvent strategy.

4.4. Typical procedure for continuous-flow anionic functionalization of polysulphone by chlorometylation

4.4.1. Continuous chloromethylation of polysulphone

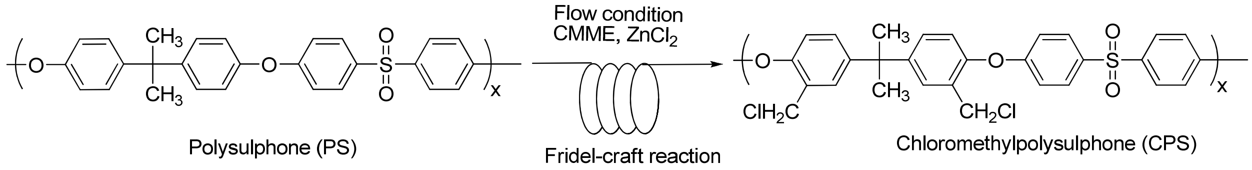

A solution of polysulphone (1.2 gm in 10 ml THF solution) was taken in one syringe and the solution of $ZnCl_2$ (0.05 M) in THF was taken in another syringe (Supplementary Figure 15). The two solutions were introduced to a T-mixer (T_1) in a flow rate (polysulphone solution = 160 $\mu Lmin^{-1}$, $ZnCl_2$= 14 $\mu Lmin^{-1}$), and then passed through PTFE tubing (id = 500 μm, l = 89 cm) to mix for 1 min residence time. Next the reaction mixture were connected to outlet of CMME purified microreactor at flow rate =17.5 $\mu Lmin^{-1}$ by another T-mixer (T_2) at 45 ^oC (Supplementary Figure 15). Three component mixture (polysulphone, $ZnCl_2$, CMME) was passed through a PTFE tubing (id = 500 μm, length = varied as mentioned in Supplementary Figure 15) for different retention times. The use of 40 psi BPR enhanced the degree of chloromethylation. Then controlled degree of chloromethylated polymer solution was connected with T-mixer (T_3), and THF solvent was added continuously (flow rate 200 $\mu Lmin^{-1}$) and again passed through PTFE tubing (id = 500 μm, l = 1 m) to dilute the polymer solution for 30 sec residence time. The properly diluted chloromethylated polymer solution was precipitated continuously by adding methanol (95% methanol and 5% water) solution (flow rate = 1 $mLmin^{-1}$) through T-mixer (T_4) and passed through a PTFE tubing (id = 1000 μm, length = 10 cm) to complete quenching of excess of $ZnCl_2$ and CMME during 3 sec residence time with no clogging problem. Finally, the chloromethylated polymer was washed several times with de-ionized water to determine degree of chloromthylation by NMR analysis after drying.

4.4.2. Typical procedure for continuous-flow anionic functionalization of multiblock copoly(arylene ether) by chloromethylation

4.4.2.1. Synthesis of fluorine-terminated oligomer (Block A_{16})

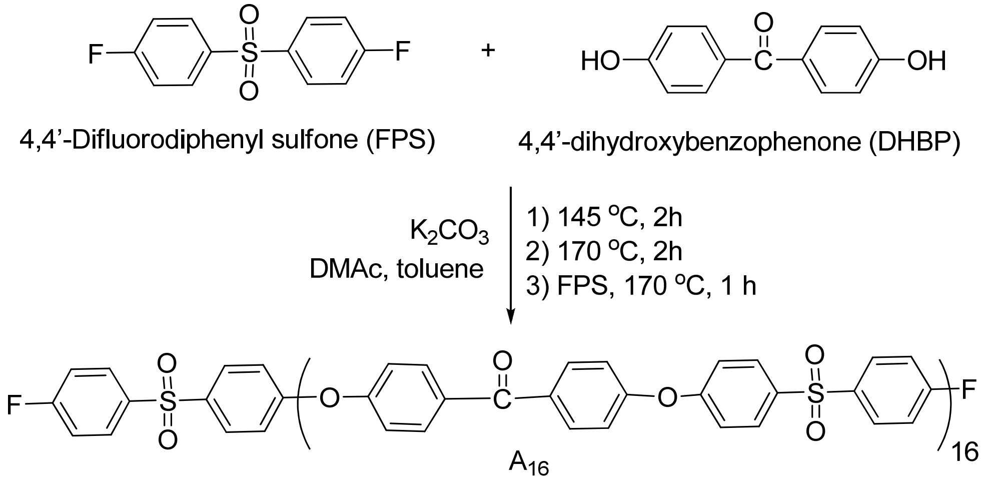

The hydrophobic fluorine-terminated oligomer was synthesized by following the reported method (as mentioned above).[8] In a 250 mL double necked round-bottomed flask, attached with a Dean-Stark trap, 4,4'-difluorodiphenyl sulfone (FPS) (2.50 gm, 9.83 mmol), 4,4'-dihydroxybenzophenone (DHBP) (1.98 gm, 9.25 mmol), and potassium carbonate (3.39 gm, 24.58 mmol) were mixed in 40 mL of dimethylacetamide (DMAc) and 10 mL of toluene under nitrogen atmosphere. The polymerization temperature was maintained at 150 °C for 3 hr and then the Dean-Stark trap was replaced by condenser. The polymerization temperature was maintained at 170 °C for additional 3 hr under nitrogen atmosphere. A light yellow, viscous mixture was obtained. A small amount of FPS (0.125 gm, 0.492 mmol) was added to the polymerization mixture to ensure fluorine end-capping oligomer. Again the polymerization mixture was stirred at 170 °C for 1 hr. The oligomer was obtained by precipitation of mixture in large excess of hot water while hot. The precipitated oligomer was collected by filtration and

was purified by repeated dissolution in DMAc and precipitated from hot water, twice and finally washed with methanol several times to get pure oligomer. Collected oligomer was dried under vacuum at 80 °C for 24 hr.

4.4.2.2. Synthesis of hydroxy-terminated oligomer (Block B_{11})

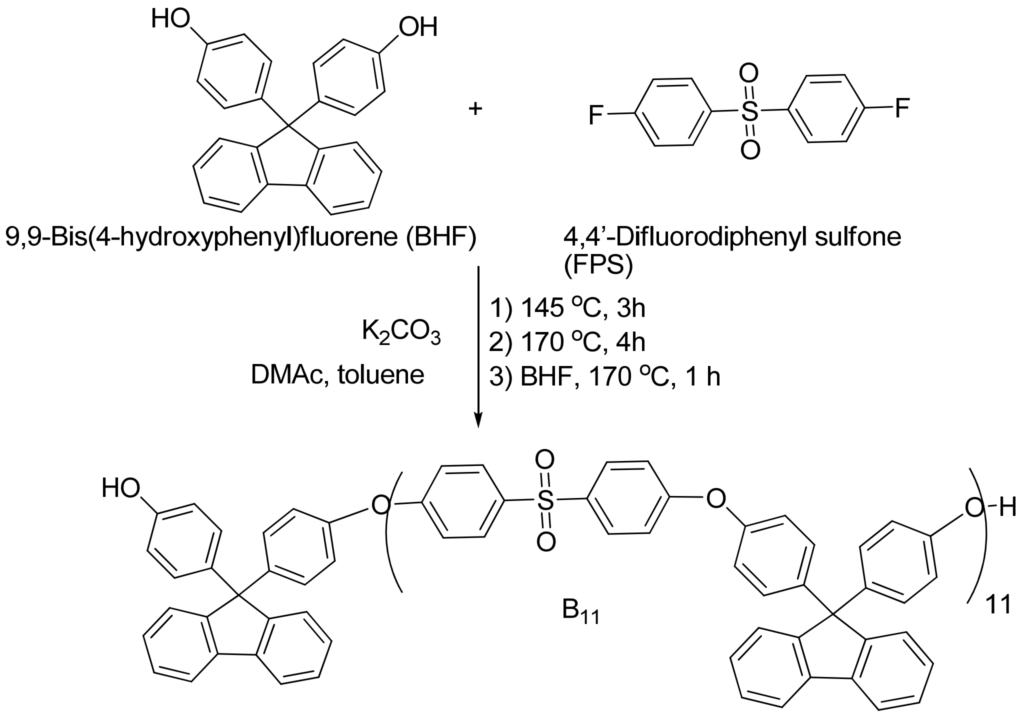

The fluorene-containing hydroxy-terminated oligomer was synthesized similarly to the fluorine-terminated oligomer (as mentioned above).[8] In a 250 mL double necked round-bottomed flask, attached with a Dean-Stark trap, FPS (1.25 gm, 4.92 mmol), 9,9-Bis(4-hydroxyphenyl)fluorene (BHF) (1.94 gm, 5.53 mmol) and potassium carbonate (1.91 gm, 13.83 mmol) were mixed with 20 mL DMAc and 5 mL toluene under nitrogen atmosphere. The polymerization temperature was maintained at 140 °C for 2 hr, then the Dean-Stark trap was replaced by condenser. The reaction temperature was then maintained at 165 °C for another 2 h under nitrogen flow. A small amount of BHF (0.097 gm, 0.277 mmol) was added to the reaction mixture to ensure

hydroxyl end-capping oligomer. After stirring for another 1 hr at 165 °C the mixture was precipitated in hot water while hot. The precipitated oligomer was collected by filtration and was purified by similar way as mentioned above for fluorine-terminated oligomer.

4.4.2.3. Synthesis of poly(arylene ether) multiblock copolymer

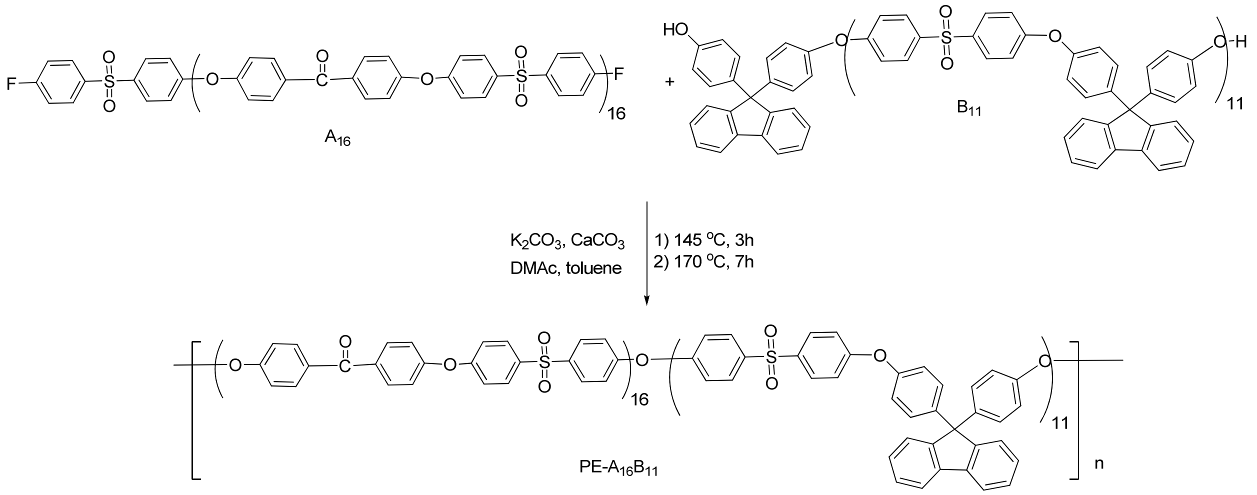

Block poly(arylene ether)s were synthesized by step-growth polymerization of fluorine-terminated oligomer and hydroxyl-terminated oligomer in 1:1 molar ratio.[8] In a 250 mL double necked round-bottomed flask, attached with Dean-Stark trap, fluorine-terminated oligomer (1.86 g, [-F] = 5.000 mmol), hydroxyl-terminated fluorene-containing oligomer (1.70 g, [-OH] = 5.000 mmol), potassium carbonate (0.864 g, 6.25 mmol) and calcium carbonate (6.26 gm, 62.50 mmol) were mixed in DMAc (25 mL) and toluene (6.25 mL) under nitrogen atmosphere. The reaction mixture was stirred at 145 °C for 3 h under nitrogen atmosphere. Then Dean-Stark trap was replaced by condenser and polymerization was continued at 160 °C for 5 hr. DMAc (20 mL) was added to the polymerization mixture at 160 °C and precipitated drop wise from excess of hot water while hot. The obtained polymer was purified by repeated dissolution in hot DMAc (25 mL) and precipitation from hot water, thrice. The product was obtained by filtration and

several times washed with deionized water and methanol and then dried at 80 ^{o}C under vacuum. To get narrower molecular weight distribution, polymer was dissolved in chloroform (80 mL) and precipitated from acetone (200 mL).

4.4.2.4. Chloromethylation of Poly(arylene ether) block copolymer

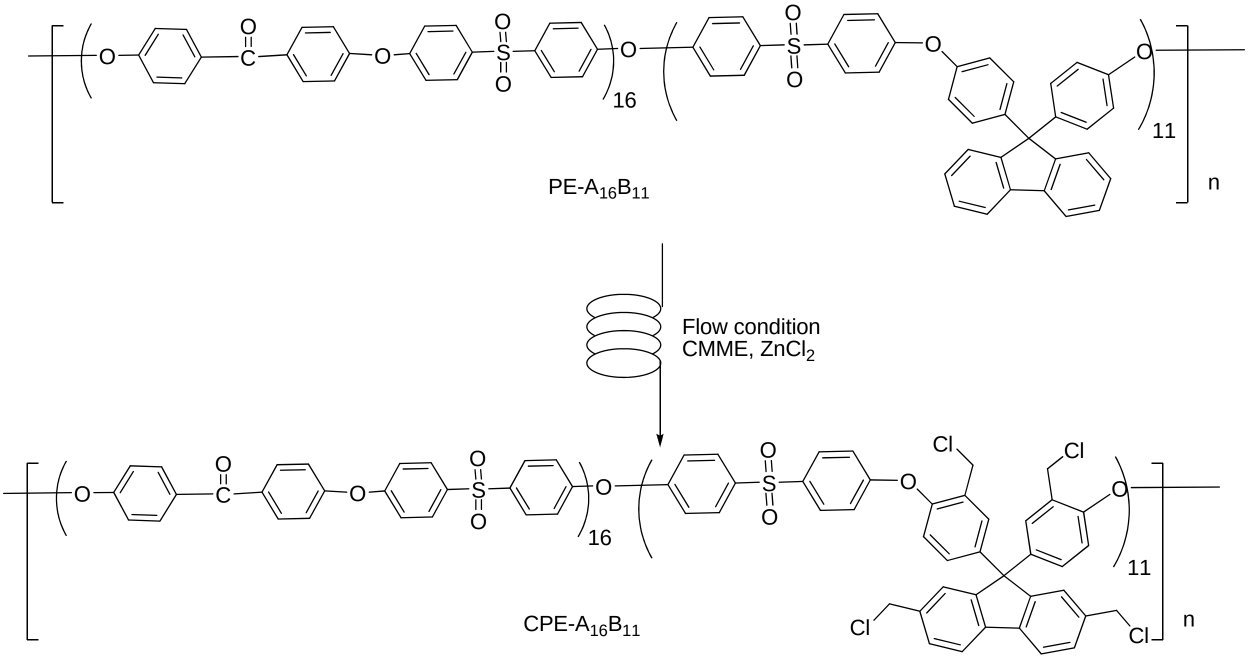

PE-$A_{16}B_{11}$ polymer (1.0 g, fluorine unit = 1.03 mmol) in 20 ml TCE solution was taken in one syringe and the solution of $ZnCl_2$ (0.25 M) in THF was taken in another syringe. The two solution were introduced to a T-mixer (T_1) in a flow rate (PE-$A_{16}B_{11}$ polymer solution = 101 $\mu Lmin^{-1}$, $ZnCl_2$= 11 $\mu Lmin^{-1}$), and then passed through PTFE tubing (id = 500 µm, l = 57 cm) to occur proper mixing of PE-$A_{16}B_{11}$ and zinc chloride solution during 1 min residence time. Next the reaction mixture was connected by another T-mixer (T_2) and out flowing in-situ generated CMME (flow rate =17.5 $\mu Lmin^{-1}$). Through the T-mixer (T_2) PE-$A_{16}B_{11}$, $ZnCl_2$, CMME could mix together (Supplementary Figure 19) and resulting solution was passed through a PTFE tubing (id = 500 µm, length = varied as mentioned below) under 45 ^{o}C temperature for different retention

times. The polymer solution was connected to T-mixer (T_3) by enhancing the degree of chloromethylation at 40 psi BPR. The diluted polymer solution by adding TCE solvent (flow rate 200 $\mu Lmin^{-1}$) was passed through PTFE tubing (id = 500 μm, length = 85 cm) to mix for 30 sec, and continuously precipitated by adding methanol solution (95% methanol + 5 water) (flow rate = 1 $mLmin^{-1}$) through T-mixer (T_4) and PTFE tubing (id = 1000 μm, length = 1 m) for complete quenching of excess $ZnCl_2$ and CMME for 3 sec residence time. Finally, the chloromethylated PE-$A_{16}B_{11}$ polymer was washed several times with de-ionized water and determined the degree of chloromethylation by NMR analysis after drying.

5. Characterization of all synthesized compounds

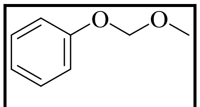

(2a)

(Methoxymethoxy)benzene (2a). Color: light yellow liquid, Mass (EI): m/z = 138 (M+); ^{1}H NMR ($CDCl_3$, 300MHz) δ: 3.51 (s, 3H), 5.21 (s, 2H), 7.01-7.09 (m, 3H), 7.30-7.35 (m, 2H), ^{13}C NMR ($CDCl_3$, 600MHz) δ: 55.9, 94.4, 116.3, 121.9, 129.4, 157.3.

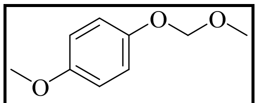

(2b)

1-Methoxy-4-(methoxymethoxy)benzene (2b). Color: yellow liquid, Mass (EI): m/z = 168 (M+); ^{1}H NMR ($CDCl_3$, 300MHz) δ: 3.49 (s, 3H), 3.8 (s, 3H), 5.18 (s, 2H), 6.57-6.69 (m, 3H), 7.10 (t, J = 8 Hz, 1H), ^{13}C NMR ($CDCl_3$, 150MHz) δ: 55.6, 55.8, 95.3, 114.6, 117.6, 151.3, 154.8.

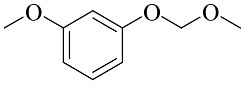

(**2c**)

1-Methoxy-3-(methoxymethoxy)benzene (2c). Color: yellow liquid, Mass (EI): m/z = 168 (M+); ^{1}H NMR ($CDCl_3$, 300MHz) δ: 3.49 (s, 3H), 3.79 (s, 3H), 5.17 (s, 2H), 6.56-6.68 (m, 3H), 7.17-7.23 (t, J = 8Hz, 1H), ^{13}C NMR ($CDCl_3$, 150 MHz) δ: 55.24, 55.97, 94.47, 102.62, 107.5, 108.42, 129.92, 158.52, 160.83.

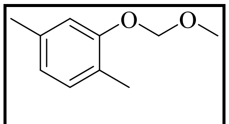

(**2d**)

2-(methoxymethoxy)-1,4-dimethylbenzene (2d). Color: yellow liquid**,** Mass (EI): m/z = 166 (M+); ^{1}H NMR ($CDCl_3$, 300MHz) δ: 2.31 (s, 3H), 2.41 (s, 3H), 3.58 (s, 3H), 5.27 (s, 2H), 6.87 (d, J= 8Hz, 1H), 6.98 (s, 1H), 7.10 (d, J = 7Hz, 1H), ^{13}C NMR ($CDCl_3$, 150MHz) δ: 15.9, 21.3, 55.9, 94.5, 114.8, 122.3, 124.2, 130.6, 136.7, 155.4.

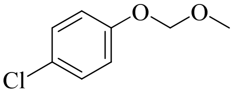

(**2e**)

1-chloro-4-(methoxymethoxy)benzene (2e). Color: yellow liquid**,** Mass (EI): m/z = 172 (M+); ^{1}H NMR ($CDCl_3$, 300MHz) δ: 3.50 (s, 3H), 5.17 (s, 2H), 7.0 (d, J = 9 Hz, 2H), 7.26 (d, J = 9 Hz, 2H), ^{13}C NMR ($CDCl_3$, 150MHz) δ: 56.0, 94.61, 117.6, 126.8, 129.3, 155.8.

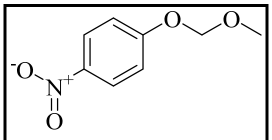

(2f)

1-(Methoxymethoxy)-4-nitrobenzene (2f). Color yellowish liquid**,** Mass (EI): m/z = 183 (M+); ^{1}H NMR ($CDCl_3$, 300MHz) δ: 3.48 (s, 3H), 5.25 (s, 2H), 7.0 (d, J = 9.3 Hz, 2H), 8.17 (d, J = 9.3 Hz, 2H), ^{13}C NMR ($CDCl_3$, 150MHz) δ:. 56.4, 94.3, 116.0, 126.7, 142.2, 162.2.

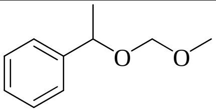

(2g)

(1-(Methoxymethoxy)ethyl)benzene (2g). Color: colorless liquid, Mass (EI): m/z = 166 (M+); ^{1}H NMR ($CDCl_3$, 600MHz) δ: 1.53 (d, J = 6.6 Hz, 3H), 3.4 (s, 3H), 4.59 (d, J = 6.6 Hz, 1H), 4.79 (d, J = 6.8 Hz, 1H), 4.8 (q, J = 6.5 Hz, 1H), 7.30-7.32 (m, 1H), 7.37-7.39 (m, 4H), ^{13}C NMR ($CDCl_3$, 150 MHz) δ: 23.6, 55.3, 73.8, 94.1, 126.3, 127.5, 128.4, 143.3.

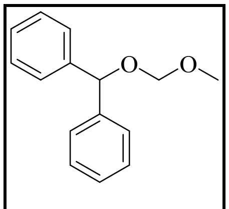

(2h)

((Methoxymethoxy)methylene)dibenzene (2h). Color: colorless liquid, Mass (EI): m/z = 228 (M+); ^{1}H NMR ($CDCl_3$, 600MHz) δ: 3.4 (s, 3H), 4.72 (s, 2H), 5.77 (s, 1H), 7.36 (d, J = 7.6 Hz, 4H), 7.39 (d, J = 7.2 Hz, 4H), ^{13}C NMR ($CDCl_3$, 150 MHz) δ: 55.6, 78.7, 94.0, 127.2, 127.5, 128.4, 141.8.

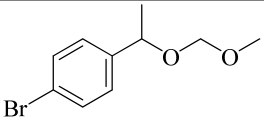

(**2i**)

1-Bromo-4-(1-(methoxymethoxy)ethyl)benzene (2i). Color: yellowish liquid, Mass (EI): m/z = 244 (M+); ^{1}H NMR ($CDCl_3$, 300MHz) δ: 1.43 (d, J = 6.5 Hz, 3H), 3.35 (s, 3H), 4.5 (dd, J = 10.8, 6.7 Hz, 2H), 4.7 (q, J = 6.5 Hz, 1H), 7.18-7.23 (m, 2H), 7.43-7.48 (m, 2H), ^{13}C NMR ($CDCl_3$, 150 MHz) δ: 23.5, 55.3, 73.1, 94.1, 121.2, 128.0, 131.5, 142.4.

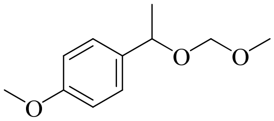

(**2j**)

1-Methoxy-4-(1-(methoxymethoxy)ethyl)benzene (2j). Color: colorless liquid, Mass (EI): m/z = 196 (M+); ^{1}H NMR ($CDCl_3$, 300MHz) δ: 1.45 (d, J = 6.5 Hz, 3H), 3.37 (s, 3H), 3.80 (s, 3H), 4.53 (dd, J = 6.7, 1.6 Hz, 2H), 4.7 (q, J = 6.5 Hz, 1H), 6.86 (d, J = 8.7 Hz, 2H), 7.25 (d, J = 8.5 Hz, 2H), ^{13}C NMR ($CDCl_3$, 150 MHz) δ: 23.5, 55.25, 55.28, 73.26, 93.86, 113.79, 127.65, 135.24, 159.0.

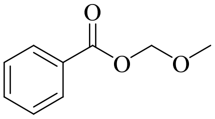

(**2k**)

Methoxymethyl benzoate (2k). Color: light yellow liquid, Mass (EI): m/z = 166 (M+); ^{1}H NMR ($CDCl_3$, 300MHz) δ: 3.51 (s, 3H), 5.46 (s, 2H), 7.39-7.44 (m, 2H), 7.51-7.57 (m, 1H), 8.02-8.06 (m, 2H), ^{13}C NMR ($CDCl_3$, 150MHz) δ: 57.6, 90.9, 128.4, 129.7, 129.9, 133.2, 166.0.

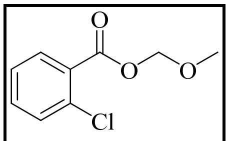

(2l)

Methoxymethyl 2-chlorobenzoate (2l). Color: yellow liquid, Mass (EI): m/z = 200 (M+); ^{1}H NMR ($CDCl_3$, 600MHz) δ: 3.59 (s, 3H), 5.51 (s, 2H), 7.33 (td, J = 6.3, 1.3 Hz, 1H), 7.44-7.49 (m, 2H), 7.89 (dd, J = 6.2, 1.6 Hz, 1H), ^{13}C NMR ($CDCl_3$, 150 MHz) δ: 58.03, 91.49, 126.63, 129.63, 131.18, 131.6, 132.83, 133.88, 165.13.

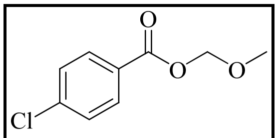

(2m)

Methoxymethyl 4-chlorobenzoate (2m). Color: yellowish liquid, Mass (EI): m/z = 200 (M+); ^{1}H NMR ($CDCl_3$, 600MHz) δ: 3.56 (s, 3H), 5.5 (s, 2H), 7.43 (d, J = 8.7 Hz, 2H), 8.0 (d, J = 8.7 Hz, 2H), ^{13}C NMR ($CDCl_3$, 150 MHz) δ: 57.8, 91.19, 128.33, 128.8, 131.15, 139.76, 165.18.

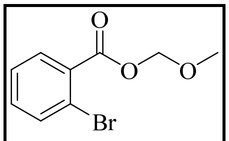

(2n)

Methoxymethyl 2-bromobenzoate (2n). Color: yellow liquid, Mass (EI): m/z = 243 (M+); ^{1}H NMR ($CDCl_3$, 600MHz) δ: 3.59 (s, 3H), 5.50 (s, 2H), 7.34-7.40 (m, 2H), 7.68 (dd, J = 6.5, 1.3 Hz, 1H), 7.85 (dd, 5.7, 1.9 Hz, 1H), ^{13}C NMR ($CDCl_3$, 150 MHz) δ: 58.1, 91.5, 121.7, 127.2, 131.5, 131.7, 132.8, 134.4, 165.5.

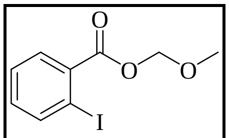

(2o)

Methoxymethyl 2-iodobenzoate (2o). Color: yellowish liquid, Mass (EI): m/z = 291 (M+); ^{1}H NMR ($CDCl_3$, 600MHz) δ: 3.61 (s, 3H), 5.52 (s, 2H), 7.18-7.2 (td, J = 5.8, 1.14 Hz, 1H), 7.43-7.45 (td, J = 5.8, 1.70 Hz, 1H), 7.88-7.90 (dd, J = 6.1, 1.6 Hz 1H), 8.03-8.04 (dd, J = 6.9, 1.03 Hz, 1H) ^{13}C NMR ($CDCl_3$, 150 MHz) δ: 58.2, 91.6, 94.1, 127.9, 131.9, 132.9, 134.6, 141.5, 165.8.

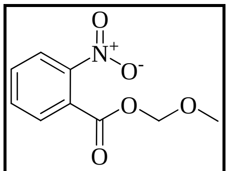

(2p)

Methoxymethyl 2-nitrobenzoate (2p). Color: light yellow liquid, Mass (EI): m/z = 211 (M+); ^{1}H NMR ($CDCl_3$, 600MHz) δ: 3.55 (s, 3H), 5.47 (s, 2H), 7.65-7.72 (m, 2H), 7.78 (dd, J = 6.0, 1.5 Hz, 1H), 7.91 (dd, 6.7, 1.2 Hz, 1H), ^{13}C NMR ($CDCl_3$, 150 MHz) δ: 58.2, 92.4, 123.9, 127.2, 129.9, 132.0, 132.9, 148.2, 164.9.

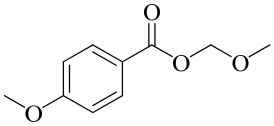

(2q)

Methoxymethyl 4-methoxybenzoate (2q). Color: light yellow liquid, Mass (EI): m/z = 196 (M+); ^{1}H NMR ($CDCl_3$, 300MHz) δ: 3.53 (s, 3H), 3.86 (s, 3H), 5.45 (s, 2H), 6.9 (d, J = 8.9 Hz, 2H), 8.02 (d, J = 8.9 Hz, 2H), ^{13}C NMR ($CDCl_3$, 150 MHz) δ: 55.4, 57.6, 90.6, 113.7, 122.2, 131.8, 163.6, 165.7.

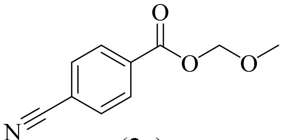

(2r)

Methoxymethyl 4-cyanobenzoate (2r). Color: yellowish liquid, Mass (EI): m/z = 191 (M+); ^{1}H NMR ($CDCl_3$, 300MHz) δ: 3.57 (s, 3H), 5.52 (s, 2H), 7.7 (d, J = 8.6 Hz, 2H), 8.1 (d, J = 8.6 Hz, 2H), ^{13}C NMR ($CDCl_3$, 150 MHz) δ: 58.0, 91.7, 116.7, 117.8, 130.2, 132.3, 133.7, 164.4.

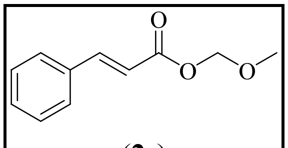

(2s)

Methoxymethylcinnamate (2s). Color: yellow liquid, Mass (EI): m/z = 192 (M+); ^{1}H NMR ($CDCl_3$, 150MHz) δ: 3.55 (s, 3H), 5.4 (s, 2H), 6.48 (d, J = 16 Hz, 1H), 7.42 (t, J = 3.2 Hz, 3H), 7.56-7.57 (m, 2H), 7.7 (d, J = 16 Hz, 1H), ^{13}C NMR ($CDCl_3$, 600 MHz) δ: 57.6, 90.5, 117.6, 128.2, 128.9, 130.5, 134.2, 145.7, 166.3.

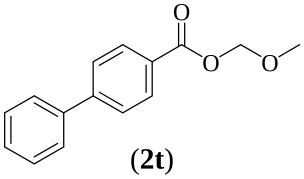

(2t)

Methoxymethyl biphenyl-4-carboxylate (2t). Color: Yellowish solid, Mass (EI): m/z = 242 (M+); ^{1}H NMR ($CDCl_3$, 600MHz) δ: 3.60 (s, 3H), 5.5 (s, 2H), 7.43 (d, J = 7.8, 1H), 7.65-7.67 (m, 2H), 7.70-7.72 (m, 2H), 8.18 (dd, J = 4.8 Hz, 1.8 Hz, 2H), ^{13}C NMR ($CDCl_3$, 150 MHz) δ: 57.7, 91.0, 127.1, 127.2, 128.2, 128.6, 128.9, 130.3, 139.9, 146.0, 165.9.

6. Supplementary References

1. Berliner, M.A. & Belecki, K. Simple, Rapid Procedure for the Synthesis of Chloromethyl Methyl Ether and Other Chloro Alkyl Ethers. *J. Org. Chem.* **70**, 9618-9621 (2005).
2. De Volder, M. & Hart, A.J. Engineering Hierarchical Nanostructures by Elastocapillary Self-Assembly. *Angew. Chem. Int. Ed.* **52**, 2412-2425 (2013).
3. Walia, J., Dhindsa, N., Khorasaninejad, M. & Saini, S.S. Color Generation and Refractive Index Sensing Using Diffraction from 2D Silicon Nanowire Arrays. *Small* **10**, 144-151 (2014).
4. Basavaraju, K.C., Sharma, S., Maurya, R.A. & Kim, D.-P. Safe Use of a Toxic Compound: Heterogeneous OsO4 Catalysis in a Nanobrush Polymer Microreactor. *Angew. Chem. Int. Ed.* **125**, 6867-6870 (2013).
5. *Acute Exposure Guideline Levels for Selected Airborne Chemicals:* The National Acadamic Press, Vol 3 (2013).
6. Sharma, S., Maurya, R.A., Min, K.-I., Jeong, G.-Y. & Kim, D.-P. Odorless Isocyanide Chemistry: An Integrated Microfluidic System for a Multistep Reaction Sequence. *Angew. Chem. Int. Ed.* **52**, 7564-7568 (2013).
7. Maurya, R.A., Min, K.-I. & Kim, D.-P. Continuous flow synthesis of toxic ethyl diazoacetate for utilization in an integrated microfluidic system. *Green Chem.* **16**, 116-120 (2014).
8. Tanaka, M. et al. Anion Conductive Block Poly(arylene ether)s: Synthesis, Properties, and Application in Alkaline Fuel Cells. *J. Am. Chem. Soc.* **133**, 10646-10654 (2011).
